# A snapshot of medical physics practice patterns

**DOI:** 10.1002/acm2.12464

**Published:** 2018-10-01

**Authors:** Kelly D. Kisling, Rachel B. Ger, Tucker J. Netherton, Carlos E. Cardenas, Constance A. Owens, Brian M. Anderson, Joonsang Lee, Dong Joo Rhee, Sharbacha S. Edward, Skylar S. Gay, Yulun He, Shaquan D. David, Jinzhong Yang, Paige L. Nitsch, Peter A. Balter, Diana L. Urbauer, Christine B. Peterson, Laurence E. Court, Scott Dube

**Affiliations:** ^1^ The University of Texas MD Anderson Cancer Center UTHealth Graduate School of Biomedical Sciences Houston TX USA; ^2^ Department of Radiation Physics The University of Texas MD Anderson Cancer Center Houston TX USA; ^3^ Department of Imaging Physics The University of Texas MD Anderson Cancer Center Houston TX USA; ^4^ Department of Biostatistics The University of Texas MD Anderson Cancer Center Houston TX USA; ^5^ Morton Plant Mease Health System Clearwater FL USA

**Keywords:** brachytherapy, external beam radiation therapy, quality assurance, radiation therapy, surveys

## Abstract

A large number of surveys have been sent to the medical physics community addressing many clinical topics for which the medical physicist is, or may be, responsible. Each survey provides an insight into clinical practice relevant to the medical physics community. The goal of this study was to create a summary of these surveys giving a snapshot of clinical practice patterns. Surveys used in this study were created using SurveyMonkey and distributed between February 6, 2013 and January 2, 2018 via the MEDPHYS and MEDDOS listserv groups. The format of the surveys included questions that were multiple choice and free response. Surveys were included in this analysis if they met the following criteria: more than 20 responses, relevant to radiation therapy physics practice, not single‐vendor specific, and formatted as multiple‐choice questions (i.e., not exclusively free‐text responses). Although the results of free response questions were not explicitly reported, they were carefully reviewed, and the responses were considered in the discussion of each topic. Two‐hundred and fifty‐two surveys were available, of which 139 passed the inclusion criteria. The mean number of questions per survey was 4. The mean number of respondents per survey was 63. Summaries were made for the following topics: simulation, treatment planning, electron treatments, linac commissioning and quality assurance, setup and treatment verification, IMRT and VMAT treatments, SRS/SBRT, breast treatments, prostate treatments, brachytherapy, TBI, facial lesion treatments, clinical workflow, and after‐hours/emergent treatments. We have provided a coherent overview of medical physics practice according to surveys conducted over the last 5 yr, which will be instructive for medical physicists.

## 1 INTRODUCTION

Surveys have been a useful tool in understanding and documenting the standard‐of‐care in various topics in radiotherapy physics.^1^, ^2^, ^3^, ^4^ Over the past 5 yr, there have been over 250 surveys distributed mainly through the medical physics listserv covering varying topics of interest to clinical physicists (e.g., simulation practices, use of image guidance, and clinical workflow). Many of these questions have been posed by practicing medical physicists on the listserv as they are inquiries that cannot be easily found in published works. While the results of these surveys are a valuable resource to the community, they are currently difficult to access. This information has not been formally analyzed or published before, and currently can only be accessed by searching through 5 yr of listserv emails to find the original survey link.

We aimed to make this valuable resource more accessible to the medical physics community by summarizing the results of the surveys that were of general interest for therapeutic medical physicists, had a reasonable number of responses, and were not vendor specific. This summary can serve as a convenient reference for the community to understand the actual practice patterns of their peers.

When reviewing and interpreting the results of these surveys, readers should keep in mind that these were not formal surveys as participation in the listserv and surveys was voluntary, so the participants do not represent a random sample of the population. Survey participation may be influenced by a variety of factors, and the data may not necessarily represent the entire medical physics community. The retrospective nature of this analysis was designed to provide preliminary idea of medical physics practices.

## 2 MATERIALS AND METHODS

The surveys used in this work were created by a single user (S.D.) through SurveyMonkey (SurveyMonkey, San Mateo, CA) and distributed via the MEDPHYS and MEDDOS list groups hosted by Wayne State University. These groups are primarily focused on practicing medical physicists and dosimetrists, respectively. The specific breakdown of demographics within these groups is not available. However, the MEDPHYS listserv is self‐described as “an international discussion list for the professional medical physicist and students in the field,” and the MEDDOS listserv is described similarly for professional medical dosimetrists. The MEDPHYS and MEDDOS listserv groups have over 6,000 and 1,500 members, respectively.

These surveys were conducted from February 6, 2013 to January 2, 2018. The specific questions were prompted by questions from members of the MEDPHYS listserv. The format of the surveys included questions that were multiple choice plus free response follow‐up questions or solely free response questions.

Surveys were first reviewed and included in further analysis if they met the following inclusion criteria: more than 20 responses, relevant to radiation therapy physics practice, not single‐vendor specific, and formatted as multiple‐choice questions (i.e., not exclusively free‐text responses). Although the results of free response questions were not explicitly reported, they were carefully reviewed, and the responses were considered in the discussion of each question. The remaining surveys were then divided into broad categories. The results of the surveys that met the inclusion criteria were then collated, and the most interesting points were summarized in the Results section. If the same survey was sent to multiple listserv groups, the results were combined to give one percentage in the Results section.

To estimate the uncertainty in the responses for each survey question, we calculated the simultaneous 95% confidence intervals for the multinomial proportions. This was computed with MultinomialCI (version 1.0) in R (version 3.4.4).^5^ The half‐widths of the confidence intervals are given as a percentage and are found in the tables in the Data S1.

## 3 RESULTS

**Figure 1:**
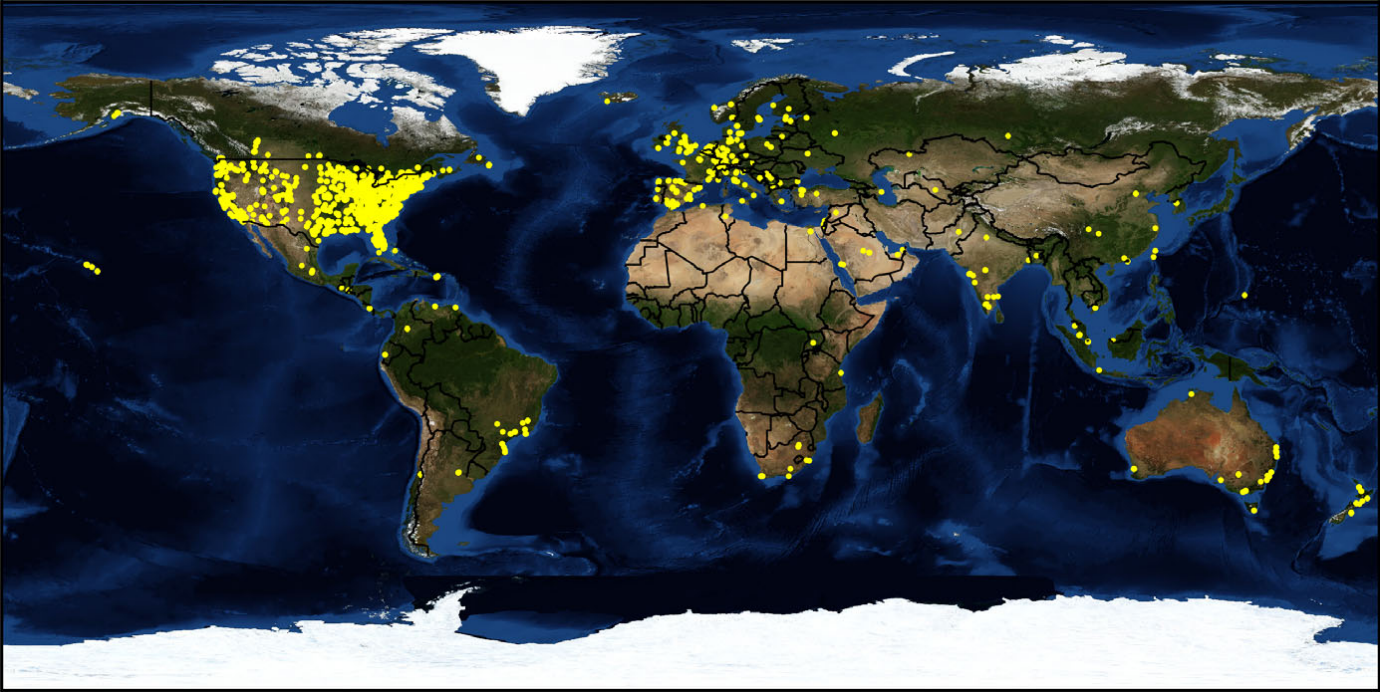
Geographical distribution of respondents. Geographical map showing all respondents who participated in the 139 selected surveys that were analyzed.

The results of 252 Surveys were available for initial review, of which 139 passed the inclusion criteria. The number of questions per survey ranged from one to nine with an average of four questions. The average number of responses to the questions in the 139 surveys was 63 (range: 5–183). The surveys with fewer than 20 responses had an identical survey on a different listserv, and the total responses in the combined surveys were above 20. Additionally, three additional free‐response surveys that had solely quantitative response (i.e., CT slice thickness or percent effort) were included. The geographical distribution of respondents is shown in Fig. 1. Eighty‐seven percent of responses were from North America. The tabulated results of all surveys that met the inclusion criteria are reported in the Data S1. The most salient points are summarized below.

### 3.A Simulation

There were several surveys addressing practices in CT simulation. Various CT slice thicknesses were reported (text responses), ranging from 2 to 5 mm for conventional radiotherapy and 0.6 to 2 mm for stereotactic body radiotherapy (SBRT).

The use of intravenous contrast for CT simulation is widespread, with use depending on the anatomic site. Sixty percent of respondents use contrast for head/neck scans, 38% for female pelvis, and 40% for male pelvis. Thirty‐eight percent of respondents never use contrast for CT simulation. When contrast is used, respondents ignore the contrast when planning (26%), use the noncontrast CT fused with contrast‐enhanced CT (42%), or override the contrast CT numbers (32%).

When considering 4D simulation for lung patients, 61% reported using the mean intensity projection (MIP) images to define the internal target volume (ITV) and 25% delineate gross tumor volumes (GTV) on each phase. Although MIP was the most popular choice, rigorous evaluation of the delineated volume through all phases was recommended by many text responses to prevent any missed volume, particularly for peripheral tumors. A small minority (6%) use “slow scans” as determined on a separate survey. The treatment plans are then planned using a free breathing scan (52%) or average CT (41%), whereas very few respondents (7%) override the density.

### 3.B Treatment planning

Surveys about treatment planning were diverse in nature. Ones of particular note were choice of software, beam naming convention, dose‐grid size, inclusion of prior irradiation, and use of hard wedges. Respondents have a wide range of treatment planning system and oncology information systems: Eclipse/Aria (Varian Medical Systems, Palo Alto, CA) (39%), Pinnacle (Phillips Medical Systems, Cleveland, OH)/Mosaiq (Elekta, Stockholm, Sweden) (35%), Eclipse/Mosaiq (11%), Pinnacle/Aria (4%), other/Mosaiq (8%) and other/Aria (3%). Beam naming conventions are split between anatomy‐based and machine‐based (32%/41%). Few were aware of published naming convention recommendations (text responses).^6^, ^7^, ^8^, ^9^, ^10^, ^11^ The dose‐grid size used varied between respondents, varying from 1 to 3 mm, with respondents often reporting the use of smaller dose grids for special procedures such as SBRT (text responses). The use of dose‐to‐water or dose‐to‐medium is approximately evenly split for both photon and electron planning, with 46% and 47% using dose‐to‐water for photons and electrons, respectively. Various ways to include prior irradiation in treatment plans were reported, including the use of specialized deformable image registration software to map the doses (26%), registration of old and new CT images in the treatment planning system (54%) and recalculating the old plan on the new CT after manually setting the isocenter (20%). This is usually performed by the dosimetrist (56%) or physicist (36%). Fifty‐five percent of users still used hard wedges. Reasons for maintaining hard wedges include the following: necessity because of the different orientation of the multileaf collimator (MLC) and dynamic wedge, to use together with field‐in‐field techniques, or dosimetrist preference (text responses).

When evaluating treatment target and normal tissue doses, the most common method (39%) was to manually extract metrics and tabulate them in a spreadsheet. The results of the treatment plan evaluation are usually stored as a file report in the patient's chart (85%).

### 3.C Electrons

The method of determining monitor units (MU) for electron treatments was a popular topic, appearing in three surveys over the years 2013, 2014, and 2016. In each of these, 14%, 24%, and 54%, respectively, reported using the MU from the treatment planning system, whereas the remainder used independent calculation. Many (85%) include the effect of tissue heterogeneities in the calculated dose. Sixty‐five percent of respondents present the isodose lines as absolute dose; the rest use relative dose. Based on the 2014 survey, institutions measure the output factor for each electron cutout individually (31%), have a library of premeasured cutout factors (32%), or rely on the dose calculation software (21%). Of those that measure output factors, they usually use an ion chamber in solid water (81%). These measurements are usually performed by physicists (text responses). The maximum source‐to‐surface distance (SSD) that respondents will use without output factor measurement ranges from 100 cm (22%) to 105 cm (29%) or 110 cm (46%).

### 3.D Linac commissioning and quality assurance (QA)

At acceptance, about half of respondents test for linac head leakage (53%). For commissioning, the vast majority of respondents would purchase a 3D beam scanning system (84%), rather than a 1D beam scanning system and 2D array, given the option. Respondents who preferred the 1D beam scanning system/2D array combination cited cost and efficiency as the reasons for their choice (text responses). Respondents who preferred 3D beam scanning systems often gave the following justifications: versatility, field‐size concerns/limitations of 1D/2D combination, as well as concerns about the use of array data for commissioning (text responses). In a separate survey, 25% of respondents use preconfigured beam profiles in their planning system, 47% use scanned profiles with asymmetry corrected (ie, centered and mirrored), and 28% use profiles without any asymmetry correction.

Most linacs are calibrated to 1 cGy/MU at depth of maximum dose for SSD setup (61%) or source axial distance (SAD) setup (29%). Other respondents reported the use of different reference depths (5 and 10 cm). Almost three‐quarters of clinics (73%) have adopted the TG‐51 addendum, 98% of which reported that this resulted in less than a 1% change in calibrated output.

The output of the linac is verified monthly using a variety of approaches. Most use ion chambers in a solid water phantom (76%), although ion chambers in a water tank are also popular (20%). In almost all responses, the baseline for secondary approaches is established annually with respect to the primary TG‐51 calibration (text responses). When the secondary system indicates a change in output, most respondents adjust the linac output, with some setting up their water tank to confirm or make the adjustment (text responses).

Monthly energy verification is usually performed using two depth measurements in either solid water (63%) or water (18%), with some institutions using planar measurements to verify flatness (and therefore energy) (10%). Almost half of respondents (45%) use the daily QA device results for their monthly flatness and symmetry measurements. Many different approaches are used when reporting symmetry, although almost half the respondents use central axis point difference (49%).

The frequency of constancy checks of electrometer‐chamber combinations varies from annual (45%), every 6 months (27%), monthly (19%), and never (8%). In the majority of cases this involves the intercomparison of two independent systems (62%).

### 3.E Setup and treatment verification

Patient setup surveys encompassed a large range of topics, from immobilization to image‐guided radiation therapy (IGRT). One common topic was the use of a table pad. Sixty‐two percent of respondents use a table pad for brain patients and 67% use table pads for “patients in pain.” Outside of this, for head and neck, lung, and pelvis patients, the majority do not use a table pad (52%, 76%, and 76%, respectively). Another topic was the use of sheets on top and/or sheets or thin pads below patients. Sixty‐four percent of respondents allow the use of a sheet or blanket over the treatment area, and 90% use sheets or thin pads below patients. Immobilization devices were also surveyed. To immobilize shoulders, a head/neck/shoulder mask is used 45% of the time. Other methods used to immobilize the shoulders include using wrist rope pulls with a foot board (18%), using both a mask and rope pulls (20%), and using shoulder suppression brackets (17%). For whole‐brain treatments, 100% of respondents use thermoplastic masks. Setup photos were reported to be used in the vault by 89% of respondents. For non‐IMRT fields, the majority of respondents record the SSDs on the first fraction (78%) and on a weekly basis (51%).

In a survey about fiducial markers, the majority of respondents reported that they use fiducial markers for IGRT (68%). Most of the fiducial markers used are gold (80%). The fiducials are usually “individual seeds” (69%) or coils (19%). During IGRT, the majority (68%) give therapists the authority to make shifts up to 1 cm at their own discretion, whereas some respondents allow up to 3 cm or any shift deemed necessary. If the shift is over the limit, most respondents reported that the physician input is required (80%), whereas less than half reported that the physicist is required (45%). Twenty‐nine percent of respondents reported that they have had a treatment event due to an inappropriate shift. When asked if they use daily IGRT for whole brain treatment, no respondents reported using daily IGRT.

Respondents generally seemed to be satisfied with the MU validation tools they were currently using, with 77% indicating that they would recommend their MU validation software to others. Most of those operating a TomoTherapy (Accuray, Sunnyvale, CA) unit did not perform an independent MU validation (79%). Respondents did report performing measurement‐based patient‐specific QA (text responses).

There were a variety of field verification surveys. For static field shape verification, the majority of respondents indicated that they take initial portal images of the treatment fields with the patient on the table to verify the MLC shape for each static field (90%) and take subsequent portal images of the treatment fields (69%). For patients being treated with conventional radiotherapy combined with daily IGRT, portal images of each field are acquired to verify all beams on the first day only (47%) or weekly (19%), to verify alternating fields weekly (11%), or are never acquired (17%).

Slightly less than half (47%) of respondents indicated that they perform routine *in vivo* dosimetry measurements for QA purposes. The main reason (38%) indicated for performing this dosimetry was that it is deemed to be valuable QA. Static photon and electron fields were identified as receiving the most routine *in vivo* dosimetry (62% and 55%, respectively) and a variety of devices were used to make these measurements: optically stimulated luminescent dosimeter (OSLD) (25%), diodes (24%), metal–oxide–semiconductor field‐effect transistor (MOSFET) (18%), and thermoluminescent dosimeter (TLD) (11%). For 2D and 3D treatments, 15% of respondents indicated that they use *in vivo* dosimetry most commonly to monitor implanted devices such as pacemakers. This number rose slightly to 19% when asked about electron treatments.

### 3.F IMRT and VMAT treatments

In IMRT planning, most centers (62%) do not use high energy photons (>10 MV), 13% use them for pelvis plans only, and 25% use them whenever it improved the plan. The majority (65%) of respondents do not modify pelvic IMRT/VMAT plans due to the presence of air cavities. For head and neck treatment plans, the majority (74%) of respondents do not modify plans due to the presence of air cavities such as the sinuses. Most (75%) contoured the high and low density artifact regions and reassigned the density as 1 g/cc for tissue and 0 g/cc for air when confronted with substantial artifacts due to dental metal. Metal artifact reduction capabilities in a scanner were reported as helping to reduce metal artifacts, but no frequency was given on its availability (text responses).

When adding a boost volume for brain and lung IMRT treatments, sequential boosts are used much more frequently (72% and 74%, respectively) than simultaneous integrated boosts. Four different surveys asked about the use of simultaneous integrated boost for head and neck IMRT cases. Two surveys asked how head‐and‐neck plans were boosted. Seventy‐four percent reported using simultaneous integrated boosts with the remainder using sequential boosts. Two other surveys asked if simultaneous integrated boosts were used for head‐and‐neck IMRT, with 80% of respondents reporting that they were. A majority (58%) of respondents who use sequential boosts plan the boost at the start of the treatment course. Although the boost is planned, 45% of respondents do not perform QA of the boost plan at the start of the first course of treatment.

A large majority of respondents (84%) do not image IMRT fields with patients on the table. Of the respondents who do image IMRT fields with patients on the table (16%), 40% film only a sample of the fields and 60% film all the fields. The rationale for not imaging IMRT fields with patients on the table was that IMRT fields are verified by 2D array (58%) and by portal imaging (42%).

For IMRT QA, when asked which method was the best available today, the top three choices from respondents were 3D‐detector‐based (51%), EPID‐based (20%), and 2D‐detector‐based (13%). A policy of completing patient specific QA before treating the first fraction was adopted by most respondents (90%). Portal dosimetry is used by over half the respondents (56%) for IMRT QA, and the majority of respondents (56%) do absolute dosimetry in addition. The majority of respondents (59%) report that the physicist performs these portal dosimetry measurements. A separate survey reports that sometimes (19%) the IMRT QA is shot by the therapist. Only 27% of respondents take *in vivo* dosimetry measurements for IMRT treatments. EPID transit dosimetry is the most widely used method (32%) for this. The majority of respondents (69%) indicated that they have changed plans based on IMRT/VMAT QA results. The majority of these changes occurred in highly modulated fields which prompted simplification of the plan (text responses). For forward planned field‐in‐field beams, the majority of respondents (77%) do not perform measurement‐based QA. Of those that do, it is typically (82%) performed by the physicist.

### 3.G SRS/SBRT

Most surveys inquiring about stereotactic treatments focused on cranial stereotactic radiosurgery (SRS) and lung SBRT. For cranial SRS, respondents reported a wide distribution of margins for creating the planning target volume (PTV) from the GTV: 0 mm (32%), 1 mm (39%), and 2 mm (30%). A variety of dose prescriptions were reported for tumors less than 20 mm in maximum diameter: 18 Gy (15%), 20 Gy (22%), 21 Gy (19%), 24 Gy (26%). If the patient had an MRI exam with contrast, about half (52%) of those surveyed said they would not request an additional contrast‐enhanced CT scan. For planning unilateral cranial SRS targets, 47% report frequently using beams which enter through the contralateral side, whereas 30% report infrequently using them. When asked about allowing beams to pass through critical structures, 33% frequently allow this provided the dose objectives are met, whereas 56% of respondents allow this only infrequently. Several text responses indicated that beams are not allowed to pass through the eyes.

For linac‐based SRS, cones were reported to be used by 41% of respondents for at least some cases. When MLCs are used to shape the fields, the reported MLC leaf width is either 5 mm (31%) or 2.5 mm (64%). For 3D plans (excluding IMRT or VMAT treatments), 47% of respondents are performing patient‐specific QA.

When asked generally about aligning SRS patients, the most common methods reported were using CBCT imaging (43%) or ExacTrac (Brain LAB AG, Feldkirchen, Germany) (31%). Others reported using orthogonal kV‐kV imaging (14%) or lasers (10%). Text responses suggested many respondents use a combination of methods, and that their methods depend on whether it is a frameless treatment or not. A majority (87%) of respondents reported that they perform cranial SRS using a frameless system. In a survey asking specifically about IGRT techniques for frameless SRS on a linac, respondents reported that they most often use CBCT (48%), followed by ExacTrac (42%) or kV‐kV imaging (10%). To verify alignment when couch angles other than 0 or 180 degrees are used, respondents reported that they most often relied on ExacTrac (41%), the couch isocentricity (33%), or surface guidance (21%). Seventy‐three percent of respondents performing cranial SRS reported having a six‐degrees‐of‐freedom couch.

For lung SBRT, the most common treatment technique was reported to be VMAT (55%). Given the option to purchase any breath control system for lung SBRT, the most popular methods were abdominal compression (48%), no control with gating (14%), or no control without gating (16%). In a survey about VMAT lung SBRT using flattening filter free (FFF) mode, 73% of respondents reported that their site uses FFF mode for VMAT lung SBRT. Of those that use FFF for SBRT, 19% reduce modulation during VMAT planning, 8% limit the dose rate, and 45% use abdominal compression. A majority of respondents (79%) do not perform a Winston Lutz test before each lung SBRT fraction.

There was a variety of responses about the required staff during stereotactic treatments. Thirty‐seven percent reported that physicians are required to be present at the linac for the entire first treatment, whereas 51% reported that physicians are only required to be present until setup is confirmed. In subsequent fractions, 21% reported that physicians are required to be present for the entire treatment, whereas 53% reported that physicians only had to be present until setup is confirmed.

In a separate survey, 72% reported that physicists are required to be present at the linac for the entire first stereotactic treatment. In subsequent fractions, 47% reported that physicists are required to be present for the entire treatment. For SRS treatments, 76% of responses said the medical physicist is required.

### 3.H Breast cancer treatments

For radiotherapy treatments of the breast, forward planned field‐in‐field was the most commonly reported technique among respondents for both right and left breast treatments (75% and 70%, respectively). Most respondents (77%) considered acceptable coverage of the PTV to be 95% of the volume covered by the prescription dose. Regarding hot spots, over half (56%) found 108% to be acceptable, whereas 110% and 105% were also acceptable hot spots to 23% and 19% of respondents, respectively. In regard to using high and low photon energies for field‐in‐field treatments, respondents were split almost equally among whether to use only low (31%), only high (38%), or both low‐ and high‐energy beams for modulation (31%). Eighty‐four percent of respondents reported using a sequential boost vs 16% who use a simultaneous integrated boost.

When asked what percentage of breast tangent patients are treated with inverse planned IMRT, 59% of respondents answered 0% of patients, whereas 31% of respondents answered between 1% and 24%. No respondents said that 100% of a patients are treated with IMRT, and only 8% said that more than 50% are treated with IMRT. According to respondents, the whole breast PTV contour necessary for IMRT are created either by the dosimetrist (65%) or by the radiation oncologist (35%). To create this structure, 54% said they follow an atlas, such as given by the Radiation Therapy Oncology Group (RTOG), whereas 46% apply tangents and create the PTV from an isodose line. When asked if using VMAT for breast or chest wall cases, 32% responded affirmatively. Some of the respondents commented that they only used VMAT for special or difficult cases. Of those that do, most (67%) generate flash around the external contour.

When specifically asked about blocking the humeral head during three‐field breast treatments, 63% reported that the radiation oncologist prefers to block the entire humeral head, whereas 25% only blocked 2/3 of the humeral head. Seventy‐one percent of respondents reported that the primary reason given by the radiation oncologist for blocking the humeral head was to prevent fibrosis which causes shoulder immobility.

In 2013, hypofractionation for breast treatments (266 cGy × 16 fractions) was not commonly used among respondents. Only 5% reported using this fractionation for at least 50% of patients. Twenty‐four percent reported never using this fractionation, and over half (56%) reported using it for between 1% and 25% of patients. For those that did use this fractionation, a variety of boosts were reportedly used, ranging from no boost (41%) to 10 Gy delivered in 4 or 5 fractions (24% and 31%, respectively). Most respondents (60%) reported primarily using electrons for breast boosts. There were a variety of policies regarding the percent of a treatment that can be delivered using high energy beams to reduce hot spots for patients treated with hypofractionation: never (13%), up to 25% (30%), up to 50% (37%), up to 75% (4%), or up to 100% (17%).

A majority (75%) of respondents reported using some type of motion management for breast radiotherapy. Of these, 88% reported using motion management for left breast only. A variety of techniques were reported, with the most common (46%) being Varian's built in systems, and the next most common (30%) is optical surface guidance. Many other options were reported and can be found in the Data S1. Fifty‐four percent of those using motion management reported using gating with a direct interface with the linac; another 21% reported having the therapist manually control the gating. Half of respondents (51%) use verbal feedback and 31% use both verbal and visual feedback. When asked what imaging protocol was used for breast tangents, a large majority reported using weekly MV ports only (82%).

About half of respondents (49%) reported that they treat some breast patients with a prone technique. For those that do, a majority of respondents (64%) reported that at least two‐thirds of patients with pendulous breasts, who are candidates for prone treatment, are treated using this technique. Twenty‐five percent reported that less than one‐third of eligible patients are treated with a prone technique.

The most common techniques reported for using bolus for postmastectomy chest wall treatments is 0.5 cm bolus either every other day (54%) or every day (21%). If the skin reaction becomes too severe, the most common responses were to stop using bolus immediately (48%) or to give a treatment break (33%). When presented with the specific scenario of discontinuing the daily use of bolus after 25 of 28 fractions, about half (53%) of respondents said they would require a replan. Furthermore, 45% responded that they would require the physician to write a whole new prescription.

### 3.I Prostate cancer treatment

In external beam radiation therapy for prostate cancer, a survey showed that most centers follow the RTOG 0924 simulation instructions: no urethral contrast (64%); no rectal contrast (79%); reproducible bladder fullness (86%); and rectum as empty as possible (71%). The only exception is that less than one‐third of respondents give the patient an enema before simulation (29%). A different survey asked about specific uses of contrast for prostate simulation: 21% use IV contrast, 26% use urethral‐administered contrast, 9% use a Foley Catheter with contrast, 12% use rectal contrast, and 12% use small bowel contrast. In three surveys conducted in 2013, rectal balloons were reported to be used by only a small number of respondents (15%). Eight percent of respondents are using gel spacers, although a few respondents commented that they use gel spacers in special cases or plan to implement them soon.

In treatment planning, 71% of the respondents indicated that they do not use simultaneous integrated boosts when treating prostate plus pelvic lymph nodes, whereas sequential boosts are used for prostate IMRT by 78% of respondents. Traditional fractionation (38–43 fractions) is much more commonly used than hypofractionation (20–27 fractions) or prostate SBRT (3–7 fractions). Seventy‐nine percent of respondents reported that at least half of their patients are treated with traditional fractionation, whereas only 13% and 3% reported that at least half of patients are treated with hypofractionation and SBRT, respectively.

The most common techniques for daily IGRT of the prostate are CBCT without fiducial markers (41%) and kV‐kV imaging with fiducial markers (31%). Of the respondents who have kV CBCT capabilities, 54% indicated that they use CBCT without fiducial markers, 17% use CBCT with fiducial markers, and 29% use an orthogonal kV‐kV pair with fiducials for alignment. If substantial rectal filling happens on a given day, about half (48%) of respondents indicated that they go ahead and treat the patient, whereas the rest (52%) will have the patient evacuate before treatment. If a patient has consistent rectal filling, 65% of respondents indicated that they will rescan and replan for the treatment.

### 3.J Brachytherapy

One survey showed that 23% of respondents continue to utilize low‐dose rate (LDR) Cs‐137 for gynecological brachytherapy, whereas the rest use high‐dose rate (HDR). Most (76%) reported that their institution still use both Cs‐137 and have an HDR program. From those that no longer use Cs‐137, most (63%) had discontinued the use of Cs‐137 more than 5 yr ago; from those still using Cs‐137, 23% expect to discontinue its use within 5 yr. Another survey specific to cervical cancer brachytherapy showed that the vast majority (97%) use HDR Ir‐192, and the rest use LDR Cs‐137.

There were two main approaches to entering the HDR source activity into the treatment planning system: using the manufacturer's certificate value (70%) or using the clinic's measured value (28%). Interest in purchasing software capable of heterogeneity corrections is roughly evenly split (54% in favor). For those, not in favor, the reasons include the cost of the software and a wish to be consistent with historical data (text responses).

When asked about where they perform HDR brachytherapy for cervical cancer, 59% of respondents use the operating room for each fraction, and 41% use it for just the first fraction. Text responses indicated that there are many other options, including the use of dedicated HDR suites, the department's CT simulation room, and a regular treatment vault. Most hospitals use gauze for packing (87%), although a specialized packing balloon is also used (13%). Applicator positioning is usually verified using CT (89%), although orthogonal x‐ray imaging and MRI are also used (8% and 3%, respectively). Text responses indicated many combinations of these are in use, especially MRI fused to CT. The dose is prescribed to either point A (57%) or using volume‐based optimization (44%); text responses indicated that it is common to do both, with point A often used for historical recording.

Regarding brachytherapy seeds, 70% of respondents assay seeds when they receive an order from the vendor. The number of assayed seeds varies widely among respondents: 5, 10, or 15 seeds, 10% of seeds, all seeds, etc. (text responses). In most cases (74%), additional seeds are ordered from the same batch specifically for this purpose.

### 3.K Total body irradiation (TBI)

In one 2016 survey, 80% of respondents’ clinics offer TBI, 11% do not, and the rest either used to, or intend to. Almost half of the respondents (47%) use an anteroposterior–posteroanterior beam arrangement, 37% use laterals, and the rest use a combination of these, additional fields, or TomoTherapy. Other techniques (such as 3‐field VMAT) were also mentioned in the text responses. Reported treatment positions include lying down (66%), standing (21%), and seated (15%). The use of compensators is wide‐spread (82%), with a variety of compensators reported, including lung and head compensators (text responses). The majority (73%) of TBI dose calculations are done manually, with the physicist nearly always (90%) involved in the calculations and additional involvement by dosimetrists (23%). The use of *in vivo* dosimetry is widespread (84%) at initial setup, with initial setup performed by therapists (85%) and physicists (79%). Several radiation detectors are used, including the following: diodes, MOSFETs, TLDs, OSLDs and ion chambers (text responses). Physicists are also often present for subsequent fractions (60%).

### 3.L Facial lesion treatments

Facial lesions are treated with either electrons (80%) or superficial x‐rays (20%). When using electrons, 46% use skin blocking and the rest use a cutout in the electron cone. When using skin blocking, 55% use a customized lead mask.

### 3.M Clinical workflow

Surveys pertaining to workflow were split into three groups: efficiency, task assignment, and workload. Topics in efficiency address turnaround time for treatment plan creation. Task assignment discusses the duties and roles of the therapist, dosimetrist, and physicist. The workload section pertains to the number of patients treated and time per treatment.

#### 3.M.1 Efficiency

The expected turnaround time for a physician to contour a CT dataset for a 3D plan was reported as within 1 day (47%), 2 days (42%), or 3 days (11%). The turnaround time for a dosimetrist to develop a 3D plan for the physician to review was reported as within 1 day (31%), 2 days (42%), or 3 days (27%). For IMRT, the average turnaround time was slightly longer for both physician contouring and dosimetrist planning. The expected turnaround time for a physician to contour the CT dataset for an IMRT plan was reported as within 1 day (16%), 2 days (55%), 3 days (21%), or 4 days (7%). The expected turnaround time for a dosimetrist to generate an IMRT plan for the physician to review was reported as within 1 day (0%), 2 days (30%), 3 days (34%), or 4 days (36%).

For the above‐mentioned tasks, respondents were further questioned on what factors expedite or prolong these processes. Some of the factors that many respondents reported as helping to expedite contouring were physician availability, physician willingness/urgency, having outside fusion data be available at the time of contouring, and having dosimetrists perform most of the contouring while the physician verifies. For expediting planning, many respondents reported that this hinges on physician's ability to provide contours in a timely fashion. Others mentioned that communications between the physician and dosimetrist were key and that clear instructions and directives helped to expedite planning.

#### 3.M.2 Task assignment

The most common tasks that it was reported a therapist performs are the following: perform CT simulation (95%), make custom bolus (75%), make electron blocks (72%), daily/weekly CT simulation QA (79%), daily linac QA (94%), daily IGRT QA (85%), create treatment and imaging calendar in record and verify system (59%), record SSDs (73%), report changes in patient separation (77%), and initiating the HDR treatment at the console (43%).

The most common tasks that it was reported a dosimetrist performs are the following: image fusion (93%), normal anatomy contouring (91%), autosegmentation (67%), creating PTVs from physician‐drawn GTVs (67%), IMRT planning (95%), SBRT planning (79%), additional protocol‐related work (73%), pushing the plan for secondary MU calculation (93%), and emergent after‐hours planning (81%).

The most common tasks that it was reported a physicist performs are the following: SRS planning (56%), HDR planning (73%), prostate seed implant planning (68%), taking measurements at HDR simulation (59%), reviewing plans after physician approval (95%), approving MU validation (86%), and weekly chart checks (95%).

**Figure 2:**
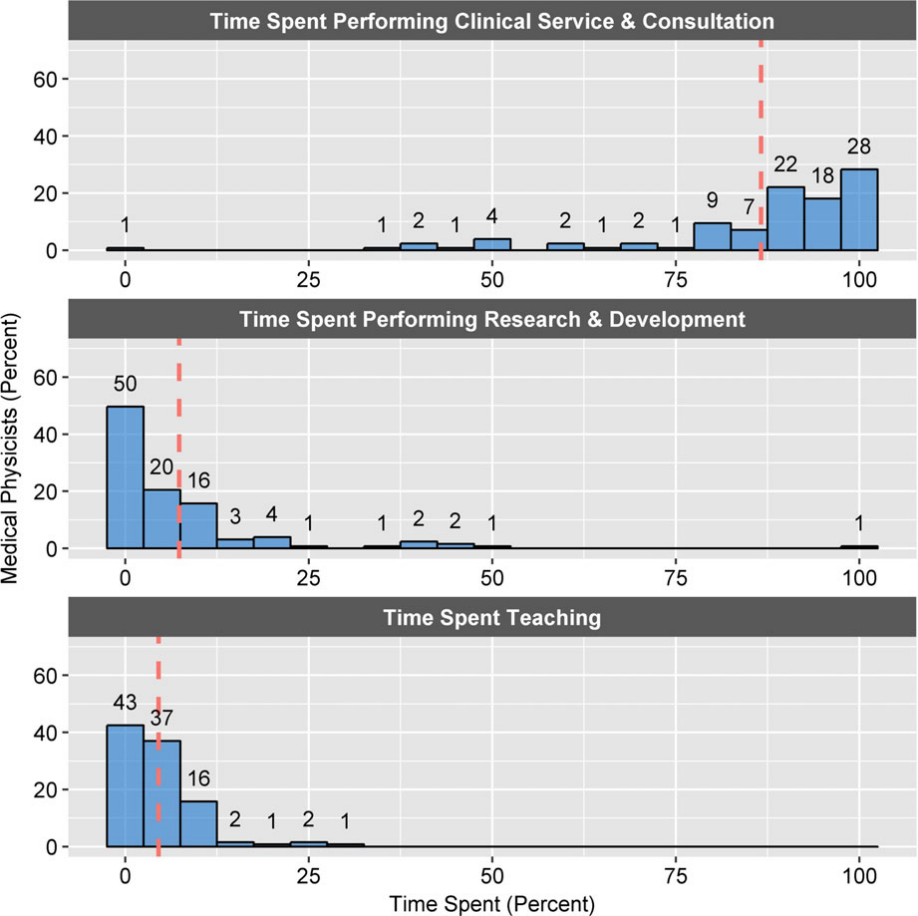
Histogram distributions of work time spent by medical physicists. Total number of respondents is 127. On average respondents spend 87%, 7%, and 5% of their time performing clinical service and consultation, research and development, and teaching, respectively. The width of each bin is 5%. The dashed red line indicates the average percent of work time spent for each category.

An additional survey pertaining specifically to medical physicists assessed the amount of time that medical physicists spend performing different activities. Although the data were collected in a free response format, it was possible to generate histogram distributions since all responses were reported numerically (Fig. 2). Most physicists spend a majority of their time performing clinical and consultation duties, with 28% of respondents dedicating over 95% of their time to clinical service and consultation. On average respondents spend 87% of their time performing clinical service and consultation, 7% of their time performing research and development, and 5% of their time teaching. In a separate survey, 22% of respondents indicated that physicists are included on the medical staff at their facility.

In a 2017 survey involving the duties of medical physics assistants, 24% of respondents indicated that their institution employed medical physics assistants, with most of them performing QA tasks and IMRT/patient specific QA tasks.

#### 3.M.3 Workload

In a 2013 survey, 54% of respondents reported a decline in patient numbers at their centers in the past 5 yr with 16% experiencing more than a 30% decline. Twenty‐eight percent of respondents reported minimal change in patient numbers (within 10%) in the past 5 yr. Respondents identified the changes to be caused by competition from nearby radiation oncology centers (72%), the economy (52%), lack of patient insurance (30%), competing surgical therapies (25%), competing chemotherapy regimens (18%), changes in screening guidelines (15%), etc. When asking about future patient numbers, about half of respondents (49%) anticipated minimal change (less than 10%) in next 5 yr, whereas 19% anticipated an increase of 10‐20%, and 18% anticipated a decrease of 10‐20%.

Most respondents (80%) indicated that they schedule 15‐min linac treatment time slots. For a routine new patient start, respondents scheduled 15 min (16%), 30 min (75%), or more than 30 min (8%). Respondents reported routinely treating less than 20 patients per day per linac (8%), 20 patients (15%), 25 patients (40%), 30 patients (21%), 35 patients (9%), and 40 patients or more (8%).

### 3.N After‐hours/emergent treatments

Of the respondents, 91% indicated that they provide radiotherapy for emergent patients after‐hours. For those who do not treat after‐hours, 80% will treat the patients at the start of the next clinical day; others send patients to another facility which does treat emergent patients. For those who do treat after‐hours, 85% are community hospitals or free‐standing cancer centers and 15% are academic institutions. Eighty‐one percent of centers treated 20 or less emergent cases in a year. Respondents reported the following cases to be emergent: cord compression (100%), superior vena cava syndrome (92%), whole brain (53%), excruciating bone metastases (53%), and rectal bleeding (35%).

Most respondents (96%) consider physicians and therapists as essential on‐call personnel who are required to attend the emergent treatment, 33% of respondents consider dosimetrists and 14% consider physicists as essential on‐call personnel. In preparing for an emergent treatment, 56% of respondents indicated that they use manual calculations and 38% indicated that they perform CT simulation and computer planning. If the patient receives a CT simulation, 81% reported that the dosimetrists were responsible for the planning, whereas 31% reported that the physicists do the planning. Approximately half of respondents (52%) reported that the treatment fields are entered into the record and verify before treatment, whereas others capture the treatment parameters on the table (29%) or treat in standby mode (19%).

## 4 DISCUSSION AND CONCLUSION

This work summarizes responses to 139 surveys distributed to the MEDPHYS and MEDDOS listservs over a 5‐yr period, covering topics of particular interest to practicing medical physicists. These were informal surveys created in response to questions posed by the listserv membership. The surveys were conducted anonymously, which helps increase the number of responses and also means people are often more comfortable giving honest responses. Each survey provides a snapshot of practice patterns relevant to the medical physicist. Until now, the medical physics community has lacked a summary of this information, and it is not always easy for individual physicists to access information about how the wider community is practicing. In addition to summarizing the surveys, the quantitative results are now reproduced in a single document (the Data S1).

There are a few limitations to this work. The initial intent of these surveys was to informally collect responses from the medical physics community and not to provide data for extensive scientific analysis. As such, the reader should bear in mind that the survey participation and responses to individual questions may be influenced by many factors, including the context of the survey and construction of the question and answer choices. For example, the multiple choice options were not necessarily an exhaustive list, but often did include an “other” option. Additionally, when combining results from similar surveys, we did not have a means to determine if one person responded more than once, therefore we made the assumption that all respondents were unique. Although guidelines for survey design exist, due to the informal nature of these surveys, these guidelines were not generally followed. As a result, the respondents’ demographics remain unknown, and the survey participants were not randomly selected from the population. Due to these limitations, we were unable to estimate or control for representation bias.

Despite these limitations, we feel that this summary is instructive and provides information that is not available elsewhere. Furthermore, we hope that these informal studies generate a preliminary idea of medical physics practice, and that this can lead to the design of more rigorous surveys of practice patterns in the future.

In summary, we have provided a coherent overview of medical physics practice according to surveys conducted over the last 5 yr, which will be useful to medical physicists.

## CONFLICTS OF INTEREST

No conflicts of interest.

## Supporting information


**Data S1.** Tabulated responses to all multiple choice surveys analyzed.
