# Supplementary material for: A snapshot of medical physics practice patterns
Source: J Appl Clin Med Phys. 2018 Oct 1;19(6):306–15. doi: 10.1002/acm2.12464 (PMC6236839; doi:10.1002/acm2.12464)
Supplement: Supplementary file 1 — Data S1. Tabulated responses to all multiple choice surveys analyzed. [file ACM2-19-306-s001.pdf]

SUPPLEMENTAL MATERIAL

The results of 139 multiple choice surveys used for analysis are provided below with our calculation of the uncertainty reported, as described in the manuscript. Individual surveys had a variable number of questions. Additionally there were three types of multiple choice questions which affected how the uncertainty was calculated.

1.) Only one response allowed. In this type, the uncertainty was calculated using the number of responses for all answer choices.
2.) One response allowed with a comment option, which was often labeled as “Other (please specify).” In this type, the uncertainty was calculated using the number of responses for all answer choices except for the comment option.
3.) Select all that apply. In this type, the uncertainty was calculated separately for each answer choice as a binomial of the number of responses for that answer choice relative to the total number of responses for the question, excluding the comment option.

TABLE OF CONTENTS

Survey Name	Creation date	Questions
3D Scanner or 1D Scanner plus 2D Array?	4/12/2017	If you were starting a new program, which would you purchase for linac commissioning and annual QA?
3D Scanning System	5/3/2016	If you were to purchase a 3D Scanning System today, which would you choose?
6DOF Couch Top for Cranial SRS	5/18/2017	For those of you who perform Cranial SRS, do you have a 6DoF Couch Top?
Adaptive Radiotherapy	4/2/2014	How often do your replan Head/Neck IMRT cases after they start?<br>What are your criteria for replanning? (check all that apply)<br>If you do not replan, what are the reasons? (check all that reply)
After Hours Radiotherapy	7/3/2015	Does your facility provide radiotherapy for emergent patients after hours?<br>If no, how do you manage these patients? (Then skip to Q13)<br>If yes, what is your type of facility?<br>How many emergent cases per year are treated?<br>Which cases does your facility consider emergent? (check all the apply)<br>Which personnel are on-call and required to attend the emergent treatment? (check all that apply)<br>How is the patient prepared for treatment?<br>If CT Simulated / Computer Planned, who does the planning?<br>How is the R&V component managed?<br>How is the imaging managed?<br>What is the availability during the week?<br>What is the availability on Saturday, Sunday, and Holidays?
APBI Balloon Verification	8/21/2013	How do you verify the APBI balloon volume and position before each fraction?
Beam Energy for IMRT	3/11/2013	How often do you use high energy photons (> 10 MV) for IMRT?
Beam Naming Convention	10/2/2017	What convention do you use for beam naming?

Beam Naming Convention - medphys	10/2/2017	What convention do you use for beam naming?
Beam Profiles	5/25/2015	Which cross-beam profiles do you have in your planning system?<br><br>With regards to TG142 baseline symmetry, what is your baseline?<br><br>When quoting symmetry, which evaluation are you using?
Brachytherapy for Cervix Cancer	11/25/2015	If you perform brachytherapy for cervix cancer, are you in a hospital or free standing clinic?<br><br>If you perform brachytherapy for cervix cancer, what modality do you use?<br><br>For those who use HDR, do you bring the patient to the OR for each fraction or just the first (assuming a Smit Sleeve is used?<br><br>Do you find the use of brachytherapy for cervical cancer to be increasing, staying the same, or decreasing?<br><br>What do you use for packing?<br><br>What imaging method do you use?<br><br>What prescription method do you use?
Brass Mesh Bolus with 15x or 18x	7/31/2014	Do you use brass mesh bolus for post-mastectomy chestwalls?<br><br>If yes, do you use it when one or more of the beams are 15X or 18X?
Breast Flash with VMAT	9/13/2016	Do you use VMAT to treat Breast or Chestwall cases?<br><br>If Yes, do you somehow generate Flash around the external contuour?
Breast Planning Questions	7/28/2016	What technique do you typically use when planning Right Breast Tangents?<br><br>What technique do you typically use when planning Left Breast Tangents?<br><br>What is the acceptable Hot Spot?<br><br>What is the acceptable Dose Coverage of the PTV?
Breast Planning Techniques	7/14/2014	What technique do you typically use when planning Right Breast Tangents?<br><br>What technique do you typically use when planning Left Breast Tangents?

		What percentage of your breast tangent patients are treated with Inverse Planned IMRT technique?
Breast Radiotherapy	2/22/2013	Let us assume the "Canadian Protocol" for breast radiotherapy is 266 cGy x 16. How often do you use that fractionation scheme for your patients?<br><br>How do you typically boost these patients?<br><br>What is your policy on using higher energy photons (15 MV, 18 MV) to reduce the hot spots?<br><br>What modality to you use for breast boosts?
Checking SSDs - meddos	2/2/2017	Do your therapists check and record the SSDs on the first fraction for non-IMRT fields?<br><br>Do your therapists check and record the SSDs on a weekly basis for non-IMRT fields?<br><br>Do you do the same for IMRT patients?<br><br>Do you do the same for IGRT patients?
Checking SSDs - medphysusa	2/2/2017	Do your therapists check and record the SSDs on the first fraction for non-IMRT fields?<br><br>Do your therapists check and record the SSDs on a weekly basis for non-IMRT fields?<br><br>Do you do the same for IMRT patients?<br><br>Do you do the same for IGRT patients?
Chestwall Bolus Technique	12/12/2013	What technique do you use for bolusing postmastectomy chestwalls?<br><br>What happens if the skin reaction gets too severe?
CMD Contouring	2/6/2013	Do you contour the prostate for prostate cases?<br><br>Do you contour the bladder for prostate cases?<br><br>Do you contour the rectum for prostate cases?<br><br>Do you contour the parotids for head/neck cases?<br><br>Do you contour the healthy lung for mediastinal cases?<br><br>Do you contour the spinal cord for all cases?<br><br>Do you contour the optic chiasm for brain cases?
CMD Contouring - 2016	9/16/2016	At your center, who contours the normal anatomy and Organs At Risk?<br><br>At your center, who contours the nodal PTVs?

		At your center, who creates the tumor PTV after the physician has drawn the GTV?
		At your center, who creates the ITV for lung cases?
		At your center, who performs the Atlas Based Auto Segmentation?
Coaching for DIBH Patients	7/20/2017	If you provide coaching for DIBH treatments, what method do you use most often?
Constancy Check of Electrometer-Chamber System	10/19/2015	How do you perform a constancy check of your electrometer-chamber combination?
		How often do you perform a constancy check of your electrometer-chamber combination?
Contrast for Prostate only CT Sim	5/13/2013	For Prostate CT Sim, do you use IV contrast?
		For Prostate CT Sim, do you use urethral administered contrast?
		For Prostate CT SIm, do you use a Foley Catheter with contrast?
		For Prostate CT Sim, do you use rectal contrast?
		For Prostate CT Sim, do you use small bowel contrast?
		For Prostate CT Sim, do you use a rectal balloon?
Copy of SIB for Prostate and PLN- meddos	12/16/2015	Do you use Simultaneous Integrated Boost when treating prostate plus pelvic lymph nodes?
Cranial SRS Planning	10/7/2014	When treating a unilateral cranial SRS target, do you use beams/arcs which enter through the contralateral side?
		Do you allow beams to pass through a critical structure provided the dose objectives are met?
Creating Lung ITV with 4DCT-meddos	6/26/2015	How do you create your Internal Target Volume (ITV) for lung patients scanned with 4DCT?
Cs-137 for GYN Brachytherapy	11/3/2016	Does your hospital continue to utilize Cs-137 for GYN brachytherapy treatments?
		If no, when did you discontinue its use?
		If yes, when do you plan to discontinue using Cs-137?
		If yes, do you run an HDR program in addition?
		Do you solely rely on an HDR unit now for all GYN brachytherapy applications?

Dental Metal Artifacts	6/8/2016	How do you manage Head/Neck patients who have substantial artifacts due to dental metal when running an IMRT/VMAT plan?
Do you assay seeds?	12/20/2013	Do you assay any seeds when you receive an order from the vendor?<br><br>If you do assay seeds, how many do you assay?<br><br>If you assay seeds, how to you acquire the seeds you assay?
Dose Specification - Water or Medium?	6/23/2016	For photon plans, do report ''dose to water'' or ''dose to medium''?<br><br>For electron plans, do you report "dose to water" or "dose to medium"?
Electron Calculations	11/30/2014	How do you account for the Electron Cutout Factor used for MU calculations?<br><br>If you measure individual Electron Cutout Factors, what is your method?<br><br>Do you treat with the MU determined by a manual calculation or by the treatment planning computer?<br><br>What is the maximum SSD which you would use calculated MU without phantom validation?
Electron Cutout Factors	4/13/2013	How do you handle the MU Calculation when using an Electron Cutout?
Electron MUs and Isodose Plans	4/20/2013	How do you determine the MU to be used for electron treatments?<br><br>What type of Isodose Plan do you generate for the chart?<br><br>How is the Isodose Plan presented?
Electron MUs and Isodose Plans - 2016	2/19/2016	How do you determine the MU to be used for electron treatments?<br><br>What type of Isodose Plan do you generate for the chart?<br><br>How is the Isodose Plan presented?
FFF beams for Breast Tangents	7/25/2014	For those of you with TrueBeam, do you use FFF mode for breast tangents to reduce the treatment time for DIBH techniques?
Fiducial Markers		Do you use fiducial markers for IGRT?<br><br>If yes, what type of fiducial marker do you use?<br><br>If yes, what design of fiducial marker do you use?

		If yes, what vendor supplies your fiducial markers?
Filming IMRT Fields	3/20/2014	Do you film IMRT fields with the patient on the table?<br><br>If yes, do you film all the fields or only a sample?<br><br>If you do not film the IMRT fields, what is your rationale?
Frame vs Frameless SRS	5/27/2015	If you perform Cranial SRS, do use a Frame (pins into skull) or Frameless (no pins into skull) system?
Frame vs Frameless SRS-medphys	5/29/2015	If you perform Cranial SRS, do use a Frame (pins into skull) or Frameless (no pins into skull) system?
Gafchromic Film	6/11/2015	What type of Gafchromic film do you use for Light/Radiation QA?<br><br>What type of Gafchromic film do you use for Picket Fence QA?<br><br>What type of Gafchromic film do you use for IMRT QA?<br><br>What type of Gafchromic Film do you use for SRS QA?<br><br>Do you analyze in relative or absolute dose?
Gel Phantoms	4/1/2016	Would you be interested in using a Gel Phantom for dosimetry validation?<br><br>If yes, what applications would you be testing? (check all that apply)<br><br>If yes, how would you use such a phantom? (check all the apply)<br><br>If yes, which type of solution would you prefer?<br><br>If no, why not?
Generating Composite Dose Distributions	2/26/2016	When your clinic encounters a patient with prior radiation does, is a composite isodose distribution generated?<br><br>If your clinic generates composite isodose distributions, how is it accomplished?<br><br>In general which method of generating a composite isodose distribution is most accurate?<br><br>If your Clinic generates composite isodose distributions, who is responsible for the work?<br><br>Do you bill a Special Physics Consult 77370 for composite isodose distributions?<br><br>If using DIR software do you bill a Special Physics Consult 77370 for deformable image registration?

GTV-PTV for SRS	6/4/2015	For Cranial SRS, what margin around the GTV do you use for creating the PTV?
Hard Wedges- meddos	5/13/2015	Do you still use Hard Wedges for any patient treatments?
Hard Wedges- medphys	5/13/2015	Do you still use Hard Wedges for any patient treatments?
HDR Source Activity	6/17/2016	What HDR Source Activity do you enter into the treatment planning system and control console?
Higher Photon Energy	9/2/2013	If you could alter the energy pair on all your linacs to one standard, what would you choose?
Humeral Head blocking	3/20/2015	When treating three field breasts, how does your radiation oncologist block the humeral head?<br>What does your radiation oncologist say is the primary reason to block the humeral head?
Hybrid IMRT and QA	8/11/2016	Given the stated definition of Hybrid IMRT, would you perform QA for these plans?
Hypofractionation for Prostate	9/11/2015	Does you center use standard fractionation such as 38-43 fractions for prostates?<br>If no, how many fractions do you use?
IGRT for Breast Tangents	1/27/2014	Which imaging protocol do you follow for breast tangents?
IGRT for Frameless SRS	6/2/2015	If you perform frameless SRS on a linac, what IGRT technique do you use?
IGRT Shift Limits	7/1/2014	What is the maximum shift your therapists allowed to make independently?<br>If there is a shift limit, who must be called to approve it?<br>Have you ever had a treatment event because an inappropriate shift was applied?
Implementation of 10X FFF	4/27/2017	For those of you with 10X FFF, have you commissioned it?<br>If yes, do you find it is sometimes superior to 6X FFF?
Implementation of 10X FFF - meddos	4/24/2017	For those of you with 10X FFF, have you commissioned it?<br>If yes, do you find it is sometimes superior to 6X FFF?
IMRT Boost Techniques - meddos	2/22/2017	How do you boost Prostate cases?<br>How do your boost Head and Neck cases?<br>How do you boost Brain cases?

		How do you boost Lung cases?<br><br>How do you boost Breast cases?<br><br>If you use Sequential Boosts, do you run the boost plan at the start or later in the course?<br><br>If you use Sequential Boosts, do you QA the boost plan at the start or later in the course?
IMRT Boost Techniques - medphys	2/21/2017	How do you boost Prostate cases?<br><br>How do your boost Head and Neck cases?<br><br>How do you boost Brain cases?<br><br>How do you boost Lung cases?<br><br>How do you boost Breast cases?<br><br>If you use Sequential Boosts, do you run the boost plan at the start or later in the course?<br><br>If you use Sequential Boosts, do you QA the boost plan at the start or later in the course?
IMRT/VMAT planning and Air Cavities	7/12/2017	For Head-Neck IMRT/VMAT planning, how do you handle air cavities such as sinuses, oral cavity, etc?<br><br>For Pelvis IMRT/VMAT planning, how do you handle air cavities due to flatulence?
IMRT/VMAT planning and Air Cavities - meddos	7/12/2017	For Head-Neck IMRT/VMAT planning, how do you handle air cavities such as sinuses, oral cavity, etc?<br><br>For Pelvis IMRT/VMAT planning, how do you handle air cavities due to flatulence?
IMRT/VMAT QA - Why?	1/2/2018	Have you ever changed a plan based on the IMRT/VMAT QA?
IMRT/VMAT QA Methods	4/3/2017	Which method do you feel is the best available today for IMRT/VMAT QA?
In Vivo Dosimetry	8/20/2017	Do you perform ROUTINE in vivo dosimetry measurements (i.e. on patient during actual treatment) for QA purposes?<br><br>If yes, for which of the following treatment types do you use ROUTINE in vivo dosimetry for QA? (click all that apply)<br><br>If yes, what is the MAIN reason you perform ROUTINE in vivo dosimetry?<br><br>If yes, do you bill for this service?
Invivo Dosimetry	1/6/2016	What do you use for invivo dosimetry?

IV contrast for CT Sim	3/4/2014	When do you use IV contrast for CT Simulation? (Select all that apply)<br><br>When contrast is used, how do you then plan?
IVD for 2D and 3D Routine Treatment Delivery QA	10/18/2017	For 2D and 3D Photon treatments, do you make routine vivo dosimetry measurements of the delivered dose to the patient?<br><br>For Electron treatments, do you make routine vivo dosimetry measurements of the delivered dose to the patient?
IVD for Treatment Delivery QA	11/21/2016	For IMRT/VMAT treatments, do you make vivo dosimetry measurements of the delivered dose to the patient?<br><br>If yes, what type of vivo measurements do you make?
Linac Output Reference Point	7/7/2014	What is the geometry which defines the output reference point calibrated to be 1 cGy/MU at your facility?
Linac SRS Questions	9/21/2017	How do you verify the patient alignment when using tables angles other than 0/180?<br><br>What is the leaf width of your MLC?<br><br>Do you ever use cones for SRS?
Lung SBRT Options	12/29/2015	Which immobilization system would you purchase today for your Lung SBRT program?<br><br>Which breath control system would you purchase for your Lung SBRT program?<br><br>Which treatment technique would you use for Lung SBRT?
Lymph Node Bed PTV Contouring	2/10/2013	Do you contour the Head/Neck Lymph Node Beds which the radiation oncologist then reviews and revises as needed?<br><br>Do you contour Pelvic Lymph Node Beds which the radiation oncologist then reviews and revises if needed?<br><br>Do you contour the Bowel Bag which the radiation oncologist then reviews and revises as needed?<br><br>Do you contour the Lumpectomy Bed which the radiation oncologist then reviews and revises as needed?
Mixed Energy Breast Tangents	3/19/2013	Suppose you are using mixed energy tangents (6X and 15X) and Forward Planned Field-In-Field beams to plan a large breast. Which beams would you modulate? Please explain why is the Other field.
Monthly Linac QA Methods	8/17/2017	How do you verify the linac output when performing monthly QA?

		How do you verify energy on a monthly basis?<br><br>How do you verify flatness and symmetry on a monthly basis?
More questions regarding Lung 4DCT- revised	8/3/2015	How do you create your Lung ITV?<br><br>Once you have created your ITV, how do you manage its Density?<br><br>How do you treat the PTV expansion?<br><br>How do you gate during Lung SBRT treatments?<br><br>What treatment modality do you primarily use for Lung SBRT?
Motion Management for Breast Radiotherapy	6/2/2017	Do you use any type of motion management for breast radiotherapy?<br><br>If yes, what technique do you use?<br><br>If yes, what Gating method do you use?<br><br>If yes, what feedback method do you use?<br><br>If yes, what patients are treated with motion management?
Motion Management for Breast Radiotherapy - meddos	5/29/2017	Do you use any type of motion management for breast radiotherapy?<br><br>If yes, what technique do you use?<br><br>If yes, what Gating method do you use?<br><br>If yes, what feedback method do you use?<br><br>If yes, what patients are treated with motion management?
MU validation	2/7/2013	What MU Validation software do you use?<br><br>Would you recommend others get what you use?
Multimodality Composite Plans	3/2/2016	Have you ever generated a composite plan with XRT plus SRS? Please explain your answer in the Comment field.<br><br>Have you ever generated a composite plan with XRT and SBRT? Please explain your answer in the Comment field.<br><br>Have you ever generated a composite plan with XRT and HDR? Please explain your answer in Comment field.<br><br>Have you ever generated a composite plan with XRT and LDR? Please explain your answer in the Comment field.

		Have you ever generated a composite plan with HDR and LDR? Please explain your answer in the Comment field.
Patient Specific QA for SRS	4/21/2016	Do you perform patient specific QA for linac based SRS cases using 3D plans? (This includes plans using static or rotational conformal beams. It excludes plans using IMRT or VMAT beams.)
Patient Specific VMAT QA	8/14/2013	Based on your experience and/or investigation, which system would your purchase today for Patient Specific VMAT QA?
Patient Specific VMAT QA Policy	8/14/2013	What is you policy for Patient Specific VMAT QA?
Patients per linac	10/14/2015	How much time do you schedule for routine patient treatments?<br>How much time do you schedule for routine new patient starts?<br>How many routine patients per day do you treat on one linac?
Perturbation Factors for SRS	10/14/2014	Are you aware of the recently recommended Perturbation Factors pertaining to SRS dosimetry measurements?<br>Do you plan to apply these Perturbation Factors to your measured SRS data?
Physician Presence at SRT	1/15/2016	What does your facility require of the physician for the first SRT treatment?<br>What does your facility require of the physician for the subsequent SRT treatments?<br>Does your facility believe it is a billing requirement for the physician to personally attend each SRT treatment?
Physician Supervision	2/9/2015	In your operation, is the physician required to make a personal appearance at each and every simulation?
Physicist Presence at SRS/SRT	1/11/2016	What does your facility require of the medical physicist for a SRS treatment?<br>What does your facility require of the medical physicist for the first SRT treatment?<br>What does your facility require of the medical physicist for the subsequent SRT treatments?<br>Does your facility believe it is a billing requirement for a physicist to personally attend a SRS/SRT treatment?
Physicists as Medical Staff	10/10/2017	Are Physicists included on the Medical Staff at your facility?

Portal Dosimetry for IMRT/VMAT QA - medphysusa	12/8/2015	Do you use portal dosimetry for IMRT / VMAT QA?<br><br>Whether or not you use portal dosimetry, is an absolute dose measurement necessary?<br><br>Whether or not you use portal dosimetry, is it appropriate to bill for IMRT (77301) if the QA is done with portal dosimetry?<br><br>If you use portal dosimetry, do you generate an absolute dose measurement?<br><br>If you use portal dosimetry, do you bill for IMRT (CPT 77301) without any other measurement?<br><br>If you use portal dosimetry, who takes the acquires the measurement?
Portal Imaging and IGRT	11/2/2016	For patients who get daily KV or CBCT IGRT, what is your policy regarding MV portal imaging for non-IMRT beams?
Pregnant Physicists and Brachytherapy	8/17/2015	What is your policy regarding pregnant physicists and prostate seed implant procedures?<br><br>What is you policy regarding pregnant physicists and HDR procedures?
Prone Breast Teletherapy	6/12/2014	Do you treat some of your breast patients with a prone technique?<br><br>If yes, which system do you use?<br><br>If yes, which system would you prefer based on your experience?<br><br>If yes, what percentage of pendulous breast patients who are candidates for a prone technique actually get treated with a prone breast technique?
Prone Breast Teletherapy - 2015	8/4/2015	Do you treat some of your breast patients with a prone technique?<br><br>If yes, which system do you use?<br><br>If yes, which system would you prefer based on your experience?<br><br>If yes, what percentage of pendulous breast patients who are candidates for a prone technique actually get treated with a prone breast technique?
Prostate IGRT with CBCT	12/23/2014	For those of you with KV CBCT capability, how do you perform IGRT for the prostate?

Prostate Teletherapy Fractionation	6/29/2017	What percentage of your prostate patients are treated with traditional fractionation (38-43 fractions)?<br><br>What percentage of your prostate patients are treated with hypofractionation (20 – 27 fractions)?<br><br>What percentage of your prostate patients are treated with SBRT (3 - 7 fractions)?<br><br>Does your center use gel spacers for prostate teletherapy?
Prostate Teletherapy Fractionation - meddos	6/29/2017	What percentage of your prostate patients are treated with traditional fractionation (38-43 fractions)?<br><br>What percentage of your prostate patients are treated with hypofractionation (20 – 27 fractions)?<br><br>What percentage of your prostate patients are treated with SBRT (3 - 7 fractions)?<br><br>Does your center use gel spacers for prostate teletherapy?
QA for FIF beams	7/16/2014	Is measurement based QA for Forward Planned Field In Field (FIF) beams performed in your department?<br><br>If measurement based QA for FIF beams is performed, what device is used?<br><br>If measurement based QA for FIF is performed, who takes the measurements?
Radiation Oncology Workload	5/5/2013	In the past five years, how has the number of patients treated per year at your center changed?<br><br>What do you believe is the reason for the change? (select all that apply)<br><br>Has the use of hypofractionation changed the number of fractions for prostate patients?<br><br>Has the use of hypofractionation changed the number of fractions for breast patients?<br><br>Has the use of hypfractionation changed the number of fractions for bone metastasis patients?<br><br>What is your forecast for patient load at your center in five years?
Rectal Balloon - meddos	5/17/2013	How do you use a rectal balloon for prostate radiotherapy?
Rectal Balloon - medphys	5/17/2013	How do you use a rectal balloon for prostate radiotherapy?
Rectal Filling Management for Prostate Patients	5/19/2014	RTOG 0924 gives the following simulation innstructions for prostates. Which ones do you follow? (Check all that apply)

		How do you align the prostate before each fraction?<br><br>For those who use CBCT, how do you manage the patient who has substantial rectal filling on a given day?<br><br>For those who use CBCT, how do you manage the patient who has consistent rectal filling?
Replan for no bolus?	9/29/2015	Consider the following. A chestwall is planned with daily 0.5 cm flab bolus for 28 fractions. After 25 fractions, the physician decides to to stop the bolus. Would you replan?<br><br>Consider the following. A chestwall is planned with daily 0.5 cm flab bolus for 28 fractions. After 25 fractions, the physician decides to to stop the bolus. Would you require the physician to write a whole new prescription?
SBRT Immobilization	10/2/2013	Based on your experience, which immobilzation system would you purchase today for your Lung SBRT program? Please explain why in the "Other (please specify)" text box.
Setup Photos in Linac Vault	6/22/2017	Do you include Setup Photos in your Field Displays so the therapists can view them in the Linac Vault?
Sheets and Blankets and Pads - Oh My!	8/25/2014	When treating the patient, what do you allow the therapists to place over the treatment area for either warmth or modesty?<br><br>When treating the patient, what do you allow the therapists to place under the patient to improve comfort?<br><br>When treating chestwalls, how do you apply the bolus?
Shoulder Immobilization	6/11/2013	How do you immobilize the shoulders at the time of CT Simulation?<br><br>How do you immobilize the shoulders at the time of Treatment?
SIB for HN IMRT	5/12/2013	Does your center use Simultaneous Integrated Boost for Head and Neck IMRT cases?
SIB for IMRT	5/12/2013	Do you use Simultaneous Integrated Boost for your Head and Neck IMRT cases?
SIB for Prostate and PLN	12/16/2015	Do you use Simultaneous Integrated Boost when treating prostate plus pelvic lymph nodes?
Signing Consent	2/25/2014	At what time does the patient sign the Consent?<br><br>Who witnesses the signing of the Consent?<br><br>What sort of Consent form do you use?

SRS Dose Prescription	3/11/2016	What dose does your rad onc prescribe for tumors less than 20 mm in maximum diameter?
SRS Patient Alignment	4/24/2014	How do you align your SRS patient?
SRS: CT Contrast after MRI?	9/9/2015	Let's say you SRS patient had MRI exam with contrast. Would you then get a CT scan for planning with contrast?
Static Field Shape Verification	8/7/2016	Do you take initial portal images of the treatment fields with the patient on the table to verify the MLC shape for each static field?<br><br>After the patient has started, do you take subsequent portal images of the treatment fields?
Table Pads	9/21/2017	For Brain patients, do you use a table pad?<br><br>For Head/Neck patients, do you use a table pad?<br><br>For Lung patients, do you use a table pad?<br><br>For Pelvis patients, do you use a table pad?<br><br>For patients in pain, do you use a table pad?
Target for 4DCT	6/22/2015	How do you create your Internal Target Volume (ITV) for lung patients scanned with 4DCT?
TBI	6/24/2016	What is the status of TBI at your facility?<br><br>If you perform TBI, what is the beam arrangement?<br><br>If you do TBI, what is the patient position?<br><br>If you do TBI, what is your method of MU calculation?<br><br>If you do TBI, what is your In-Vivo Dosimetry status?<br><br>If you do TBI, what is your Compensator status? (choose all that apply)
TBI Roles	8/23/2017	For TBI, who participates in the initial simulation measurements? (check all that apply)<br><br>For TBI, who performs planning calculations? (check all that apply)<br><br>For TBI, who performs initial setup (including in-vivo measurements)? (check all that apply)<br><br>For TBI, who is present during each subsequent fraction? (check all that apply)
Testing for Linac Head Leakage	12/21/2015	Do you test for Head Leakage when accepting a new linac?

TG-51 Addendum	7/24/2017	Has your clinic implemented the addendum to the TG-51 protocol (McEwen et al., Med. Phys. 2014)?<br><br>If yes, what was the maximum calibration difference for any photon beam when switching from the original TG-51 protocol to the 2014 addendum?
Therapeutic MPAs	3/3/2017	Do you include MPAs in your practice (by that or some other name)?
Therapist Activities	4/24/2015	Which of the following treatment preparation activities do your therapists perform?<br><br>Which of the following QA activities do your therapists perform?<br><br>Which of the following treatment related activities do your therapists perform?<br><br>Which of the following HDR related activities do your therapists perform?<br><br>Which of the following weekly activities do your therapists perform?
Therapist Activities - meddos	5/11/2015	Which of the following treatment preparation activities do your therapists perform?<br><br>Which of the following QA activities do your therapists perform?<br><br>Which of the following treatment related activities do your therapists perform?<br><br>Which of the following HDR related activities do your therapists perform?<br><br>Which of the following weekly activities do your therapists perform?
Tomotherapy Independent MU Validation	3/1/2017	If you operate a Tomotherapy unit, do you perform an independent MU validation?
TPS and OIS	2/6/2013	Which of the following best describes your clinic?
Treating Facial Lesions	3/8/2014	What modality do you use to treat facial lesions?<br><br>If using electrons, how do you shape the field?<br><br>If using skin blocking, what is your method?<br><br>If you make a lead mask, what material do you use to make the facial impression?

		If you make a lead mask, what material do you use to make the mould?
Treatment Plan Evaluation Methods	9/21/2016	What method do you use to evaluate your treatment plans?
		What do you do with the results of the treatment plan evaluation? (select all that apply)
Treatment Plan QA	12/30/2014	Let's say Dosimetrist A completes a treatment plan. Who reviews the plan prior the the physician?
		After the physician approves the plan, who reviews the plan?
		Who pushes the plan to the secondary MU validation program (e.g. RadCalc)?
		Who calculates the secondary MU validation?
		Who approves the secondary MU validation results?
Turnaround time for planning	9/30/2014	In your department, what is the expected turnaround time for a physician to contour the CT dataset for a 3D plan?
	11/28/2016	In your department, what is the expected turnaround time for the dosimetrist to develop a 3D plan for the physician to review?
	3/10/2016	In your department, what is the expected turnaround time for a physician to contour the CT dataset for an IMRTplan?
	9/13/2014	In your department, what is the expected turnaround time for a dosimetrist to generate an IMRT plan for the physician to review?
VMAT SBRT using FFF Mode for Lung	6/11/2015	Does your site treat SBRT lung using VMAT under FFF mode?
		If Yes, do you reduce modulation during VMAT planning?
		If Yes, do you limit dose rate when treating?
		If Yes, do you use abdomen compression to limit respiration motion?
		If No, why not?
WBRT - Take 2	12/15/2015	How do you immobilize your WBRT patients?
		How do you plan your WBRT patients?
		What do you charge for WBRT planning?
		What do you charge for the WBRT treatment?
		Do you perform daily IGRT for your WBRT patients?

Weekly Rounds	9/16/2016	What is the nature of your Weekly Rounds? (select all that apply)
Who Attends Simulation?	10/27/2013	When do your radiation oncologists attend simulation for their patients?<br><br>When do your dosimetrists attend simulation?
Who does what - 2016	4/3/2013	At your center, who routinely does the image fusion?<br><br>At your center, who routinely does the IMRT/VMAT planning?<br><br>At your center, who routinely does the SBRT planning?<br><br>At your center, who routinely does the SRS planning?<br><br>At your center, who routinely does the HDR planning?<br><br>At your center, who routinely does the Prostate Seed Implant planning?<br><br>At your center, who routinely does the Weekly Chart Check?<br><br>At your center, who rotuinely does the extra work required for protocol patients?
Who does what for HDR?	6/22/2015	Who is responsible for taking measurements at Simulation?<br><br>Who is responsible for generating the HDR treatment plan?<br><br>Who is responsible for connecting the transfer tubes?<br><br>Who is responsible for initiating treatment at the Control Console?
Who does what?	5/26/2015	At your center, who routinely does the IMRT planning?<br><br>At your center, who routinely does the SBRT planning?<br><br>At your center, who routinely does the SRS planning?<br><br>At your center, who routinely does the HDR planning?<br><br>At your center, who routinely does the Prostate Seed Implant planning?
Whole Breast Contouring - meddos	12/15/2014	Inverse Planning for Whole Breast requires a Whole Breast PTV contour. At your facility, who creates the Whole Breast PTV contour?<br><br>How is the Whole Breast PTV contour created?
Winston Lutz for Lung SBRT	5/26/2015	Do you perform a WInston Lutz test before each Lung SBRT fraction?

Winston Lutz Test	12/15/2014	For SRS treatments, when do you perform the Winston Lutz test?<br><br>For SRT or SBRT treatments, when do you perform the Winston Lutz test?<br><br>Who performs the WInston Lutz test?<br><br>Does the test use film or the portal imager?<br><br>How are the images analyzed?<br><br>If using Cones and MLC the same day, which Winston Lutz test(s) do you perform?<br><br>What happens if IGRT (MV, KV, CBCT) indicates the anatomy does not line up?
Would you invest in hetero brachy software	10/25/2015	Would you purchase an algorithm that supports heterogeneity corrected dose calculations in HDR brachytherapy (e.g.Acuros BV) - today?

SURVEY DATA

3D Scanner or 1D Scanner plus 2D Array?

Q1. If you were starting a new program, which would you purchase for linac commissioning and annual QA?

Answer Choices	Responses		Uncertainty
3D Scanner	83.78%	62	±8%
1D Scanner plus 2D Array	16.22%	12	±8%
	**Answered**	74	
	**Skipped**	0	

3D Scanning System

Q1. If you were to purchase a 3D Scanning System today, which would you choose?

Answer Choices	Responses		Uncertainty
IBA Blue Phantom	26.15%	17	±12%
PTW MP3	24.62%	16	±12%
Sun Nuclear 3D Scanner	43.08%	28	±12%
Other (please specify)	6.15%	4	±9%
	**Answered**	65	
	**Skipped**	0	

6DOF Couch Top for Cranial SRS

Q1. For those of you who perform Cranial SRS, do you have a 6DoF Couch Top?

Answer Choices	Responses		Uncertainty
Yes	72.55%	37	±12%
No	27.45%	14	±12%
	**Answered**	51	
	**Skipped**	0	

Adaptive Radiotherapy

Q1. How often do your replan Head/Neck IMRT cases after they start?

Answer Choices	Responses	Uncertainty

Never	8.00%	2	±15%
Rarely	28.00%	7	±19%
Occasionally	56.00%	14	±19%
Often	4.00%	1	±13%
Systematically	4.00%	1	±13%
Other (please specify)		1	
	**Answered**	25	
	**Skipped**	0	

Q2. What are your criteria for replanning? (check all that apply)

Answer Choices	Responses		Uncertainty
Mask become loose	75.00%	18	±12%
SSD rule	20.83%	5	±7%
CBCT shows shrinking of external contour	70.83%	17	±12%
Patient loses certain amount of their initial weight	54.17%	13	±11%
Other (please specify)		1	±2%
	**Answered**	24	
	**Skipped**	1	

Q3. If you do not replan, what are the reasons? (check all that reply)

Answer Choices	Responses		Uncertainty
Cannot get reimbursed for replan	14.29%	1	±10%
Physicians are too busy to recontour	57.14%	4	±22%
Dosimetrists are too busy to replan	0.00%	0	±6%
We do not have Deformable Registration software	71.43%	5	±23%

Other (please specify)		3	±20%
	**Answered**	7	
	**Skipped**	18	

After Hours Radiotherapy

Q1. Does your facility provide radiotherapy for emergent patients after hours?

Answer Choices	Responses		Uncertainty
Yes	90.57%	48	±7%
No	9.43%	5	±7%
Other (please specify)		2	
	**Answered**	53	
	**Skipped**	1	

Q2. If no, how do you manage these patients? (Then skip to Q13)

Answer Choices	Responses		Uncertainty
Treat patient first thing the next clinical day	80.00%	4	±20%
Send patient to another facility which does emergent patients	20.00%	1	±25%
Other (please specify)		0	
	**Answered**	5	
	**Skipped**	49	

Q3. If yes, what is your type of facility?

Answer Choices	Responses		Uncertainty
Academic	14.58%	7	±13%
Community	62.50%	30	±13%
Free Standing	22.92%	11	±13%
Other (please specify)		1	
	**Answered**	48	
	**Skipped**	6	

Q4. How many emergent cases per year are treated?

Answer Choices	Responses		Uncertainty
1-4	38.30%	18	±14%
5-10	25.53%	12	±14%
11-20	17.02%	8	±14%
21-30	6.38%	3	±11%
31-40	6.38%	3	±11%
41-50	2.13%	1	±9%
> 50	4.26%	2	±10%
Other (please specify)		2	
	**Answered**	47	
	**Skipped**	7	

Q5. Which cases does your facility consider emergent? (check all the apply)

Answer Choices	Responses		Uncertainty
Cord Compression	100.00%	49	±7%
SVC Syndrome	91.84%	45	±7%
Whole Brain	53.06%	26	±5%
Rectal Bleeding	34.69%	17	±4%
Excruciating Bone Mets	53.06%	26	±5%
Other (please specify)		3	±2%
	**Answered**	49	
	**Skipped**	5	

Q6. Which personnel are on-call and required to attend the emergent treatment? (check all that apply)

Answer Choices	Responses		Uncertainty
Physician	95.92%	47	±8%
Therapist	95.92%	47	±8%
Dosimetrist	32.65%	16	±5%

Physicist	14.29%	7	±3%
Nurse	4.08%	2	±2%
Other (please specify)		10	±4%
	**Answered**	49	
	**Skipped**	6	

Q7. How is the patient prepared for treatment?

Answer Choices	Responses		Uncertainty
Machine Setup / Manual Calculation	56.41%	22	±15%
Machine Setup / Predetermined Monitor Units	5.13%	2	±11%
CT Simulated / Computer Planned	38.46%	15	±15%
Other (please specify)		13	
	**Answered**	39	
	**Skipped**	15	

Q8. If CT Simulated / Computer Planned, who does the planning?

Answer Choices	Responses		Uncertainty
Therapist	7.69%	2	±6%
Dosimetrist	80.77%	21	±16%
Physicist	30.77%	8	±13%
Other (please specify)		4	±9%
	**Answered**	26	
	**Skipped**	28	

Q9. How is the R&V component managed?

Answer Choices	Responses		Uncertainty
Pre-entered before treatment	52.08%	25	±14%
Captured on table	29.17%	14	±14%

Treat in Standby	18.75%	9	±14%
Other (please specify)		2	
	**Answered**	48	
	**Skipped**	6	

Q10. How is the imaging managed?

Answer Choices	Responses		Uncertainty
Rely only on port films	50.00%	22	±15%
Generate DRR's before treatment	50.00%	22	±15%
Other (please specify)		7	
	**Answered**	44	
	**Skipped**	10	

Q11. What is the availability during the week?

Answer Choices	Responses		Uncertainty
End of day - 9 PM	25.64%	10	±14%
End of day - Midnight	7.69%	3	±12%
End of day - Beginning of Next Day	66.67%	26	±14%
Other (please specify)		7	
	**Answered**	39	
	**Skipped**	15	

Q12. What is the availability on Saturday, Sunday, and Holidays?

Answer Choices	Responses		Uncertainty
8 AM - 5 PM	25.58%	11	±14%
8 AM - 9 PM	11.63%	5	±14%
8 AM - Midnight	0.00%	0	±8%
24 hours	62.79%	27	±14%
Other (please specify)		5	
	**Answered**	43	
	**Skipped**	11	

APBI Balloon Verification

Q1. How do you verify the APBI balloon volume and position before each fraction?

Answer Choices	Responses		Uncertainty
Radiograph with Simulator	7.44%	9	±9%
Radiograph with Portable	2.48%	3	±6%
Radiograph with Linac OBI	4.13%	5	±7%
CT Scanner	28.93%	35	±9%
CT Simulator	36.36%	44	±9%
CT with Linac CBCT	1.65%	2	±6%
Ultrasound	11.57%	14	±9%
Other	7.44%	9	±9%
Other (please specify)		22	
	**Answered**	121	
	**Skipped**	1	

Beam Energy for IMRT

Q1. How often do you use high energy photons (> 10 MV) for IMRT?

Answer Choices	Responses		Uncertainty
Never	61.67%	74	±9%
Pelvis only	13.33%	16	±9%
Whenever it makes the plan look better	25.00%	30	±9%
Other (please specify)		17	
	**Answered**	120	
	**Skipped**	10	

Beam Naming Convention

Q1. What convention do you use for beam naming?

Answer Choices	Responses		Uncertainty
Anatomy based (AP, RT LAT, LAO, etc)	45.00%	18	±16%
Machine based (G0, G90, G135, etc)	32.50%	13	±16%
Other (please specify)	22.50%	9	±16%
	**Answered**	40	
	**Skipped**	0	

Beam Naming Convention – medphys

Q1. What convention do you use for beam naming?

Answer Choices	Responses		Uncertainty
Anatomy based (AP, RT LAT, LAO, etc)	28.07%	32	±10%
Machine based (G0, G90, G135, etc)	43.86%	50	±10%
Other (please specify)	28.07%	32	±10%
	**Answered**	114	
	**Skipped**	0	

Beam Profiles

Q1. Which cross-beam profiles do you have in your planning system?

Answer Choices	Responses		Uncertainty
Profiles from the scanner with asymmetry included	28.07%	16	±13%
Profiles from the scanner with no asymmetry (centered & mirrored)	47.37%	27	±13%
Preconfigured beam data from vendor	24.56%	14	±13%
Other (please specify)		2	
	**Answered**	57	
	**Skipped**	0	

Q2. With regards to TG142 baseline symmetry, what is your baseline?

Answer Choices	Responses		Uncertainty
Original scanned data with asymmetry included	53.57%	30	±13%
Profiles in TPS with no asymmetry (if centered and mirrored)	33.93%	19	±13%
Preconfigured beam data provided by vendor	12.50%	7	±13%
Other (please specify)		3	
	**Answered**	56	
	**Skipped**	1	

Q3. When quoting symmetry, which evaluation are you using?

Answer Choices	Responses		Uncertainty
Central axis point difference	49.06%	26	±13%
Local point difference	11.32%	6	±13%
Point ratio	9.43%	5	±12%
Positive point difference	1.89%	1	±8%
Area average	13.21%	7	±13%
Area	15.09%	8	±13%
Other (please specify)		4	
	**Answered**	53	
	**Skipped**	4	

Brachytherapy for Cervix Cancer

Q1. If you perform brachytherapy for cervix cancer, are you in a hospital or free standing clinic?

Answer Choices	Responses	Uncertainty

Hospital	85.39%	76	±7%
Free Standing Center	14.61%	13	±7%
Other (please specify)		1	
	**Answered**	89	
	**Skipped**	0	

Q2. If you perform brachytherapy for cervix cancer, what modality do you use?

Answer Choices	Responses		Uncertainty
HDR with Ir-192	96.51%	83	±3%
HDR with Xoft	0.00%	0	±2%
LDR with Cs-137	3.49%	3	±3%
Other (please specify)		8	
	**Answered**	86	
	**Skipped**	3	

Q3. For those who use HDR, do you bring the patient to the OR for each fraction or just the first (assuming a Smit Sleeve is used?

Answer Choices	Responses		Uncertainty
Each Fraction	59.42%	41	±11%
First Fraction only	40.58%	28	±11%
Other (please specify)		20	
	**Answered**	69	
	**Skipped**	20	

Q4. Do you find the use of brachytherapy for cervical cancer to be increasing, staying the same, or decreasing?

Answer Choices	Responses		Uncertainty
Increasing	15.12%	13	±10%
Staying the same	66.28%	57	±10%
Decreasing	18.60%	16	±10%
Other (please specify)		4	
	**Answered**	86	
	**Skipped**	3	

Q5. What do you use for packing?

Answer Choices	Responses		Uncertainty
Gauze	87.01%	67	±7%
Radiadyne Alatus Vaginal Packing Balloon	12.99%	10	±7%
Other (please specify)		9	
	**Answered**	77	
	**Skipped**	12	

Q6. What imaging method do you use?

Answer Choices	Responses		Uncertainty
Orthogonal pair of 2D images	7.95%	7	±6%
CT imaging	88.64%	78	±6%
MR imaging	3.41%	3	±5%
Other (please specify)		12	
	**Answered**	88	
	**Skipped**	1	

Q7. What prescription method do you use?

Answer Choices	Responses		Uncertainty
Point A	56.47%	48	±11%
Volume based optimization	43.53%	37	±11%
Other (please specify)		10	
	**Answered**	85	
	**Skipped**	4	

Brass Mesh Bolus with 15X or 18X

Q1. Do you use brass mesh bolus for post-mastectomy chestwalls?

Answer Choices	Responses	Uncertainty

Yes	15.00%	3	±13%
No	85.00%	17	±12%
Other (please specify)		1	
	**Answered**	20	
	**Skipped**	1	

Q2. If yes, do you use it when one or more of the beams are 15X or 18X?

Answer Choices	Responses		Uncertainty
Yes	60.00%	3	±30%
No	40.00%	2	±36%
Other (please specify)		0	
	**Answered**	5	
	**Skipped**	16	

Breast Flash with VMAT

Q1. Do you use VMAT to treat Breast or Chestwall cases?

Answer Choices	Responses		Uncertainty
Yes	32.41%	35	±9%
No	61.11%	66	±9%
Other (please specify)	6.48%	7	±8%
	**Answered**	108	
	**Skipped**	0	

Q2. If Yes, do you somehow generate Flash around the external contuour?

Answer Choices	Responses		Uncertainty
Yes	67.39%	31	±13%
No	26.09%	12	±13%
Other (please specify)	6.52%	3	±11%
	**Answered**	46	
	**Skipped**	62	

Breast Planning Questions

Q1. What technique do you typically use when planning Right Breast Tangents?

Answer Choices	Responses		Uncertainty
Physical Wedges	4.35%	2	±8%
EDW, Virtual Wedge, OmniWedge	4.35%	2	±8%
Forward Planned Field In Field	73.91%	34	±11%
Electronic Compensator (Eclipse)	6.52%	3	±9%
Inverse Planned IMRT	2.17%	1	±7%
Hybrid (3D Base Dose + Inverse Planned IMRT)	8.70%	4	±10%
Other (please specify)		3	
	**Answered**	46	
	**Skipped**	0	

Q2. What technique do you typically use when planning Left Breast Tangents?

Answer Choices	Responses		Uncertainty
Physical Wedge	4.55%	2	±9%
EDW, Virtual Wedge, OmniWedge	4.55%	2	±9%
Forward Planned Field In Field	70.45%	31	±12%
Electronic Compensator (Eclispe)	6.82%	3	±10%
Inverse Planned IMRT	4.55%	2	±9%
Hybrid (3D Base Dose + Inverse Planned IMRT)	9.09%	4	±11%
Other (please specify)		6	
	**Answered**	44	
	**Skipped**	0	

Q3. What is the acceptable Hot Spot?

Answer Choices	Responses		Uncertainty
105.00%	18.60%	8	±15%
108.00%	55.81%	24	±15%
110.00%	23.26%	10	±15%
115.00%	2.33%	1	±9%
Other (please specify)		10	
	**Answered**	43	
	**Skipped**	3	

Q4. What is the acceptable Dose Coverage of the PTV?

Answer Choices	Responses		Uncertainty
100% of PTV gets Prescribed Dose	7.69%	3	±10%
95% of PTV gets Prescribe Dose	76.92%	30	±12%
93% of PTV gets Prescribed Dose	7.69%	3	±10%
90% of PTV gets Prescribed Dose	7.69%	3	±10%
Other (please specify)		10	
	**Answered**	39	
	**Skipped**	7	

Breast Planning Techniques

Q1. What technique do you typically use when planning Right Breast Tangents?

Answer Choices	Responses		Uncertainty
Physical Wedges	7.41%	4	±10%
EDW, Virtual Wedge, OmniWedge	14.81%	8	±10%
Forward Planned Field In Field	75.93%	41	±10%
Inverse Planned IMRT	1.85%	1	±7%
Other (please specify)		9	

**Answered**	54
**Skipped**	6

Q2. What technique do you typically use when planning Left Breast Tangents?

Answer Choices	Responses		Uncertainty
Physical Wedge	3.70%	2	±8%
EDW, Virtual Wedge, OmniWedge	11.11%	6	±11%
Forward Planned Field In Field	72.22%	39	±11%
Inverse Planned IMRT	12.96%	7	±11%
Other (please specify)		9	
	**Answered**	54	
	**Skipped**	6	

Q3. What percentage of your breast tangent patients are treated with Inverse Planned IMRT technique?

Answer Choices	Responses		Uncertainty
0.00%	59.32%	35	±12%
1% - 24%	30.51%	18	±12%
25% - 49%	1.69%	1	±7%
50% - 74%	5.08%	3	±9%
75% - 99%	3.39%	2	±8%
100.00%	0.00%	0	±6%
Other (please specify)		0	
	**Answered**	59	
	**Skipped**	1	

Breast Radiotherapy

Q1. Let us assume the "Canadian Protocol" for breast radiotherapy is 266 cGy x 16. How often do you use that fractionation scheme for your patients?

Answer Choices	Responses		Uncertainty
Never	24.19%	15	±12%

1% - 25% of patients	56.45%	35	±12%
26% - 50% of patients	14.52%	9	±12%
51% - 75% of patients	3.23%	2	±8%
76% - 100% of patients	1.61%	1	±7%
Other (please specify)		1	
	**Answered**	62	
	**Skipped**	1	

Q2. How do you typically boost these patients?

Answer Choices	Responses		Uncertainty
No Boost	41.38%	24	±13%
266 x 4	3.45%	2	±9%
250 x 4	24.14%	14	±13%
200 x 5	31.03%	18	±13%
Other (please specify)		2	
	**Answered**	58	
	**Skipped**	5	

Q3. What is your policy on using higher energy photons (15 MV, 18 MV) to reduce the hot spots?

Answer Choices	Responses		Uncertainty
Never use higher energy	12.96%	7	±13%
May mix up to 25% higher energy	29.63%	16	±13%
May mix up to 50% higher energy	37.04%	20	±13%
May mix up to 75% higher energy	3.70%	2	±9%
May use 100% higher energy	16.67%	9	±13%
Other (please specify)		11	
	**Answered**	54	
	**Skipped**	9	

Q4. What modality do you use for breast boosts?

Answer Choices	Responses		Uncertainty
Only electrons	8.06%	5	±10%
Primarily electrons	59.68%	37	±12%
Equal use of electrons or photons	20.97%	13	±12%
Primarily photons	8.06%	5	±10%
Only photons	3.23%	2	±8%
Other (please specify)		4	
	**Answered**	62	
	**Skipped**	1	

Checking SSDs – meddos

Q1. Do your therapists check and record the SSDs on the first fraction for non-IMRT fields?

Answer Choices	Responses		Uncertainty
Yes	87.50%	14	±9%
No	6.25%	1	±12%
Other (please specify)	6.25%	1	±12%
	**Answered**	16	
	**Skipped**	0	

Q2. Do your therapists check and record the SSDs on a weekly basis for non-IMRT fields?

Answer Choices	Responses		Uncertainty
Yes	68.75%	11	±20%
No	25.00%	4	±20%
Other (please specify)	6.25%	1	±14%
	**Answered**	16	
	**Skipped**	0	

Q3. Do you do the same for IMRT patients?

Answer Choices	Responses		Uncertainty

Yes	50.00%	8	±24%
No	25.00%	4	±24%
Other (please specify)	25.00%	4	±24%
	**Answered**	16	
	**Skipped**	0	

Q4. Do you do the same for IGRT patients?

Answer Choices	Responses		Uncertainty
Yes	66.67%	10	±21%
No	20.00%	3	±21%
Other (please specify)	13.33%	2	±18%
	**Answered**	15	
	**Skipped**	1	

Checking SSDs – medphysusa

Q1. Do your therapists check and record the SSDs on the first fraction for non-IMRT fields?

Answer Choices	Responses		Uncertainty
Yes	75.00%	42	±10%
No	17.86%	10	±10%
Other (please specify)	7.14%	4	±10%
	**Answered**	56	
	**Skipped**	0	

Q2. Do your therapists check and record the SSDs on a weekly basis for non-IMRT fields?

Answer Choices	Responses		Uncertainty
Yes	46.43%	26	±13%
No	37.50%	21	±13%
Other (please specify)	16.07%	9	±13%
	**Answered**	56	
	**Skipped**	0	

Q3. Do you do the same for IMRT patients?

Answer Choices	Responses		Uncertainty
Yes	70.91%	39	±11%
No	23.64%	13	±11%
Other (please specify)	5.45%	3	±9%
	**Answered**	55	
	**Skipped**	1	

Q4. Do you do the same for IGRT patients?

Answer Choices	Responses		Uncertainty
Yes	75.00%	42	±11%
No	23.21%	13	±11%
Other (please specify)	1.79%	1	±7%
	**Answered**	56	
	**Skipped**	0	

Chestwall Bolus Technique

Q1. What technique do you use for bolusing postmastectomy chestwalls?

Answer Choices	Responses		Uncertainty
0.5 cm flab every day	20.83%	5	±19%
0.5 cm flab every other day	54.17%	13	±19%
1.0 cm flab every day	12.50%	3	±17%
1.0 cm flab every other day	4.17%	1	±12%
1 layer of brass mesh bolus every day	4.17%	1	±12%
No bolus	4.17%	1	±12%
Other (please specify)		7	
	**Answered**	24	
	**Skipped**	5	

Q2. What happens if the skin reaction gets too severe?

Answer Choices	Responses		Uncertainty

Stop using the bolus immediately	48.15%	13	±18%
Give a break in treatment	33.33%	9	±18%
Stay the course because it will heal	3.70%	1	±13%
Switch to the Boost then finish with the remaining Tangents	14.81%	4	±18%
Other (please specify)		2	
	**Answered**	27	
	**Skipped**	2	

CMD Contouring

Q1. Do you contour the prostate for prostate cases?

Answer Choices	Responses		Uncertainty
Yes	21.90%	23	±8%
No	78.10%	82	±8%
	**Answered**	105	
	**Skipped**	2	

Q2. Do you contour the bladder for prostate cases?

Answer Choices	Responses		Uncertainty
Yes	92.52%	99	±4%
No	7.48%	8	±4%
	**Answered**	107	
	**Skipped**	0	

Q3. Do you contour the rectum for prostate cases?

Answer Choices	Responses		Uncertainty
Yes	89.72%	96	±5%
No	10.28%	11	±5%
	**Answered**	107	
	**Skipped**	0	

Q4. Do you contour the parotids for head/neck cases?

Answer Choices	Responses		Uncertainty
Yes	75.70%	81	±8%
No	24.30%	26	±8%
	**Answered**	107	
	**Skipped**	0	

Q5. Do you contour the healthy lung for mediastinal cases?

Answer Choices	Responses		Uncertainty
Yes	98.13%	105	±2%
No	1.87%	2	±2%
	**Answered**	107	
	**Skipped**	0	

Q6. Do you contour the spinal cord for all cases?

Answer Choices	Responses		Uncertainty
Yes	97.20%	104	±2%
No	2.80%	3	±3%
	**Answered**	107	
	**Skipped**	0	

Q7. Do you contour the optic chiasm for brain cases?

Answer Choices	Responses		Uncertainty
Yes	67.92%	72	±9%
No	32.08%	34	±9%
	**Answered**	106	
	**Skipped**	1	

CMD Contouring – 2016

Q1. At your center, who contours the normal anatomy and Organs At Risk?

Answer Choices	Responses		Uncertainty
Dosimetrist	91.07%	51	±6%

Physician	0.00%	0	±4%
Other (please specify)	8.93%	5	±6%
	**Answered**	56	
	**Skipped**	0	

Q2. At your center, who contours the nodal PTVs?

Answer Choices	Responses		Uncertainty
Dosimetrist	14.29%	8	±10%
Physician	75.00%	42	±10%
Other (please specify)	10.71%	6	±10%
	**Answered**	56	
	**Skipped**	0	

Q3. At your center, who creates the tumor PTV after the physician has drawn the GTV?

Answer Choices	Responses		Uncertainty
Dosimetrist	66.67%	32	±13%
Physician	33.33%	16	±13%
Other (please specify)		11	
	**Answered**	48	
	**Skipped**	8	

Q4. At your center, who creates the ITV for lung cases?

Answer Choices	Responses		Uncertainty
Dosimetrist	30.19%	16	±13%
Physicist	13.21%	7	±13%
Other (please specify)	56.60%	30	±13%
	**Answered**	53	
	**Skipped**	3	

Q5. At your center, who performs the Atlas Based Auto Segmentation?

Answer Choices	Responses		Uncertainty
Dosimetrist	66.67%	34	±12%

Physicist	3.92%	2	±9%
Other (please specify)	29.41%	15	±12%
	**Answered**	51	
	**Skipped**	5	

Coaching for DIBH Patients

Q1. If you provide coaching for DIBH treatments, what method do you use most often?

Answer Choices	Responses		Uncertainty
Verbal coaching	62.50%	20	±16%
Visual coaching with goggles	21.88%	7	±16%
Visual coaching with in room display	12.50%	4	±16%
Other (please specify)	3.13%	1	±11%
	**Answered**	32	
	**Skipped**	0	

Constancy Check of Electrometer-Chamber System

Q1. How do you perform a constancy check of your electrometer-chamber combination?

Answer Choices	Responses		Uncertainty
Sr-90 Well Check Source	32.04%	33	±9%
HDR Source	5.83%	6	±8%
Intercompare two independent systems	62.14%	64	±9%
Other (please specify)		11	
	**Answered**	103	
	**Skipped**	8	

Q2. How often do you perform a constancy check of your electrometer-chamber combination?

Answer Choices	Responses		Uncertainty
Annually	45.28%	48	±10%
Every Six Months	27.36%	29	±10%

Monthly	18.87%	20	±10%
Never	8.49%	9	±9%
Other (please specify)		5	
	**Answered**	106	
	**Skipped**	5	

Contrast for Prostate only CT Sim

Q1. For Prostate CT Sim, do you use IV contrast?

Answer Choices	Responses		Uncertainty
Yes	21.21%	7	±13%
No	78.79%	26	±13%
Other (please specify)		1	
	**Answered**	33	
	**Skipped**	2	

Q2. For Prostate CT Sim, do you use urethral administered contrast?

Answer Choices	Responses		Uncertainty
Yes	26.47%	9	±14%
No	73.53%	25	±14%
Other (please specify)		2	
	**Answered**	34	
	**Skipped**	1	

Q3. For Prostate CT SIm, do you use a Foley Catheter with contrast?

Answer Choices	Responses		Uncertainty
Yes	8.82%	3	±8%
No	91.18%	31	±7%
Other (please specify)		2	
	**Answered**	34	
	**Skipped**	1	

Q4. For Prostate CT Sim, do you use rectal contrast?

Answer Choices	Responses		Uncertainty
Yes	11.76%	4	±9%
No	88.24%	30	±9%
Other (please specify)		1	
	**Answered**	34	
	**Skipped**	1	

Q5. For Prostate CT Sim, do you use small bowel contrast?

Answer Choices	Responses		Uncertainty
Yes	11.76%	4	±9%
No	88.24%	30	±9%
Other (please specify)		0	
	**Answered**	34	
	**Skipped**	1	

Q6. For Prostate CT Sim, do you use a rectal balloon?

Answer Choices	Responses		Uncertainty
Yes	2.86%	1	±4%
No	97.14%	34	±3%
Other (please specify)		0	
	**Answered**	35	
	**Skipped**	0	

Copy of SIB for Prostate and PLN – meddos

Q1. Do you use Simultaneous Integrated Boost when treating prostate plus pelvic lymph nodes?

Answer Choices	Responses		Uncertainty
Yes	33.33%	4	±24%
No	66.67%	8	±24%
Other (please specify)		1	
	**Answered**	12	
	**Skipped**	0	

Cranial SRS Planning

Q1. When treating a unilateral cranial SRS target, do you use beams/arcs which enter through the contralateral side?

Answer Choices	Responses		Uncertainty
Never	7.59%	6	±10%
Infrequently	30.38%	24	±11%
Frequently	46.84%	37	±11%
Always	15.19%	12	±11%
Other (please specify)		3	
	**Answered**	79	
	**Skipped**	0	

Q2. Do you allow beams to pass through a critical structure provided the dose objectives are met?

Answer Choices	Responses		Uncertainty
Never	7.69%	6	±10%
Infrequently	56.41%	44	±11%
Frequently	33.33%	26	±11%
Never	2.56%	2	±7%
Other (please specify)		2	
	**Answered**	78	
	**Skipped**	1	

Creating Lung ITV with 4DCT – meddos

Q1. How do you create your Internal Target Volume (ITV) for lung patients scanned with 4DCT?

Answer Choices	Responses		Uncertainty
Maximum Intensity Projection (MIP) of all phases	48.39%	15	±18%
Average Intensity Projection (AveIP) of all phases	3.23%	1	±11%
Inspiration + Expiration contours integrated	12.90%	4	±16%

Contour GTV on each individual phase and integrate over all phases	35.48%	11	±18%
Other (please specify)		3	
	**Answered**	31	
	**Skipped**	1	

Cs-137 for GYN Brachytherapy

Q1. Does your hospital continue to utilize Cs-137 for GYN brachytherapy treatments?

Answer Choices	Responses		Uncertainty
No	75.58%	65	±9%
Yes	23.26%	20	±9%
Other (please specify)	1.16%	1	±5%
	**Answered**	86	
	**Skipped**	0	

Q2. If no, when did you discontinue its use?

Answer Choices	Responses		Uncertainty
< 5 years ago	28.36%	19	±11%
> 5 years ago	62.69%	42	±11%
Other (please specify)	8.96%	6	±11%
	**Answered**	67	
	**Skipped**	19	

Q3. If yes, when do you plan to discontinue using Cs-137?

Answer Choices	Responses		Uncertainty
< 1 year	13.64%	3	±18%
2-5 years	9.09%	2	±16%
5+ years	0.00%	0	±11%
Never	27.27%	6	±20%
Unknown	31.82%	7	±20%

Other (please specify)	18.18%	4	±20%
	**Answered**	22	
	**Skipped**	64	

Q4. If yes, do you run an HDR program in addition?

Answer Choices	Responses		Uncertainty
No	15.79%	6	±12%
Yes	76.32%	29	±12%
Other (please specify)	7.89%	3	±11%
	**Answered**	38	
	**Skipped**	48	

Q5. Do you solely rely on an HDR unit now for all GYN brachytherapy applications?

Answer Choices	Responses		Uncertainty
No	16.87%	14	±9%
Yes	74.70%	62	±9%
Other (please specify)	8.43%	7	±9%
	**Answered**	83	
	**Skipped**	3	

Dental Metal Artifacts

Q1. How do you manage Head/Neck patients who have substantial artifacts due to dental metal when running an IMRT/VMAT plan?

Answer Choices	Responses		Uncertainty
Contour the High Density and Low Density artifact regions and reassign the density (1 for tissue and 0 for air as needed).	75.00%	30	±12%
Run the plan with no density reassignments	10.00%	4	±12%
Run the plan in Homogeneous mode	0.00%	0	±7%

Other (please specify)	15.00%	6	±12%
	**Answered**	40	
	**Skipped**	0	

Do you assay seeds?

Q1. Do you assay any seeds when you receive an order from the vendor?

Answer Choices	Responses		Uncertainty
Yes	70.49%	43	±11%
No	29.51%	18	±11%
Other (please specify)		9	
	**Answered**	61	
	**Skipped**	2	

Q2. If you do assay seeds, how many do you assay?

Answer Choices	Responses		Uncertainty
a single seed	12.50%	2	±12%
ten percent of shipment	87.50%	14	±9%
Other (please specify)		29	
	**Answered**	16	
	**Skipped**	47	

Q3. If you assay seeds, how to you acquire the seeds you assay?

Answer Choices	Responses		Uncertainty
pull seed(s) from the group to be implanted	25.71%	9	±13%
order extra seed(s) from the same batch	74.29%	26	±13%
Other (please specify)		10	
	**Answered**	35	
	**Skipped**	28	

Dose Specification- water or medium?*

*For this survey, uncertainty was calculated for the Total column.

Q1. For photon plans, do you report “dose to water” or “dose to medium”?

	VarianAcuros		BrainLab		Elekta Monaco		Accuray Multiplan		Other		Total	Uncertainty
Dose to water	46.34%	19	17.07%	7	2.44%	1	4.88%	2	29.27%	12	41	±10%
Dose to medium	65.31%	32	4.08%	2	18.37%	9	2.04%	1	10.20%	5	49	±10%
Please specify Other											13	±10%
									**Answered**		84	
									**Skipped**		2	

Q2. For electron plans, do you report “dose to water” or “dose to medium”?

	VarianeMC		RayStation		Other		Total	Uncertainty
Dose to water	70.27%	26	5.41%	2	24.32%	9	37	±11%
Dose to medium	76.19%	32	2.38%	1	21.43%	9	42	±11%
Please specify Other							10	±11%
					**Answered**		76	
					**Skipped**		10	

Electron Calculations

Q1. How do you account for the Electron Cutout Factor used for MU calculations?

Answer Choices	Responses		Uncertainty
Measure each cutout individually	31.17%	24	±11%
Have a library of premeasured cutout factors	32.47%	25	±11%
Rely on RadCalc (for example) to calculate cutout factor	6.49%	5	±9%
Rely on treatment planning computer to	14.29%	11	±11%

determine cutout factor			
Ignore cutout factor except for cutouts smaller than a specific size	15.58%	12	±11%
Other (please specify)		14	
	**Answered**	77	
	**Skipped**	3	

Q2. If you measure individual Electron Cutout Factors, what is your method?

Answer Choices	Responses		Uncertainty
Ion chamber in soild water phantom	81.25%	52	±9%
Film in solid water phantom	1.56%	1	±6%
Diode or MOSFET under buildup	17.19%	11	±9%
Other (please specify)		6	
	**Answered**	64	
	**Skipped**	16	

Q3. Do you treat with the MU determined by a manual calculation or by the treatment planning computer?

Answer Choices	Responses		Uncertainty
Manual Calculation	75.64%	59	±9%
Treatment Planning System determination	24.36%	19	±9%
Other (please specify)		7	
	**Answered**	78	
	**Skipped**	2	

Q5. What is the maximum SSD which you would use calculated MU without phantom validation?

Answer Choices	Responses		Uncertainty
100	22.03%	13	±13%

103	3.39%	2	±9%
105	28.81%	17	±13%
110	45.76%	27	±13%
Other (please specify)		9	
	**Answered**	59	
	**Skipped**	21	

Electron Cutout Factors

Q1. How do you handle the MU Calculation when using an Electron Cutout?

Answer Choices	Responses		Uncertainty
Measure a specific cutout factor for each patient	38.98%	23	±13%
Use a lookup table of cutout factors	37.29%	22	±13%
Do not use cutout factor if cutout exceeds certain size	23.73%	14	±13%
Other (please specify)		13	
	**Answered**	59	
	**Skipped**	8	

Electron MUs and Isodose Plans

Q1. How do you determine the MU to be used for electron treatments?

Answer Choices	Responses		Uncertainty
Use MU from Independent Calculation (Hand Calc, RadCalc, IMSure, etc)	86.49%	32	±10%
Use MU from Eclipse Monte Carlo plan	10.81%	4	±10%
Use MU from Pinnacle plan	0.00%	0	±5%
Use MU from Xio plan	2.70%	1	±7%

Other (please specify)		3	
	**Answered**	37	
	**Skipped**	2	

Q2. What type of Isodose Plan do you generate for the chart?

Answer Choices	Responses		Uncertainty
Hetero plan using MU from Independent calculation	21.62%	8	±16%
Hetero plan using MU from Treatment Planning System	54.05%	20	±16%
Homo plan using MU from Independent calculation	8.11%	3	±13%
Homo plan using MU from Treatment Planning System	16.22%	6	±16%
Other (please specify)		5	
	**Answered**	37	
	**Skipped**	2	

Q3. How is the Isodose Plan presented?

Answer Choices	Responses		Uncertainty
Absolute Dose	52.78%	19	±16%
Relative Dose	47.22%	17	±16%
Other (please specify)		2	
	**Answered**	36	
	**Skipped**	3	

Electron MUs and Isodose Plans – 2016

Q1. How do you determine the MU to be used for electron treatments?

Answer Choices	Responses		Uncertainty
Use MU from Independent	45.69%	53	±9%

Calculation (Hand Calc, RadCalc, IMSure, etc)			
Use MU from Eclipse Monte Carlo plan	45.69%	53	±9%
Use MU from Eclipse AAA Plan	2.59%	3	±6%
Use MU from Pinnacle plan	2.59%	3	±6%
Use MU from Xio plan	3.45%	4	±7%
Other (please specify)		14	
	**Answered**	116	
	**Skipped**	8	

Q2. What type of Isodose Plan do you generate for the chart?

Answer Choices	Responses		Uncertainty
Hetero plan using MU from Independent calculation	14.88%	18	±7%
Hetero plan using MU from Treatment Planning System	73.55%	89	±7%
Homo plan using MU from Independent calculation	5.79%	7	±7%
Homo plan using MU from Treatment Planning System	5.79%	7	±7%
Other (please specify)		4	
	**Answered**	121	
	**Skipped**	3	

Q3. How is the Isodose Plan presented?

Answer Choices	Responses		Uncertainty
Absolute Dose	68.85%	84	±8%
Relative Dose	31.15%	38	±8%

Other (please specify)		1
	**Answered**	122
	**Skipped**	2

FFF beams for Breast Tangents

Q1. For those of you with TrueBeam, do you use FFF mode for breast tangents to reduce the treatment time for DIBH techniques?

Answer Choices	Responses		Uncertainty
Yes	0.00%	0	±3%
No	100.00%	26	±0%
Other (please specify)		1	
	**Answered**	26	
	**Skipped**	0	

Fiducial Markers

Q1. Do you use fiducial markers for IGRT?

Answer Choices	Responses		Uncertainty
Yes	67.53%	52	±10%
No	28.57%	22	±10%
Other (please specify)	3.90%	3	±8%
	**Answered**	77	
	**Skipped**	1	

Q2. If yes, what type of fiducial marker do you use?

Answer Choices	Responses		Uncertainty
Gold	80.39%	41	±10%
Polymer	5.88%	3	±9%
Carbon	1.96%	1	±7%
Stainless Steel	3.92%	2	±8%
Titanium	0.00%	0	±6%
Other (please specify)	7.84%	4	±10%
	**Answered**	51	

**Skipped**	27

Q3. If yes, what design of fiducial marker do you use?

Answer Choices	Responses		Uncertainty
Individual Seeds	68.75%	33	±12%
Individual Coils	18.75%	9	±12%
Linked Seeds	4.17%	2	±9%
"Gold Anchor"	8.33%	4	±11%
Other (please specify)	0.00%	0	±7%
	**Answered**	48	
	**Skipped**	30	

Q4. If yes, what vendor supplies your fiducial markers?

Answer Choices	Responses		Uncertainty
Best Medical	14.63%	6	±15%
CIVCO	41.46%	17	±15%
Cortex	2.44%	1	±9%
Gold Anchor	4.88%	2	±10%
iba Dosimetry	19.51%	8	±15%
JRT Associates	0.00%	0	±8%
Other (please specify)	17.07%	7	±15%
	**Answered**	41	
	**Skipped**	37	

Filming IMRT Fields

Q1. Do you film IMRT fields with the patient on the table?

Answer Choices	Responses		Uncertainty
Yes	15.79%	6	±10%
No	84.21%	32	±10%
	**Answered**	38	
	**Skipped**	1	

Q2. If yes, do you film all the fields or only a sample?

Answer Choices	Responses		Uncertainty
All	60.00%	3	±30%
Some	40.00%	2	±36%
Other (please specify)		5	
	**Answered**	5	
	**Skipped**	34	

Q3. If you do not film the IMRT fields, what is your rationale?

Answer Choices	Responses		Uncertainty
IMRT fields verified by 2D Array	57.69%	15	±18%
IMRT fields verified by Portal Imaging	42.31%	11	±18%
Other (please specify)		8	
	**Answered**	26	
	**Skipped**	13	

Frame vs Frameless SRS

Q1. If you perform Cranial SRS, do use a Frame (pins into skull) or Frameless (no pins into skull) system?

Answer Choices	Responses		Uncertainty
Frame	6.25%	1	±8%
Frameless	93.75%	15	±6%
Other (please specify)		1	
	**Answered**	16	
	**Skipped**	0	

Frame vs Frameless SRS – medphys

Q1. If you perform Cranial SRS, do use a Frame (pins into skull) or Frameless (no pins into skull) system?

Answer Choices	Responses		Uncertainty
Frame	14.46%	12	±7%
Frameless	85.54%	71	±7%

Other (please specify)		11	
	**Answered**	83	
	**Skipped**	7	

Gafchromic Film

Q1. What type of Gafchromic film do you use for Light/Radiation QA?

Answer Choices	Responses		Uncertainty
EBT2	17.24%	5	±18%
EBT3	44.83%	13	±18%
EBT-XD	0.00%	0	±10%
RTQA2	37.93%	11	±18%
Other (please specify)		5	
	**Answered**	29	
	**Skipped**	7	

Q2. What type of Gafchromic film do you use for Picket Fence QA?

Answer Choices	Responses		Uncertainty
EBT2	22.73%	5	±21%
EBT3	45.45%	10	±21%
EBT-XD	0.00%	0	±12%
RTQA2	31.82%	7	±21%
Other (please specify)		9	
	**Answered**	22	
	**Skipped**	14	

Q3. What type of Gafchromic film do you use for IMRT QA?

Answer Choices	Responses		Uncertainty
EBT2	12.50%	3	±12%
EBT3	83.33%	20	±12%
EBT-XD	4.17%	1	±10%
RTQA2	0.00%	0	±8%

Other (please specify)		6
	**Answered**	24
	**Skipped**	12

Q4. What type of Gafchromic Film do you use for SRS QA?

Answer Choices	Responses		Uncertainty
EBT2	9.68%	3	±14%
EBT3	67.74%	21	±15%
EBT-XD	19.35%	6	±15%
RTQA2	0.00%	0	±9%
MD-V3	3.23%	1	±10%
HD-V2	0.00%	0	±9%
Other (please specify)		3	
	**Answered**	31	
	**Skipped**	5	

Q6. Do you analyze in relative or absolute dose?

Answer Choices	Responses		Uncertainty
Relative	27.27%	9	±14%
Absolute	72.73%	24	±14%
Other (please specify)		3	
	**Answered**	33	
	**Skipped**	3	

Gel Phantoms

Q1. Would you be interested in using a Gel Phantom for dosimetry validation?

Answer Choices	Responses		Uncertainty
Yes	84.38%	27	±11%
No	12.50%	4	±11%
Other (please specify)	3.13%	1	±8%
	**Answered**	32	
	**Skipped**	0	

Q2. If yes, what applications would you be testing? (check all that apply)

Answer Choices	Responses		Uncertainty
SRS	85.71%	24	±9%
SBRT	64.29%	18	±8%
IMRT	53.57%	15	±7%
VMAT	64.29%	18	±8%
Electrons	10.71%	3	±3%
Protons	14.29%	4	±3%
HDR Brachytherapy	32.14%	9	±5%
LDR Brachytherapy	0.00%	0	±1%
Other (please specify)	7.14%	2	±2%
	**Answered**	28	
	**Skipped**	4	

Q3. If yes, how would you use such a phantom? (check all the apply)

Answer Choices	Responses		Uncertainty
Initial Commissioning	75.86%	22	±13%
Patient Specific QA	51.72%	15	±11%
Annual QA	58.62%	17	±12%
Other (please specify)	3.45%	1	±2%
	**Answered**	29	
	**Skipped**	3	

Q4. If yes, which type of solution would you prefer?

Answer Choices	Responses		Uncertainty
Single use phantom (such as RPC phantom)	28.57%	8	±17%
In-house Analysis System	64.29%	18	±17%
Other (please specify)	7.14%	2	±13%
	**Answered**	28	

**Skipped**	4

Q5. If no, why not?

Answer Choices	Responses		Uncertainty
Happy with current solutions	40.00%	4	±24%
Cost concerns	60.00%	6	±25%
Other (please specify)	20.00%	2	±16%
	**Answered**	10	
	**Skipped**	22	

Generating Composite Dose Distributions

Q1. When your clinic encounters a patient with prior radiation does, is a composite isodose distribution generated?

Answer Choices	Responses		Uncertainty
Yes	87.20%	143	±5%
No	12.80%	21	±5%
Comments		34	
	**Answered**	164	
	**Skipped**	3	

Q2. If your clinic generates composite isodose distributions, how is it accomplished?

Answer Choices	Responses		Uncertainty
DIR Software with CT to CT fusion	26.06%	37	±8%
Planning System with CT to Ct fusion	53.52%	76	±8%
Manual (eyeball) placement of prior isocenter point and import of RT plan to that isocenter	20.42%	29	±8%
Comments		25	
	**Answered**	142	

**Skipped**	26

Q3. In general which method of generating a composite isodose distribution is most accurate?

Answer Choices	Responses		Uncertainty
CT to CT fusion	88.65%	125	±5%
Manual (eyeball) placement of prior isocenter point and import of RT plan to that isocenter	11.35%	16	±5%
Comments		14	
	**Answered**	141	
	**Skipped**	26	

Q4. If your Clinic generates composite isodose distributions, who is responsible for the work?

Answer Choices	Responses		Uncertainty
Dosimetrist	55.94%	80	±8%
Physicist	35.66%	51	±8%
Other (please specify)	8.39%	12	±8%
Comments		27	
	**Answered**	143	
	**Skipped**	24	

Q5. Do you bill a Special Physics Consult 77370 for composite isodose distributions?

Answer Choices	Responses		Uncertainty
Yes	32.26%	40	±9%
No	53.23%	66	±9%
Sometimes (please explain)	14.52%	18	±9%
Comments		28	
	**Answered**	124	
	**Skipped**	43	

Q6. If using DIR software do you bill a Special Physics Consult 77370 for deformable image registration?

Answer Choices	Responses		Uncertainty
Yes	25.27%	23	±9%
No	70.33%	64	±9%
Sometimes (please explain)	4.40%	4	±7%
Comments		13	
	**Answered**	91	
	**Skipped**	76	

GTV-PTV for SRS

Q1. For Cranial SRS, what margin around the GTV do you use for creating the PTV?

Answer Choices	Responses		Uncertainty
0 mm	31.82%	14	±15%
1 mm	38.64%	17	±15%
2 mm	29.55%	13	±15%
Other (please specify)		8	
	**Answered**	44	
	**Skipped**	4	

Hard Wedges – medphys

Q1. Do you still use Hard Wedges for any patient treatments?

Answer Choices	Responses		Uncertainty
Yes	51.85%	42	±11%
No	48.15%	39	±11%
Other (please specify)		7	
	**Answered**	81	
	**Skipped**	4	

HDR Source Activity

Q1. What HDR Source Activity do you enter into the treatment planning system and control console?

Answer Choices	Responses		Uncertainty

Manufacturer's Certificate value	69.88%	116	±7%
User's measured value	27.71%	46	±7%
Other (please specify)	2.41%	4	±5%
	**Answered**	166	
	**Skipped**	0	

Higher Photon Energy

Q1. If you could alter the energy pair on all your linacs to one standard, what would you choose?

Answer Choices	Responses		Uncertainty
6X / 10X	45.24%	38	±11%
6X / 12X	7.14%	6	±9%
6X / 15X	36.90%	31	±11%
6X / 18X	5.95%	5	±9%
Other	4.76%	4	±8%
Other (please specify)		10	
	**Answered**	84	
	**Skipped**	3	

Humeral Head Blocking

Q1. When treating three field breasts, how does your radiation oncologist block the humeral head?

Answer Choices	Responses		Uncertainty
Does not block humeral head	4.17%	1	±12%
Block 1/3 of humeral head	0.00%	0	±10%
Block 1/2 of humeral head	0.00%	0	±10%
Block 2/3 on humeral head	25.00%	6	±18%
Block entire humeral head	62.50%	15	±18%

Block entire humeral head plus margin	8.33%	2	±14%
Other (please specify)		2	
	**Answered**	24	
	**Skipped**	1	

Q2. What does your radiation oncologist say is the primary reason to block the humeral head?

Answer Choices	Responses		Uncertainty
Prevent fibrosis which can cause shoulder immobility	71.43%	15	±17%
Prevent lymphadema in the arm	9.52%	2	±15%
Protect bone marrow	19.05%	4	±17%
Other (please specify)		5	
	**Answered**	21	
	**Skipped**	4	

Hybrid IMRT and QA

Q1. Given the stated definition of Hybrid IMRT, would you perform QA for these plans?

Answer Choices	Responses		Uncertainty
Yes	42.50%	17	±16%
No	45.00%	18	±16%
Other (please specify)	12.50%	5	±14%
	**Answered**	40	
	**Skipped**	0	

Hypofractionation for Prostate

Q1. Does you center use standard fractionation such as 38-43 fractions for prostates?

Answer Choices	Responses		Uncertainty
Yes	68.00%	17	±17%
No	16.00%	4	±17%
Other (please specify)	16.00%	4	±17%

**Answered**	25
**Skipped**	0

Q2. If no, how many fractions do you use?

Answer Choices	Responses		Uncertainty
30	0.00%	0	±17%
28	14.29%	1	±24%
5	28.57%	2	±31%
Other (please specify)	57.14%	4	±31%
	**Answered**	7	
	**Skipped**	18	

IGRT for Breast Tangents

Q1. Which imaging protocol do you follow for breast tangents?

Answer Choices	Responses		Uncertainty
Weekly MV Ports alone	82.35%	28	±11%
Weekly MV Ports plus Daily KV Setup Pair	11.76%	4	±11%
Daily Setup Pair alone	5.88%	2	±10%
Weekly MV Ports plus Daily CBCT	0.00%	0	±7%
Daily CBCT alone	0.00%	0	±7%
We do not have a KV Imager	0.00%	0	±7%
Other (please specify)		7	d
	**Answered**	34	
	**Skipped**	5	

IGRT for Frameless SRS

Q1. If you perform frameless SRS on a linac, what IGRT technique do you use?

Answer Choices	Responses		Uncertainty
ExacTrac	42.47%	31	±12%

CBCT	47.95%	35	±12%
KV-KV	9.59%	7	±11%
Other (please specify)		9	
	**Answered**	73	
	**Skipped**	0	

IGRT Shift Limits

Q1. What is the maximum shift your therapists allowed to make independently?

Answer Choices	Responses		Uncertainty
1 cm	67.50%	27	±13%
2 cm	15.00%	6	±13%
3 cm	7.50%	3	±11%
4 cm	0.00%	0	±7%
Any shift deemed necessary	10.00%	4	±12%
Other (please specify)		12	
	**Answered**	40	
	**Skipped**	12	

Q2. If there is a shift limit, who must be called to approve it?

Answer Choices	Responses		Uncertainty
Chief Therapist	4.55%	2	±3%
Dosimetrist	15.91%	7	±6%
Physicist	45.45%	20	±10%
Physician	79.55%	35	±12%
Other (please specify)		6	±6%
	**Answered**	44	
	**Skipped**	8	

Q3. Have you ever had a treatment event because an inappropriate shift was applied?

Answer Choices	Responses		Uncertainty
Yes	28.57%	14	±12%

No	71.43%	35	±12%
Other (please specify)		3	
	**Answered**	49	
	**Skipped**	3	

Implementation of 10X FFF

Q1. For those of you with 10X FFF, have you commissioned it?

Answer Choices	Responses		Uncertainty
Yes	86.44%	51	±8%
No	13.56%	8	±8%
	**Answered**	59	
	**Skipped**	0	

Q2. If yes, do you find it is sometimes superior to 6X FFF?

Answer Choices	Responses		Uncertainty
Yes	56.86%	29	±13%
No	13.73%	7	±13%
Other (please specify)	29.41%	15	±13%
	**Answered**	51	
	**Skipped**	8	

Implementation of 10X FFF – meddos

Q1. For those of you with 10X FFF, have you commissioned it?

Answer Choices	Responses		Uncertainty
Yes	88.89%	8	±11%
No	11.11%	1	±15%
	**Answered**	9	
	**Skipped**	0	

Q2. If yes, do you find it is sometimes superior to 6X FFF?

Answer Choices	Responses		Uncertainty
Yes	50.00%	4	±32%

No	25.00%	2	±32%
Other (please specify)	25.00%	2	±32%
	**Answered**	8	
	**Skipped**	1	

IMRT Boost Techniques – meddos

Q1. How do you boost Prostate cases?

Answer Choices	Responses		Uncertainty
Sequential Boost	89.19%	33	±8%
Simultaneous Integrated Boost	10.81%	4	±8%
Comment		4	
	**Answered**	37	
	**Skipped**	2	

Q2. How do your boost Head and Neck cases?

Answer Choices	Responses		Uncertainty
Sequential Boost	26.32%	10	±13%
Simultaneous Integrated Boost	73.68%	28	±13%
Comment		3	
	**Answered**	38	
	**Skipped**	1	

Q3. How do you boost Brain cases?

Answer Choices	Responses		Uncertainty
Sequential Boost	86.84%	33	±9%
Simultaneous Integrated Boost	13.16%	5	±9%
Comment		2	
	**Answered**	38	
	**Skipped**	1	

Q4. How do you boost Lung cases?

Answer Choices	Responses		Uncertainty
Sequential Boost	77.78%	28	±13%
Simultaneous Integrated Boost	22.22%	8	±13%
Comment		3	
	**Answered**	36	
	**Skipped**	3	

Q5. How do you boost Breast cases?

Answer Choices	Responses		Uncertainty
Sequential Boost	94.74%	36	±5%
Simultaneous Integrated Boost	5.26%	2	±5%
Comment		1	
	**Answered**	38	
	**Skipped**	1	

Q6. If you use Sequential Boosts, do you run the boost plan at the start or later in the course?

Answer Choices	Responses		Uncertainty
At the start	68.57%	24	±14%
Later in the course	31.43%	11	±14%
Comment		8	
	**Answered**	35	
	**Skipped**	4	

Q7. If you use Sequential Boosts, do you QA the boost plan at the start or later in the course?

Answer Choices	Responses		Uncertainty
At the start	31.43%	11	±14%
Later in the course	68.57%	24	±14%
Comment		4	
	**Answered**	35	
	**Skipped**	4	

IMRT Boost Techniques – medphys

Q1. How do you boost Prostate cases?

Answer Choices	Responses		Uncertainty
Sequential Boost	73.91%	68	±9%
Simultaneous Integrated Boost	26.09%	24	±9%
Comment		12	
	**Answered**	92	
	**Skipped**	3	

Q2. How do your boost Head and Neck cases?

Answer Choices	Responses		Uncertainty
Sequential Boost	25.84%	23	±9%
Simultaneous Integrated Boost	74.16%	66	±9%
Comment		7	
	**Answered**	89	
	**Skipped**	6	

Q3. How do you boost Brain cases?

Answer Choices	Responses		Uncertainty
Sequential Boost	65.06%	54	±10%
Simultaneous Integrated Boost	34.94%	29	±10%
Comment		6	
	**Answered**	83	
	**Skipped**	12	

Q4. How do you boost Lung cases?

Answer Choices	Responses		Uncertainty
Sequential Boost	72.15%	57	±10%

Simultaneous Integrated Boost	27.85%	22	±10%
Comment		13	
	**Answered**	79	
	**Skipped**	16	

Q5. How do you boost Breast cases?

Answer Choices	Responses		Uncertainty
Sequential Boost	79.07%	68	±8%
Simultaneous Integrated Boost	20.93%	18	±8%
Comment		12	
	**Answered**	86	
	**Skipped**	9	

Q6. If you use Sequential Boosts, do you run the boost plan at the start or later in the course?

Answer Choices	Responses		Uncertainty
At the start	53.93%	48	±10%
Later in the course	46.07%	41	±10%
Comment		13	
	**Answered**	89	
	**Skipped**	6	

Q7. If you use Sequential Boosts, do you QA the boost plan at the start or later in the course?

Answer Choices	Responses		Uncertainty
At the start	50.00%	42	±11%
Later in the course	50.00%	42	±11%
Comment		9	
	**Answered**	84	
	**Skipped**	11	

IMRT/VMAT planning and Air Cavities

Q1. For Head-Neck IMRT/VMAT planning, how do you handle air cavities such as sinuses, oral cavity, etc?

Answer Choices	Responses		Uncertainty
No modification	74.03%	57	±9%
Contour and assign tissue density	5.19%	4	±8%
Rely on Eclipse Air Cavity Correction	12.99%	10	±9%
Other (please specify)	7.79%	6	±9%
	**Answered**	77	
	**Skipped**	1	

Q2. For Pelvis IMRT/VMAT planning, how do you handle air cavities due to flatulence?

Answer Choices	Responses		Uncertainty
No modification	65.38%	51	±10%
Contour and assign tissue density	17.95%	14	±10%
Rely on Eclipse Air Cavity Correction	8.97%	7	±10%
Other (please specify)	7.69%	6	±9%
	**Answered**	78	
	**Skipped**	0	

IMRT/VMAT planning and Air Cavities – meddos

Q1. For Head-Neck IMRT/VMAT planning, how do you handle air cavities such as sinuses, oral cavity, etc?

Answer Choices	Responses		Uncertainty
No modification	72.97%	27	±13%
Contour and assign tissue density	5.41%	2	±10%
Rely on Eclipse Air Cavity Correction	13.51%	5	±13%
Other (please specify)	8.11%	3	±12%
	**Answered**	37	

**Skipped**	0

Q2. For Pelvis IMRT/VMAT planning, how do you handle air cavities due to flatulence?

Answer Choices	Responses		Uncertainty
No modification	64.86%	24	±14%
Contour and assign tissue density	5.41%	2	±10%
Rely on Eclipse Air Cavity Correction	16.22%	6	±14%
Other (please specify)	13.51%	5	±14%
	**Answered**	37	
	**Skipped**	0	

IMRT/VMAT QA - Why?

Q1. Have you ever changed a plan based on the IMRT/VMAT QA?

Answer Choices	Responses		Uncertainty
Yes	68.57%	24	±14%
No	31.43%	11	±14%
	**Answered**	35	
	**Skipped**	0	

IMRT/VMAT QA Methods

Q1. Which method do you feel is the best available today for IMRT/VMAT QA?

Answer Choices	Responses		Uncertainty
2D Detector Based	12.66%	20	±8%
3D Detector Based	51.27%	81	±8%
EPID Based	20.25%	32	±8%
Film Based	1.27%	2	±5%
Logfile Base	2.53%	4	±5%
Logfile Based with Point Measurements	0.63%	1	±4%

Logfile Based with EPID Measurements	8.86%	14	±8%
Point Measurement Based	0.00%	0	±4%
Other	2.53%	4	±5%
Reason for choice		67	
	**Answered**	158	
	**Skipped**	0	

In Vivo Dosimetry

Q1. Do you perform ROUTINE in vivo dosimetry measurements (i.e. on patient during actual treatment) for QA purposes?

Answer Choices	Responses		Uncertainty
Yes	46.67%	42	±10%
No	53.33%	48	±10%
	**Answered**	90	
	**Skipped**	0	

Q2. If yes, for which of the following treatment types do you use ROUTINE in vivo dosimetry for QA? (click all that apply)

Answer Choices	Responses		Uncertainty
Electron fields	55.32%	26	±9%
Dynamic MLC fields	23.40%	11	±8%
Dynamic Arc fields	12.77%	6	±5%
Brachytherapy	0.00%	0	±4%
SRS	6.38%	3	±1%
SBRT	10.64%	5	±3%
TBI	21.28%	10	±4%
TSE	10.64%	5	±5%
Other (please specify)	14.89%	7	±4%
	**Answered**	47	
	**Skipped**	43	

Q3. If yes, what is the MAIN reason you perform ROUTINE in vivo dosimetry?

Answer Choices	Responses		Uncertainty
It is a valuable QA tool	38.30%	18	±14%
Legal defense	12.77%	6	±14%
Revenue	6.38%	3	±11%
Continuation of historical practice	19.15%	9	±14%
Institute policy	10.64%	5	±13%
Other (please specify)	12.77%	6	±14%
	**Answered**	47	
	**Skipped**	43	

Q4. If yes, do you bill for this service?

Answer Choices	Responses		Uncertainty
Yes	58.33%	28	±14%
No	22.92%	11	±14%
Does not apply because we are beyond the USA border	18.75%	9	±14%
	**Answered**	48	
	**Skipped**	42	

Invivo Dosimetry

Q1. What do you use for invivo dosimetry?

Answer Choices	Responses		Uncertainty
LiF TLD with inhouse reader	9.78%	9	±10%
LiF TLD read by vendor	1.09%	1	±6%
OSLD with inhouse reader	18.48%	17	±10%
OSLD read by Landauer	6.52%	6	±9%

Diode	23.91%	22	±10%
MOSFET	18.48%	17	±10%
Do not perform measurements	8.70%	8	±10%
Other (please specify)	13.04%	12	±10%
	**Answered**	92	
	**Skipped**	0	

IV contrast for CT Sim

Q1. When do you use IV contrast for CT Simulation? (Select all that apply)

Answer Choices	Responses		Uncertainty
Never	37.50%	15	±8%
Head/Neck	60.00%	24	±9%
Prostate alone	10.00%	4	±4%
Prostate plus Pelvic Lymph Nodes	30.00%	12	±7%
GYN plus Pelvic Lymph Nodes	37.50%	15	±8%
Other (please specify)		14	±7%
	**Answered**	40	
	**Skipped**	1	

Q2. When contrast is used, how do you then plan?

Answer Choices	Responses		Uncertainty
Ignore the contrast	25.81%	8	±18%
Use two scans - contour on contrast scan, fuse the scans, plan on no contrast scan	41.94%	13	±18%
Use a specialized CT# / Density Table to set contrast density to 1.0	32.26%	10	±18%
Other (please specify)		3	

**Answered**	31
**Skipped**	10

IVD for 2D and 3D Routine Treatment Delivery QA

Q1. For 2D and 3D Photon treatments, do you make routine vivo dosimetry measurements of the delivered dose to the patient?

Answer Choices	Responses		Uncertainty
Yes	14.75%	27	±5%
No	79.78%	146	±5%
Other (please specify)	5.46%	10	±5%
	**Answered**	183	
	**Skipped**	0	

Q2. For Electron treatments, do you make routine vivo dosimetry measurements of the delivered dose to the patient?

Answer Choices	Responses		Uncertainty
Yes	18.58%	34	±6%
No	75.41%	138	±6%
Other (please specify)	6.01%	11	±6%
	**Answered**	183	
	**Skipped**	0	

IVD for Treatment Delivery QA

Q1. For IMRT/VMAT treatments, do you make vivo dosimetry measurements of the delivered dose to the patient?

Answer Choices	Responses		Uncertainty
Yes	27.27%	15	±12%
No	65.45%	36	±12%
Other (please specify)	7.27%	4	±10%
	**Answered**	55	
	**Skipped**	0	

Q2. If yes, what type of vivo measurements do you make?

Answer Choices	Responses		Uncertainty
TLD Point Dose	10.53%	2	±18%
Diode Point Dose	15.79%	3	±21%
MOSFET Point Dose	21.05%	4	±21%
EPID Transit Dosimetry	31.58%	6	±21%
Other (please specify)	21.05%	4	±21%
	**Answered**	19	
	**Skipped**	36	

Linac Output Reference Point

Q1. What is the geometry which defines the output reference point calibrated to be 1 cGy/MU at your facility?

Answer Choices	Responses		Uncertainty
SSD at Dmax	61.11%	77	±8%
SSD at D5	0.00%	0	±5%
SSD at D10	1.59%	2	±5%
SAD at Dmax	28.57%	36	±8%
SAD at D5	4.76%	6	±7%
SAD at D10	3.97%	5	±6%
Other (please specify)		4	
	**Answered**	126	
	**Skipped**	4	

Linac SRS Questions

Q1. How do you verify the patient alignment when using tables angles other than 0/180?

Answer Choices	Responses		Uncertainty
Rely on the couch isocentricity	32.76%	19	±13%
BrainLab ExacTrac	41.38%	24	±13%
Vision RT	20.69%	12	±13%
C-Rad Catalyst	0.00%	0	±7%

Other (please specify)	5.17%	3	±10%
	**Answered**	58	
	**Skipped**	0	

Q3. What is the leaf width of your MLC?

Answer Choices	Responses		Uncertainty
5 mm	31.03%	18	±12%
2.5 mm	63.79%	37	±12%
Other (please specify)	5.17%	3	±9%
	**Answered**	58	
	**Skipped**	0	

Q4. Do you ever use cones for SRS?

Answer Choices	Responses		Uncertainty
Yes	41.38%	24	±13%
No	58.62%	34	±13%
	**Answered**	58	
	**Skipped**	0	

Lung SBRT Options

Q1. Which immobilization system would you purchase today for your Lung SBRT program?

Answer Choices	Responses		Uncertainty
CDR LB-SBRT	4.80%	6	±7%
CIVCO ProLok	44.80%	56	±9%
Elekta BodyFix	20.80%	26	±9%
Qfix Stradivarius	13.60%	17	±9%
None	16.00%	20	±9%
Other (please specify)		22	
	**Answered**	125	
	**Skipped**	16	

Q2. Which breath control system would you purchase for your Lung SBRT program?

Answer Choices	Responses		Uncertainty
Abdominal Compression Plate	30.30%	40	±8%
Abdominal Compression Belt	17.42%	23	±8%
Active Breathing Control	4.55%	6	±7%
None with Gating	14.39%	19	±8%
None without Gating	15.91%	21	±8%
Other (please specify)	17.42%	23	±8%
	**Answered**	132	
	**Skipped**	9	

Q3. Which treatment technique would you use for Lung SBRT?

Answer Choices	Responses		Uncertainty
3DCRT	15.08%	19	±9%
IMRT	8.73%	11	±9%
VMAT/RapidArc	54.76%	69	±9%
Tomotherapy	1.59%	2	±5%
Other (please specify)	19.84%	25	±9%
	**Answered**	126	
	**Skipped**	15	

Lymph Node Bed PTV Contouring

Q1. Do you contour the Head/Neck Lymph Node Beds which the radiation oncologist then reviews and revises as needed?

Answer Choices	Responses		Uncertainty
Yes	12.50%	10	±7%
No	87.50%	70	±7%
Other (please specify)		4	
	**Answered**	80	
	**Skipped**	1	

Q2. Do you contour Pelvic Lymph Node Beds which the radiation oncologist then reviews and revises if needed?

Answer Choices	Responses		Uncertainty
Yes	16.25%	13	±7%
No	83.75%	67	±7%
Other (please specify)		4	
	**Answered**	80	
	**Skipped**	1	

Q3. Do you contour the Bowel Bag which the radiation oncologist then reviews and revises as needed?

Answer Choices	Responses		Uncertainty
Yes	65.43%	53	±10%
No	34.57%	28	±10%
Other (please specify)		5	
	**Answered**	81	
	**Skipped**	0	

Q4. Do you contour the Lumpectomy Bed which the radiation oncologist then reviews and revises as needed?

Answer Choices	Responses		Uncertainty
Yes	29.63%	24	±10%
No	70.37%	57	±10%
Other (please specify)		2	
	**Answered**	81	
	**Skipped**	0	

Mixed Energy Breast Tangents

Q1. Suppose you are using mixed energy tangents (6X and 15X) and Forward Planned Field-In-Field beams to plan a large breast. Which beams would you modulate? Please explain why is the Other field.

Answer Choices	Responses		Uncertainty
Leave 6X open and modulate the 15X	37.70%	23	±13%

Modulate the 6X and leave the 15X open	31.15%	19	±13%
Modulate both the 6X and 15X	31.15%	19	±13%
Other (please specify)		22	
	**Answered**	61	
	**Skipped**	5	

Monthly Linac QA Methods

Q1. How do you verify the linac output when performing monthly QA?

Answer Choices	Responses		Uncertainty
Ion chamber in water tank (primary TG-51 system)	20.13%	30	±7%
Ion chamber in solid water phantom (secondary system)	75.84%	113	±7%
Ion chamber array (secondary system)	1.34%	2	±4%
Diode array (secondary system)	0.67%	1	±4%
Other (please specify)	2.01%	3	±5%
	**Answered**	149	
	**Skipped**	0	

Q4. How do you verify energy on a monthly basis?

Answer Choices	Responses		Uncertainty
Two depth measurement in water	18.37%	27	±8%
Two depth measurement in solid water	63.27%	93	±8%
Two depth measurement using an	4.08%	6	±6%

ion chamber array or diode array			
Planar measurement to verify flatness (and therefore energy) is constant	10.20%	15	±8%
Other (please specify)	4.08%	6	±6%
	**Answered**	147	
	**Skipped**	2	

Q5. How do you verify flatness and symmetry on a monthly basis?

Answer Choices	Responses		Uncertainty
Use the results of the daily QA device	45.21%	66	±9%
Physics does their own measurement	35.62%	52	±9%
Other (please specify)	19.18%	28	±9%
	**Answered**	146	
	**Skipped**	3	

More questions regarding Lung 4DCT – revised

Q1. How do you create your Lung ITV?

Answer Choices	Responses		Uncertainty
Binned Datasets from 4DCT	82.04%	137	±5%
Full Inspiration - Full Expiration	11.98%	20	±5%
"Slow Scan"	5.99%	10	±5%
Linac CBCT	0.00%	0	±3%
Other (please specify)		8	
	**Answered**	167	
	**Skipped**	5	

Q2. Once you have created your ITV, how do you manage its Density?

Answer Choices	Responses		Uncertainty
Accept Free Breathing scan density	57.67%	94	±8%
Use Average Intensity Projection density	38.04%	62	±8%
Set density to 1	1.23%	2	±6%
Set density to GTV density	3.07%	5	±6%
Other (please specify)		8	
	**Answered**	164	
	**Skipped**	8	

Q3. How do you treat the PTV expansion?

Answer Choices	Responses		Uncertainty
Accept Free Breathing scan density	57.67%	94	±8%
Use Average Intensity Projection density	38.04%	62	±8%
Set density to 1	1.23%	2	±5%
Set density to GTV density	3.07%	5	±6%
Other (please specify)		8	
	**Answered**	163	
	**Skipped**	9	

Q4. How do you gate during Lung SBRT treatments?

Answer Choices	Responses		Uncertainty
Do not gate treatments	78.40%	127	±6%
Infrared tracking (eg RPM)	16.67%	27	±6%
Surface tracking (eg Vision RT)	1.85%	3	±4%

Fiducials and real time imaging (eg Calypso)	3.09%	5	±5%
Other (please specify)		15	
	**Answered**	162	
	**Skipped**	10	

Q5. What treatment modality do you primarily use for Lung SBRT?

Answer Choices	Responses		Uncertainty
Linac 3D	33.33%	56	±8%
Linac IMRT	10.71%	18	±8%
Linac VMAT	38.69%	65	±8%
Linac FFF	9.52%	16	±8%
CyberKnife	6.55%	11	±7%
Tomo	1.19%	2	±5%
Other (please specify)		23	
	**Answered**	168	
	**Skipped**	4	

Motion Management for Breast Radiotherapy

Q1. Do you use any type of motion management for breast radiotherapy?

Answer Choices	Responses		Uncertainty
Yes	80.00%	44	±10%
No	20.00%	11	±10%
Other (please specify)	0.00%	0	±5%
	**Answered**	55	
	**Skipped**	0	

Q2. If yes, what technique do you use?

Answer Choices	Responses		Uncertainty
Anzai Belt	0.00%	0	±7%
Anzai Laser	0.00%	0	±7%

Calypso Surface Beacons	6.82%	3	±11%
C-Rad Catalyst	6.82%	3	±11%
CyberKnife	0.00%	0	±7%
Elekta Active Breathing Coordinator	4.55%	2	±10%
HumediQ Identity	0.00%	0	±7%
Medspira Breath Hold ES	4.55%	2	±10%
Qfix SDX	2.27%	1	±9%
Varian RPM or RMM	43.18%	19	±14%
Vision RT AlignRT	22.73%	10	±14%
Other (please specify)	9.09%	4	±12%
	**Answered**	44	
	**Skipped**	11	

Q3. If yes, what Gating method do you use?

Answer Choices	Responses		Uncertainty
We do not gate	12.20%	5	±14%
Direct interface with linac	60.98%	25	±14%
Therapist manual control	21.95%	9	±14%
Other (please specify)	4.88%	2	±11%
	**Answered**	41	
	**Skipped**	14	

Q4. If yes, what feedback method do you use?

Answer Choices	Responses		Uncertainty
We do not use feedback	11.63%	5	±14%
Verbal coaching only	51.16%	22	±15%

Verbal coaching with visual feedback	27.91%	12	±15%
Visual feedback only	9.30%	4	±12%
Other (please specify)	0.00%	0	±8%
	**Answered**	43	
	**Skipped**	12	

Q5. If yes, what patients are treated with motion management?

Answer Choices	Responses		Uncertainty
Left breast only	86.36%	38	±9%
Both left and right breasts	4.55%	2	±8%
Other (please specify)	9.09%	4	±9%
	**Answered**	44	
	**Skipped**	11	

Motion Management for Breast Radiotherapy – meddos

Q1. Do you use any type of motion management for breast radiotherapy?

Answer Choices	Responses		Uncertainty
Yes	61.11%	11	±21%
No	33.33%	6	±21%
Other (please specify)	5.56%	1	±15%
	**Answered**	18	
	**Skipped**	1	

Q2. If yes, what technique do you use?

Answer Choices	Responses		Uncertainty
Anzai Belt	0.00%	0	±16%
Anzai Laser	0.00%	0	±16%
Calypso Surface Beacons	0.00%	0	±16%
C-Rad Catalyst	0.00%	0	±16%

CyberKnife	0.00%	0	±16%
Elekta Active Breathing Coordinator	0.00%	0	±16%
HumediQ Identity	0.00%	0	±16%
Medspira Breath Hold ES	0.00%	0	±16%
Qfix SDX	0.00%	0	±16%
Varian RPM or RMM	58.33%	7	±25%
Vision RT AlignRT	33.33%	4	±25%
Other (please specify)	8.33%	1	±21%
	**Answered**	12	
	**Skipped**	7	

Q3. If yes, what Gating method do you use?

Answer Choices	Responses		Uncertainty
We do not gate	40.00%	6	±25%
Direct interface with linac	33.33%	5	±25%
Therapist manual control	20.00%	3	±25%
Other (please specify)	6.67%	1	±18%
	**Answered**	15	
	**Skipped**	4	

Q4. If yes, what feedback method do you use?

Answer Choices	Responses		Uncertainty
We do not use feedback	8.33%	1	±18%
Verbal coaching only	50.00%	6	±26%
Verbal coaching with visual feedback	41.67%	5	±26%
Visual feedback only	0.00%	0	±13%

Other (please specify)	0.00%	0	±13%
	**Answered**	12	
	**Skipped**	7	

Q5. If yes, what patients are treated with motion management?

Answer Choices	Responses		Uncertainty
Left breast only	91.67%	11	±8%
Both left and right breasts	8.33%	1	±11%
Other (please specify)	0.00%	0	±7%
	**Answered**	12	
	**Skipped**	7	

MU validation

Q1. What MU Validation software do you use?

Answer Choices	Responses		Uncertainty
IMSure	11.11%	8	±11%
MUCheck	15.28%	11	±11%
RadCalc	59.72%	43	±11%
In-house	11.11%	8	±11%
None	2.78%	2	±7%
Other (please specify)		6	
	**Answered**	72	
	**Skipped**	1	

Q2. Would you recommend others get what you use?

Answer Choices	Responses		Uncertainty
Yes	76.81%	53	±9%
No	23.19%	16	±9%
	**Answered**	69	
	**Skipped**	4	

Multimodality Composite Plans

Q1. Have you ever generated a composite plan with XRT plus SRS? Please explain your answer in the Comment field.

Answer Choices	Responses		Uncertainty
No	62.50%	30	±13%
Yes	37.50%	18	±13%
Comment		10	
	**Answered**	48	
	**Skipped**	0	

Q2. Have you ever generated a composite plan with XRT and SBRT? Please explain your answer in the Comment field.

Answer Choices	Responses		Uncertainty
No	41.30%	19	±14%
Yes	58.70%	27	±14%
Comment		14	
	**Answered**	46	
	**Skipped**	2	

Q3. Have you ever generated a composite plan with XRT and HDR? Please explain your answer in Comment field.

Answer Choices	Responses		Uncertainty
No	63.64%	28	±14%
Yes	36.36%	16	±14%
Comment		12	
	**Answered**	42	
	**Skipped**	6	

Q4. Have you ever generated a composite plan with XRT and LDR? Please explain your answer in the Comment field.

Answer Choices	Responses		Uncertainty
No	88.10%	37	±9%
Yes	11.90%	5	±9%
Comment		7	

**Answered**	44
**Skipped**	4

Q5. Have you ever generated a composite plan with HDR and LDR? Please explain your answer in the Comment field.

Answer Choices	Responses		Uncertainty
No	100.00%	41	±0%
Yes	0.00%	0	±2%
Comment		3	
	**Answered**	41	
	**Skipped**	7	

Patient Specific QA for SRS

Q1. Do you perform patient specific QA for linac based SRS cases using 3D plans? (This includes plans using static or rotational conformal beams. It excludes plans using IMRT or VMAT beams.)

Answer Choices	Responses		Uncertainty
Yes	46.59%	41	±11%
No	45.45%	40	±11%
Other (please specify)	7.95%	7	±10%
	**Answered**	88	
	**Skipped**	1	

Patient Specific VMAT QA

Q1. Based on your experience and/or investigation, which system would your purchase today for Patient Specific VMAT QA?

Answer Choices	Responses		Uncertainty
Stationary 2D Detector Based - IBA Matrixx	0.00%	0	±5%
Stationary 2D Detector Based - PTW Octavius	1.10%	1	±6%
Stationary 2D Detector Based - Sun Nuclear MapCheck	10.99%	10	±10%

Gantry Mounted 2D Detector Based - IBQ Matrix	0.00%	0	±5%
Gantry Mounted 2D Detector Based - PTW Octavius	0.00%	0	±5%
Gantry Mounted 2D Detector Based - Sun Nuclear MapCheck	3.30%	3	±7%
3D Detector Based - IBA Matrixx Evolution	3.30%	3	±7%
3D Detector Based - Scandidose Delta 4	13.19%	12	±10%
3D Detector Based - Sun Nuclear ArcCheck	50.55%	46	±10%
EPID Based - EPIdos Epiqa	2.20%	2	±6%
EPID Based - IBA OmniPro	0.00%	0	±5%
EPID Based - Math Resolutions Dosimetry Check	6.59%	6	±9%
EPID Based - Sun Nuclear EPIDose	0.00%	0	±5%
Film Based - Ashland FilmQA Pro	0.00%	0	±5%
Film Based - IBA OmniPro	1.10%	1	±6%
Film Based - RIT	2.20%	2	±6%
Other	5.49%	5	±8%
Other (please specify)		7	
	**Answered**	91	
	**Skipped**	1	

Patient Specific VMAT QA Policy

Q1. What is you policy for Patient Specific VMAT QA?

Answer Choices	Responses		Uncertainty
Patient specific VMAT QA is not required	0.00%	0	±3%
Must be completed before first fraction	90.20%	92	±5%
Must be completed before second fraction	1.96%	2	±3%
Must be completed before third fraction	5.88%	6	±5%
Other	1.96%	2	±3%
Other (please specify)		5	
	**Answered**	102	
	**Skipped**	0	

Patients per linac

Q1. How much time do you schedule for routine patient treatments?

Answer Choices	Responses		Uncertainty
10 minutes	11.84%	9	±8%
12 minutes	7.89%	6	±8%
15 minutes	80.26%	61	±8%
Other (please specify)		9	
	**Answered**	76	
	**Skipped**	3	

Q2. How much time do you schedule for routine new patient starts?

Answer Choices	Responses		Uncertainty
15 minutes	16.44%	12	±9%
30 minutes	75.34%	55	±9%
45 minutes	5.48%	4	±8%
60 minutes	2.74%	2	±6%
Other (please specify)		10	

**Answered**	73
**Skipped**	6

Q3. How many routine patients per day do you treat on one linac?

Answer Choices	Responses		Uncertainty
10	1.28%	1	±6%
15	6.41%	5	±9%
20	15.38%	12	±11%
25	39.74%	31	±11%
30	20.51%	16	±11%
35	8.97%	7	±10%
40	5.13%	4	±8%
45	1.28%	1	±6%
50	1.28%	1	±6%
Other (please specify)		8	
	**Answered**	78	
	**Skipped**	1	

Perturbation Factors for SRS

Q1. Are you aware of the recently recommended Perturbation Factors pertaining to SRS dosimetry measurements?

Answer Choices	Responses		Uncertainty
Yes	50.00%	11	±20%
No	50.00%	11	±20%
Other (please specify)		0	
	**Answered**	22	
	**Skipped**	0	

Q2. Do you plan to apply these Perturbation Factors to your measured SRS data?

Answer Choices	Responses		Uncertainty
Yes	30.00%	6	±18%
No	70.00%	14	±18%

Other (please specify)		2
	**Answered**	20
	**Skipped**	2

Physician Presence at SRT

Q1. What does your facility require of the physician for the first SRT treatment?

Answer Choices	Responses		Uncertainty
Be present at the linac for the entire treatment	37.36%	34	±11%
Be present at the linac until the setup is confirmed	50.55%	46	±11%
Be available in the department	8.79%	8	±10%
No requirement to be available	0.00%	0	±6%
Other (please specify)	3.30%	3	±7%
	**Answered**	91	
	**Skipped**	0	

Q2. What does your facility require of the physician for the subsequent SRT treatments?

Answer Choices	Responses		Uncertainty
Be present at the linac for the entire treatment	20.88%	19	±10%
Be present at the linac until the setup is confirmed	52.75%	48	±10%
Be available in the department	18.68%	17	±10%
No requirement to be available	4.40%	4	±7%
Other (please specify)	3.30%	3	±7%
	**Answered**	91	

**Skipped**	0	

Q3. Does your facility believe it is a billing requirement for the physician to personally attend each SRT treatment?

Answer Choices	Responses		Uncertainty
Yes	49.40%	41	±11%
No	37.35%	31	±11%
Other (please specify)	13.25%	11	±11%
	**Answered**	83	
	**Skipped**	8	

Physician Supervision

Q1. In your operation, is the physician required to make a personal appearance at each and every simulation?

Answer Choices	Responses		Uncertainty
Yes	57.50%	23	±15%
No	42.50%	17	±15%
Other (please specify)		3	
	**Answered**	40	
	**Skipped**	1	

Physicist Presence at SRS/SRT

Q1. What does your facility require of the medical physicist for a SRS treatment?

Answer Choices	Responses		Uncertainty
Be present at the linac for the entire treatment	76.35%	113	±6%
Be present at the linac until the setup is confirmed	12.84%	19	±6%
Be available in the department	6.08%	9	±6%
No requirement to be available	2.70%	4	±5%

Other (please specify)	2.03%	3	±4%
	**Answered**	148	
	**Skipped**	0	

Q2. What does your facility require of the medical physicist for the first SRT treatment?

Answer Choices	Responses		Uncertainty
Be present at the linac for the entire treatment	71.62%	106	±7%
Be present at the linac until the setup is confirmed	16.89%	25	±7%
Be available in the department	4.73%	7	±6%
No requirement to be available	5.41%	8	±6%
Other (please specify)	1.35%	2	±4%
	**Answered**	148	
	**Skipped**	0	

Q3. What does your facility require of the medical physicist for the subsequent SRT treatments?

Answer Choices	Responses		Uncertainty
Be present at the linac for the entire treatment	46.62%	69	±8%
Be present at the linac until the setup is confirmed	15.54%	23	±8%
Be available in the department	24.32%	36	±8%
No requirement to be available	11.49%	17	±8%
Other (please specify)	2.03%	3	±5%
	**Answered**	148	
	**Skipped**	0	

Q4. Does your facility believe it is a billing requirement for a physicist to personally attend a SRS/SRT treatment?

Answer Choices	Responses		Uncertainty
Yes	26.24%	37	±8%
No	60.28%	85	±8%
Other (please specify)	13.48%	19	±8%
	**Answered**	141	
	**Skipped**	7	

Physicists as Medical Staff

Q1. Are Physicists included on the Medical Staff at your facility?

Answer Choices	Responses		Uncertainty
Yes	21.90%	23	±8%
No	74.29%	78	±8%
Other (please specify)	3.81%	4	±6%
	**Answered**	105	
	**Skipped**	0	

Portal Dosimetry for IMRT/VMAT QA – medphysusa

Q1. Do you use portal dosimetry for IMRT / VMAT QA?

Answer Choices	Responses		Uncertainty
Yes	55.77%	58	±10%
No	44.23%	46	±10%
Other (please specify)		8	
	**Answered**	104	
	**Skipped**	3	

Q2. Whether or not you use portal dosimetry, is an absolute dose measurement necessary?

Answer Choices	Responses		Uncertainty
On every patient	47.52%	48	±10%
Routinely	24.75%	25	±10%

Not at all	27.72%	28	±10%
Other (please specify)		19	
	**Answered**	101	
	**Skipped**	6	

Q3. Whether or not you use portal dosimetry, is it appropriate to bill for IMRT (77301) if the QA is done with portal dosimetry?

Answer Choices	Responses		Uncertainty
Yes, without any other measurement	70.10%	68	±9%
Yes, if an absolute dose measurement is done as well	22.68%	22	±9%
No	7.22%	7	±8%
Other (please specify)		10	
	**Answered**	97	
	**Skipped**	10	

Q4. If you use portal dosimetry, do you generate an absolute dose measurement?

Answer Choices	Responses		Uncertainty
Yes	56.06%	37	±12%
No	43.94%	29	±12%
Other (please specify)		10	
	**Answered**	66	
	**Skipped**	41	

Q5. If you use portal dosimetry, do you bill for IMRT (CPT 77301) without any other measurement?

Answer Choices	Responses		Uncertainty
Yes	84.21%	48	±9%
No	15.79%	9	±9%
Other (please specify)		13	
	**Answered**	57	
	**Skipped**	50	

Q6. If you use portal dosimetry, who takes the acquires the measurement?

Answer Choices	Responses		Uncertainty
Physicist	59.09%	39	±12%
Dosimetrist	9.09%	6	±11%
Therapist	31.82%	21	±12%
Other (please specify)		15	
	**Answered**	66	
	**Skipped**	41	

Portal Imaging and IGRT

Q1. For patients who get daily KV or CBCT IGRT, what is your policy regarding MV portal imaging for non-IMRT beams?

Answer Choices	Responses		Uncertainty
Each field once at start and never again	47.22%	17	±16%
Each field once at start and weekly thereafter	19.44%	7	±16%
Each field once at start and alternating fields thereafter	11.11%	4	±14%
Do not take MV portal images if daily IGRT is used	16.67%	6	±16%
Other (please specify)	5.56%	2	±12%
	**Answered**	36	
	**Skipped**	0	

Pregnant Physicists and Brachytherapy

Q1. What is your policy regarding pregnant physicists and prostate seed implant procedures?

Answer Choices	Responses		Uncertainty
Perform procedure with standard precautions	43.62%	41	±10%
Perform procedure with special	18.09%	17	±10%

precautions (please specify in the Comment field)			
Abstain from procedures during pregnancy	31.91%	30	±10%
Other (please specify in the Comment field)	6.38%	6	±9%
Comment		34	
	**Answered**	94	
	**Skipped**	2	

Q2. What is you policy regarding pregnant physicists and HDR procedures?

Answer Choices	Responses		Uncertainty
Perform procedure with standard precautions	35.11%	33	±11%
Perform procedure with special precautions (please specify in the Comment field)	22.34%	21	±11%
Abstain from procedures during pregnancy	31.91%	30	±11%
Other (please specify in the Comment field)	10.64%	10	±11%
Comment		38	
	**Answered**	94	
	**Skipped**	2	

Prone Breast Teletherapy

Q1. Do you treat some of your breast patients with a prone technique?

Answer Choices	Responses		Uncertainty
Yes	56.67%	17	±17%

No	43.33%	13	±17%
Other (please specify)		2	
	**Answered**	30	
	**Skipped**	2	

Q2. If yes, what system do you use?

Answer Choices	Responses		Uncertainty
Aktina	5.56%	1	±16%
Bionix	22.22%	4	±22%
CIVCO	44.44%	8	±22%
Orfit	5.56%	1	±16%
Qfix	16.67%	3	±22%
Homemade	5.56%	1	±16%
Other (please specify)		0	
	**Answered**	18	
	**Skipped**	14	

Q3. If yes, what percentage of pendulous breast patients who are candidates for a prone technique actually get treated with a prone breast technique?

Answer Choices	Responses		Uncertainty
0 – 33%	29.41%	5	±23%
34% - 66%	17.65%	3	±23%
67% - 100%	52.94%	9	±23%
Other (please specify)		0	
	**Answered**	17	
	**Skipped**	15	

Q4. How do you immobilize the breast for pendulous patients who are treated supine?

Answer Choices	Responses		Uncertainty
Breast Cup	28.57%	4	±24%
Thermoplastic Mesh	14.29%	2	±21%
Cloth Mesh	0.00%	0	±13%

Bra	57.14%	8	±24%
Other (please specify)		9	
	**Answered**	14	
	**Skipped**	18	

Prone Breast Teletherapy – 2015

Q1. Do you treat some of your breast patients with a prone technique?

Answer Choices	Responses		Uncertainty
Yes	43.59%	17	±15%
No	56.41%	22	±15%
Other (please specify)		1	
	**Answered**	39	
	**Skipped**	1	

Q2. If yes, what system do you use?

Answer Choices	Responses		Uncertainty
Aktina	0.00%	0	±14%
Bionix	25.00%	4	±23%
CIVCO	43.75%	7	±23%
Orfit	12.50%	2	±20%
Qfix	12.50%	2	±20%
Homemade	6.25%	1	±17%
Other (please specify)		1	
	**Answered**	16	
	**Skipped**	24	

Q3. If yes, which system would you prefer based on your experience?

Answer Choices	Responses		Uncertainty
Aktina	0.00%	0	±14%
Bionix	21.43%	3	±25%
CIVCO	35.71%	5	±25%

Orfit	21.43%	3	±25%
Qfix	14.29%	2	±21%
Homemade	7.14%	1	±18%
Other (please specify)		3	
	**Answered**	14	
	**Skipped**	26	

Q4. If yes, what percentage of pendulous breast patients who are candidates for a prone technique actually get treated with a prone breast technique?

Answer Choices	Responses		Uncertainty
0 - 33%	21.05%	4	±18%
34% - 66%	5.26%	1	±12%
67% - 100%	73.68%	14	±18%
Other (please specify)		0	
	**Answered**	19	
	**Skipped**	21	

Prostate IGRT with CBCT

Q1. For those of you with KV CBCT capability, how do you perform IGRT for the prostate?

Answer Choices	Responses		Uncertainty
CBCT without fiducial markers	53.66%	22	±15%
CBCT with fiducial markers	17.07%	7	±15%
Orthogonal KV/KV pair with fiducial markers	29.27%	12	±15%
Other (please specify)		12	
	**Answered**	41	
	**Skipped**	7	

Prostate Teletherapy Fractionation

Q1. What percentage of your prostate patients are treated with traditional fractionation (38-43 fractions)?

Answer Choices	Responses		Uncertainty
0% - 25%	15.79%	18	±8%
26% - 50%	6.14%	7	±8%
51% - 75%	13.16%	15	±8%
76% - 100%	64.91%	74	±8%
	**Answered**	114	
	**Skipped**	0	

Q2. What percentage of your prostate patients are treated with hypofractionation (20 - 27 fractions)?

Answer Choices	Responses		Uncertainty
0% - 25%	76.15%	83	±7%
26% - 50%	10.09%	11	±7%
51% - 75%	3.67%	4	±6%
76% - 100%	10.09%	11	±7%
	**Answered**	109	
	**Skipped**	5	

Q3. What percentage of your prostate patients are treated with SBRT (3 - 7 fractions)?

Answer Choices	Responses		Uncertainty
0% - 25%	91.96%	103	±4%
26% - 50%	4.46%	5	±4%
51% - 75%	1.79%	2	±3%
76% - 100%	1.79%	2	±3%
	**Answered**	112	
	**Skipped**	2	

Q4. Does your center use gel spacers for prostate teletherapy?

Answer Choices	Responses		Uncertainty
Yes	16.96%	19	±7%
No	79.46%	89	±7%
Other (please specify)	3.57%	4	±6%
	**Answered**	112	

**Skipped**	2

Prostate Teletherapy Fractionation – meddos

Q1. What percentage of your prostate patients are treated with traditional fractionation (38-43 fractions)?

Answer Choices	Responses		Uncertainty
0% - 25%	14.81%	4	±14%
26% - 50%	3.70%	1	±11%
51% - 75%	7.41%	2	±13%
76% - 100%	74.07%	20	±14%
	**Answered**	27	
	**Skipped**	0	

Q2. What percentage of your prostate patients are treated with hypofractionation (20 - 27 fractions)?

Answer Choices	Responses		Uncertainty
0% - 25%	81.48%	22	±13%
26% - 50%	11.11%	3	±13%
51% - 75%	0.00%	0	±7%
76% - 100%	7.41%	2	±11%
	**Answered**	27	
	**Skipped**	0	

Q3. What percentage of your prostate patients are treated with SBRT (3 - 7 fractions)?

Answer Choices	Responses		Uncertainty
0% - 25%	96.30%	26	±4%
26% - 50%	3.70%	1	±5%
51% - 75%	0.00%	0	±3%
76% - 100%	0.00%	0	±3%
	**Answered**	27	
	**Skipped**	0	

Q4. Does your center use gel spacers for prostate teletherapy?

Answer Choices	Responses		Uncertainty
Yes	7.69%	2	±10%
No	88.46%	23	±10%
Other (please specify)	3.85%	1	±8%
	**Answered**	26	
	**Skipped**	1	

QA for FIF beams

Q1. Is measurement based QA for Forward Planned Field In Field (FIF) beams performed in your department?

Answer Choices	Responses		Uncertainty
Yes	22.64%	12	±11%
No	77.36%	41	±11%
Other (please specify)		2	
	**Answered**	53	
	**Skipped**	0	

Q2. If measurement based QA for FIF beams is performed, what device is used?

Answer Choices	Responses		Uncertainty
MapCheck	42.11%	8	±22%
MatriXX	21.05%	4	±22%
Octavius	5.26%	1	±14%
Portal Dosimetry	31.58%	6	±22%
Other (please specify)		4	
	**Answered**	19	
	**Skipped**	34	

Q3. If measurement based QA for FIF is performed, who takes the measurements?

Answer Choices	Responses		Uncertainty
Physicist	82.35%	14	±15%
Dosimetrist	0.00%	0	±10%
Therapist	17.65%	3	±15%

Other (please specify)		5	
	**Answered**	17	
	**Skipped**	36	

Radiation Oncology Workload

Q1. In the past five years, how has the number of patients treated per year at your center changed?

Answer Choices	Responses		Uncertainty
Dropped more than 30%	16.16%	16	±10%
Dropped 20% - 30%	14.14%	14	±10%
Dropped 10% - 20%	23.23%	23	±10%
Constant within +/- 10%	28.28%	28	±10%
Increased 10% - 20%	10.10%	10	±10%
Increased 20% - 30%	2.02%	2	±6%
Increased more than 30%	6.06%	6	±8%
Other (please specify)		6	
	**Answered**	99	
	**Skipped**	1	

Q2. What do you believe is the reason for the change? (select all that apply)

Answer Choices	Responses		Uncertainty
Economy	52.11%	37	±6%
People do not have insurance to investigate medical symptoms	29.58%	21	±4%
Competing chemotherapy therapies	18.31%	13	±3%
Competing surgical therapies (DaVinci, etc)	25.35%	18	±4%

Competing targeted biotherapies	5.63%	4	±2%
Competing cyrotherapy	1.41%	1	±1%
Changes in screening guidelines (such as PSA)	15.49%	11	±3%
Competetion from nearby radiation oncology centers	71.83%	51	±7%
Other (please specify)		21	±4%
	**Answered**	71	
	**Skipped**	29	

Q3. Has the use of hypofractionation changed the number of fractions for prostate patients?

Answer Choices	Responses		Uncertainty
Yes	9.18%	9	±5%
No	90.82%	89	±5%
Other (please specify)		4	
	**Answered**	98	
	**Skipped**	2	

Q4. Has the use of hypofractionation changed the number of fractions for breast patients?

Answer Choices	Responses		Uncertainty
Yes	49.45%	45	±10%
No	50.55%	46	±10%
Other (please specify)		8	
	**Answered**	91	
	**Skipped**	9	

Q5. Has the use of hypfractionation changed the number of fractions for bone metastasis patients?

Answer Choices	Responses		Uncertainty
Yes	21.88%	21	±8%

No	78.13%	75	±8%
Other (please specify)		2	
	**Answered**	96	
	**Skipped**	4	

Q6. What is your forecast for patient load at your center in five years?

Answer Choices	Responses		Uncertainty
Drop > 30%	2.00%	2	±6%
Drop 20% - 30%	3.00%	3	±7%
Drop 10% - 20%	18.00%	18	±10%
Constant +/- 10%	49.00%	49	±10%
Increase 10% - 20%	19.00%	19	±10%
Increase 20% - 30%	7.00%	7	±9%
Increase > 30%	2.00%	2	±6%
	**Answered**	100	
	**Skipped**	0	

Rectal Balloon – meddos

Q1. How do you use a rectal balloon for prostate radiotherapy?

Answer Choices	Responses		Uncertainty
Do not use rectal balloon	75.76%	25	±13%
Use rectal balloon and fill it with water	15.15%	5	±13%
Use rectal balloon and fill it with air	9.09%	3	±12%
Other (please specify)		4	
	**Answered**	33	
	**Skipped**	0	

Rectal Balloon – medphys

Q1. How do you use a rectal balloon for prostate radiotherapy?

Answer Choices	Responses		Uncertainty
Do not use rectal balloon	76.92%	10	±19%
Use rectal balloon and fill it with water	15.38%	2	±20%
Use rectal balloon and fill it with air	7.69%	1	±16%
Other (please specify)		1	
	**Answered**	13	
	**Skipped**	1	

Rectal Filling Management for Prostate Patients

Q1. RTOG 0924 gives the following simulation innstructions for prostates. Which ones do you follow? (Check all that apply)

Answer Choices	Responses		Uncertainty
Do not use urethral contrast	64.29%	18	±8%
Do not use rectal contrast	78.57%	22	±8%
Bladder fullness should be reproducible	85.71%	24	±9%
Rectum as empty as possible	71.43%	20	±8%
Enema 1-2 hours before simulation	28.57%	8	±5%
	**Answered**	28	
	**Skipped**	1	

Q2. How do you align the prostate before each fraction?

Answer Choices	Responses		Uncertainty
CBCT without fiducial markers	41.38%	12	±18%
CBCT with fiducial markers	10.34%	3	±14%

KV-KV with fiducial markers	31.03%	9	±18%
MV-MV with fiducial markers	3.45%	1	±11%
ExacTrac with fiducial markers	3.45%	1	±11%
Calypso	6.90%	2	±12%
Rectal Balloon	3.45%	1	±11%
Align pelvic bones only	0.00%	0	±9%
No alignment verification	0.00%	0	±9%
Other (please specify)		3	
	**Answered**	29	
	**Skipped**	0	

Q3. For those who use CBCT, how do you manage the patient who has substantial rectal filling on a given day?

Answer Choices	Responses		Uncertainty
Get patient off table and have them evacuate	52.38%	11	±20%
Get patient off table and perform micro-enema	0.00%	0	±11%
Go ahead and treat	47.62%	10	±20%
Other (please specify)		2	
	**Answered**	21	
	**Skipped**	8	

Q4. For those who use CBCT, how do you manage the patient who has consistent rectal filling?

Answer Choices	Responses		Uncertainty
Continue with original plan	35.00%	7	±19%
Rescan and replan	65.00%	13	±19%

Other (please specify)		2	
	**Answered**	20	
	**Skipped**	9	

Replan for no bolus?

Q1. Consider the following. A chestwall is planned with daily 0.5 cm flab bolus for 28 fractions. After 25 fractions, the physician decides to to stop the bolus. Would you replan?

Answer Choices	Responses		Uncertainty
Yes	53.06%	26	±14%
No	46.94%	23	±14%
Other (please specify)		12	
	**Answered**	49	
	**Skipped**	3	

Q2. Consider the following. A chestwall is planned with daily 0.5 cm flab bolus for 28 fractions. After 25 fractions, the physician decides to to stop the bolus. Would you require the physician to write a whole new prescription?

Answer Choices	Responses		Uncertainty
Yes	44.68%	21	±14%
No	55.32%	26	±14%
Other (please specify)		17	
	**Answered**	47	
	**Skipped**	5	

SBRT Immobilization

Q1. Based on your experience, which immobilzation system would you purchase today for your Lung SBRT program? Please explain why in the "Other (please specify)" text box.

Answer Choices	Responses		Uncertainty
CDR LB-SBRT	6.78%	4	±10%
CIVCO ProLok	42.37%	25	±13%
Elekta BodyFix	25.42%	15	±13%
Qfix Stradivarius	3.39%	2	±9%
Other	22.03%	13	±13%

Other (please specify)		24	
	**Answered**	59	
	**Skipped**	2	

Setup Photos in Linac Vault

Q1. Do you include Setup Photos in your Field Displays so the therapists can view them in the Linac Vault?

Answer Choices	Responses		Uncertainty
Yes, with Aria	42.86%	12	±18%
Yes, with MOSAIQ	46.43%	13	±18%
No, with Aria	10.71%	3	±15%
No, with MOSAIQ	0.00%	0	±9%
	**Answered**	28	
	**Skipped**	0	

Sheets and Blankets and Pads - Oh My!

Q1. When treating the patient, what do you allow the therapists to place over the treatment area for either warmth or modesty?

Answer Choices	Responses		Uncertainty
Nothing	35.71%	10	±18%
One Sheet	50.00%	14	±18%
One Warm Blanket	14.29%	4	±16%
Other (please specify)		4	
	**Answered**	28	
	**Skipped**	3	

Q2. When treating the patient, what do you allow the therapists to place under the patient to improve comfort?

Answer Choices	Responses		Uncertainty
Nothing	6.90%	2	±13%
One sheet	44.83%	13	±18%
Thin Foam Pad	44.83%	13	±18%

Memory Foam Pad	3.45%	1	±11%
Other (please specify)		8	
	**Answered**	29	
	**Skipped**	2	

Q3. When treating chestwalls, how do you apply the bolus?

Answer Choices	Responses		Uncertainty
Directly on the Skin	32.26%	10	±16%
On top of Saran Wrap	64.52%	20	±16%
On top of Sheet	3.23%	1	±11%
Other (please specify)		3	
	**Answered**	31	
	**Skipped**	0	

Shoulder Immobilization

Q1. How do you immobilize the shoulders at the time of CT Simulation?

Answer Choices	Responses		Uncertainty
Head/Neck/Shoulder Mask	40.00%	12	±18%
Shoulder Suppression Brackets	16.67%	5	±18%
Wrist Rope Pulls with Foot Board	20.00%	6	±18%
Combination of Mask and Pulls	23.33%	7	±18%
Combination of Brackets and Pulls	0.00%	0	±9%
Other (please specify)		5	
	**Answered**	30	
	**Skipped**	3	

Q2. How do you immobilize the shoulders at the time of Treatment?

Answer Choices	Responses		Uncertainty

Head/Neck/Shoulder Mask	50.00%	15	±17%
Shoulder Suppression Brackets	16.67%	5	±17%
Wrist Rope Pulls with Foot Board	16.67%	5	±17%
Combination of Mask and Pulls	16.67%	5	±17%
Combination of Brackets and Pulls	0.00%	0	±9%
Other (please specify)		5	
	**Answered**	30	
	**Skipped**	3	

SIB for HN IMRT

Q1. Does your center use Simultaneous Integrated Boost for Head and Neck IMRT cases?

Answer Choices	Responses		Uncertainty
Yes	77.46%	55	±9%
No	22.54%	16	±9%
Other (please specify)		9	
	**Answered**	71	
	**Skipped**	4	

SIB for IMRT

Q1. Do you use Simultaneous Integrated Boost for your Head and Neck IMRT cases?

Answer Choices	Responses		Uncertainty
Yes	82.98%	39	±10%
No	17.02%	8	±10%
Other (please specify)		4	
	**Answered**	47	
	**Skipped**	1	

SIB for Prostate and PLN

Q1. Do you use Simultaneous Integrated Boost when treating prostate plus pelvic lymph nodes?

Answer Choices	Responses		Uncertainty
Yes	27.91%	12	±13%
No	72.09%	31	±13%
Other (please specify)		2	
	**Answered**	43	
	**Skipped**	1	

Signing Consent

Q1. At what time does the patient sign the Consent?

Answer Choices	Responses		Uncertainty
Initial Consultation	46.30%	25	±13%
Simulation	50.00%	27	±13%
First Fraction	3.70%	2	±9%
Other (please specify)		5	
	**Answered**	54	
	**Skipped**	1	

Q2. Who witnesses the signing of the Consent?

Answer Choices	Responses		Uncertainty
Physician	26.00%	13	±14%
Nurse	32.00%	16	±14%
Therapist	42.00%	21	±14%
Other (please specify)		8	
	**Answered**	50	
	**Skipped**	5	

Q3. What sort of Consent form do you use?

Answer Choices	Responses		Uncertainty
Facility Cengeral Consent Form	9.09%	5	±11%

Department General Consent Form	36.36%	20	±13%
Department Site Specific Consent Form	54.55%	30	±13%
Other (please specify)		2	
	**Answered**	55	
	**Skipped**	0	

SRS Dose Prescription

Q1. What dose does your rad onc prescribe for tumors less than 20 mm in maximum diameter?

Answer Choices	Responses		Uncertainty
27 Gy	0.00%	0	±11%
24 Gy	25.93%	7	±18%
21 Gy	18.52%	5	±18%
20 Gy	22.22%	6	±18%
18 Gy	14.81%	4	±18%
Other (please specify)	18.52%	5	±18%
	**Answered**	27	
	**Skipped**	0	

SRS Patient Alignment

Q1. How do you align your SRS patient?

Answer Choices	Responses		Uncertainty
Lasers	10.20%	5	±13%
OBI KV-KV imaging	14.29%	7	±14%
OBI CBCT imaging	42.86%	21	±14%
ExacTrac	30.61%	15	±14%
Vision RT	0.00%	0	±8%
C-RAD	2.04%	1	±9%
Other (please specify)		14	
	**Answered**	49	
	**Skipped**	5	

SRS: CT Contrast after MRI?

Q1. Let's say you SRS patient had MRI exam with contrast. Would you then get a CT scan for planning with contrast?

Answer Choices	Responses		Uncertainty
Yes	40.91%	18	±15%
No	52.27%	23	±15%
Other (please specify)	6.82%	3	±11%
	**Answered**	44	
	**Skipped**	0	

Static Field Shape Verification

Q1. Do you take initial portal images of the treatment fields with the patient on the table to verify the MLC shape for each static field?

Answer Choices	Responses		Uncertainty
Yes	90.20%	46	±7%
No	7.84%	4	±7%
Other (please specify)	1.96%	1	±5%
	**Answered**	51	
	**Skipped**	0	

Q2. After the patient has started, do you take subsequent portal images of the treatment fields?

Answer Choices	Responses		Uncertainty
Yes	68.63%	35	±12%
No	23.53%	12	±12%
Other (please specify)	7.84%	4	±10%
	**Answered**	51	
	**Skipped**	0	

Table Pads

Q1. For Brain patients, do you use a table pad?

Answer Choices	Responses		Uncertainty

Yes (please describe in Comment Field)	61.90%	13	±19%
No	38.10%	8	±19%
Please describe table pad		10	
	**Answered**	21	
	**Skipped**	0	

Q2. For Head/Neck patients, do you use a table pad?

Answer Choices	Responses		Uncertainty
Yes (please describe in Comment Field)	47.62%	10	±20%
No	52.38%	11	±20%
Please describe table pad		6	
	**Answered**	21	
	**Skipped**	0	

Q3. For Lung patients, do you use a table pad?

Answer Choices	Responses		Uncertainty
Yes (please describe in Comment Field)	23.81%	5	±16%
No	76.19%	16	±16%
Please describe table pad		4	
	**Answered**	21	
	**Skipped**	0	

Q4. For Pelvis patients, do you use a table pad?

Answer Choices	Responses		Uncertainty
Yes (please describe in Comment Field)	23.81%	5	±16%
No	76.19%	16	±16%

Please describe table pad		4
	**Answered**	21
	**Skipped**	0

Q5. For patients in pain, do you use a table pad?

Answer Choices	Responses		Uncertainty
Yes (please describe in Comment Field)	66.67%	14	±19%
No	0.00%	0	±11%
Other (please specify)	33.33%	7	±19%
	**Answered**	21	
	**Skipped**	0	

Target for 4DCT

Q1. How do you create your Internal Target Volume (ITV) for lung patients scanned with 4DCT?

Answer Choices	Responses		Uncertainty
Maximum Intensity Projection (MIP) of all phases	64.75%	79	±8%
Average Intensity Projection (AveIP) of all phases	4.92%	6	±7%
Inspiration + Expiration contours integrated	8.20%	10	±8%
Contour GTV on each individual phase and integrate over all phases	22.13%	27	±8%
Other (please specify)		19	
	**Answered**	122	
	**Skipped**	7	

TBI

Q1. What is the status of TBI at your facility?

Answer Choices	Responses		Uncertainty
Never did and don't plan to do TBI in the near future	11.27%	8	±8%
Did TBI in the past but have stopped	1.41%	1	±5%
Plan to start/resume TBI in the near future	5.63%	4	±8%
Continue to do TBI	80.28%	57	±8%
Other (please specify)	1.41%	1	±5%
	**Answered**	71	
	**Skipped**	0	

Q2. If you perform TBI, what is the beam arrangement?

Answer Choices	Responses		Uncertainty
AP/PA	46.77%	29	±13%
R/L Laterals	37.10%	23	±13%
Other (please specify)	16.13%	10	±13%
	**Answered**	62	
	**Skipped**	9	

Q3. If you do TBI, what is the patient position?

Answer Choices	Responses		Uncertainty
Standing	20.97%	13	±9%
Sitting	14.52%	9	±7%
Laying	66.13%	41	±11%
Other (please specify)	9.68%	6	±6%
	**Answered**	62	
	**Skipped**	9	

Q4. If you do TBI, what is your method of MU calculation?

Answer Choices	Responses		Uncertainty
Manual calculation	72.58%	45	±10%

TPS "IRREG" calculation	0.00%	0	±6%
TPS Isodose calculation	16.13%	10	±10%
"Test Dose" (calculate MU based on TLD measurements of low dose test treatment)	0.00%	0	±6%
Other (please specify)	11.29%	7	±10%
	**Answered**	62	
	**Skipped**	9	

Q5. If you do TBI, what is your In-Vivo Dosimetry status?

Answer Choices	Responses		Uncertainty
Do not use in-vivo dosimetry	16.39%	10	±13%
Use diodes	31.15%	19	±13%
Use MOSFETs	11.48%	7	±13%
Use TLD	9.84%	6	±12%
Other (please specify)	31.15%	19	±13%
	**Answered**	61	
	**Skipped**	10	

Q6. If you do TBI, what is your Compensator status? (choose all that apply)

Answer Choices	Responses		Uncertainty
Do not use compensators	19.35%	12	±7%
Use Lung compensators or partial transmission blocks	53.23%	33	±10%
Use Head compensator	29.03%	18	±8%
Other (please specify)	40.32%	25	±9%
	**Answered**	62	
	**Skipped**	9	

TBI Roles

Q1. For TBI, who participates in the initial simulation measurements? (check all that apply)

Answer Choices	Responses		Uncertainty
Physicist	70.83%	34	±10%
Dosimetrist	25.00%	12	±7%
Therapist	66.67%	32	±10%
Physician	20.83%	10	±6%
Other (please specify)	2.08%	1	±2%
	**Answered**	48	
	**Skipped**	0	

Q2. For TBI, who performs planning calculations? (check all that apply)

Answer Choices	Responses		Uncertainty
Physicist	89.58%	43	±10%
Dosimetrist	22.92%	11	±10%
Therapist	2.08%	1	±2%
Physician	2.08%	1	±2%
Other (please specify)	0.00%	0	±1%
	**Answered**	48	
	**Skipped**	0	

Q3. For TBI, who performs initial setup (including in-vivo measurements)? (check all that apply)

Answer Choices	Responses		Uncertainty
Physicist	79.17%	38	±10%
Dosimetrist	8.33%	4	±4%
Therapist	85.42%	41	±10%
Physician	10.42%	5	±4%
Other (please specify)	4.17%	2	±2%
	**Answered**	48	
	**Skipped**	0	

Q4. For TBI, who is present during each subsequent fraction? (check all that apply)

Answer Choices	Responses		Uncertainty
Physicist	60.42%	29	±9%
Dosimetrist	8.33%	4	±4%
Therapist	97.92%	47	±10%
Physician	18.75%	9	±5%
Other (please specify)	4.17%	2	±2%
	**Answered**	48	
	**Skipped**	0	

Testing for Linac Head Leakage

Q1. Do you test for Head Leakage when accepting a new linac?

Answer Choices	Responses		Uncertainty
Yes	53.13%	34	±12%
No	46.88%	30	±12%
	**Answered**	64	
	**Skipped**	0	

TG-51 Addendum

Q1. Has your clinic implemented the addendum to the TG-51 protocol (McEwen et al., Med. Phys. 2014)?

Answer Choices	Responses		Uncertainty
Yes	73.03%	65	±9%
No	26.97%	24	±9%
	**Answered**	89	
	**Skipped**	0	

Q2. If yes, what was the maximum calibration difference for any photon beam when switching from the original TG-51 protocol to the 2014 addendum?

Answer Choices	Responses		Uncertainty
< 1%	98.48%	65	±2%
> 1%	1.52%	1	±2%
	**Answered**	66	

**Skipped**	23

Therapeutic MPAs

Q1. Do you include MPAs in your practice (by that or some other name)?

Answer Choices	Responses		Uncertainty
Yes	24.00%	24	±8%
No	76.00%	76	±8%
Other (please specify)		3	
	**Answered**	100	
	**Skipped**	1	

Therapist Activities*

*For this survey, the uncertainty is calculated for each row.

Q1. Which of the following treatment preparation activities do your therapists perform?

	Yes		Uncertainty	No		Uncertainty	Total
Perform CT Simulation	96.74%	89	±3%	3.26%	3	±3%	92
Administer IV Contrast	52.81%	47	±10%	47.19%	42	±10%	89
Make Custom Bolus	78.89%	71	±8%	21.11%	19	±8%	90
Make Electron Blocks	69.23%	63	±9%	30.77%	28	±9%	91
Measure Electron Cutout Factors	12.22%	11	±6%	87.78%	79	±6%	90
Other (please specify)							10
						**Answered**	92
						**Skipped**	0

Q2. Which of the following QA activities do your therapists perform?

	Yes		Uncertainty	No		Uncertainty	Total
Daily/Weekly CT sim QA	80.43%	74	±8%	19.57%	18	±8%	92

Daily Linac QA	92.39%	85	±5%	7.61%	7	±5%	92
Daily IGRT QA	90.22%	83	±5%	9.78%	9	±5%	92
Shoot IMRT QA	22.83%	21	±8%	77.17%	71	±8%	92
Shoot Winston Lutz images	19.10%	17	±8%	80.90%	72	±8%	89
Other (please specify)							6
						**Answered**	92
						**Skipped**	0

Q3. Which of the following treatment related activities do your therapists perform?

	Yes		Uncertainty	No		Uncertainty	Total
Create treatment and imaging calendar in R&V	65.17%	58	±10%	34.83%	31	±10%	89
Perform TLD/Diode/MOSFET measurements	44.44%	40	±10%	55.56%	50	±10%	90
Other (please specify)							2
						**Answered**	90
						**Skipped**	2

Q4. Which of the following HDR related activities do your therapists perform?

	Yes		Uncertainty	No		Uncertainty	Total
Daily HDR QA	29.76%	25	±10%	70.24%	59	±10%	84
Deliver HDR treatments	48.19%	40	±11%	51.81%	43	±11%	83
Other (please specify)							8
						**Answered**	84
						**Skipped**	8

Q5. Which of the following weekly activities do your therapists perform?

	Yes		Uncertainty	No		Uncertainty	Total

Report changes in patient separation	79.78%	71	±8%	20.22%	18	±8%	89
Record SSDs	77.53%	69	±8%	22.47%	20	±8%	89
Check charts	61.8%	55	±10%	38.20%	34	±10%	89
Orchestrate the Chart Rounds	38.82%	33	±10%	61.18%	52	±10%	85
Other (please specify)							1
						**Answered**	90
						**Skipped**	2

Therapist Activities – meddos*

*For this survey, the uncertainty is calculated for each row.

Q1. Which of the following treatment preparation activities do your therapists perform?

	Yes		Uncertainty	No		Uncertainty	Total
Perform CT Simulation	90.74%	49	±7%	9.26%	5	±7%	54
Administer IV Contrast	23.53%	12	±11%	76.47%	39	±11%	51
Make Custom Bolus	67.31%	35	±12%	32.69%	17	±12%	52
Make Electron Blocks	75.47%	40	±11%	24.53%	13	±11%	53
Measure Electron Cutout Factors	18.87%	10	±10%	81.13%	43	±10%	53
Other (please specify)							0
						**Answered**	54
						**Skipped**	0

Q2. Which of the following QA activities do your therapists perform?

	Yes		Uncertainty	No		Uncertainty	Total

Daily/Weekly CT sim QA	75.93%	41	±11%	24.07%	13	±11%	54
Daily Linac QA	96.23%	51	±4%	3.77%	2	±4%	53
Daily IGRT QA	75.47%	40	±11%	24.53%	13	±11%	53
Shoot IMRT QA	13.46%	7	±8%	86.54%	45	±8%	52
Shoot Winston Lutz images	6.12%	3	±6%	93.88%	46	±5%	49
Other (please specify)							0
						**Answered**	54
						**Skipped**	0

Q3. Which of the following treatment related activities do your therapists perform?

	Yes		Uncertainty	No		Uncertainty	Total
Create treatment and imaging calendar in R&V	48.08%	25	±13%	51.93%	27	±13%	52
Perform TLD/Diode/MOSFET measurements	41.51%	22	±13%	58.49%	31	±13%	53
Other (please specify)							12
						**Answered**	53
						**Skipped**	1

Q4. Which of the following HDR related activities do your therapists perform?

	Yes		Uncertainty	No		Uncertainty	Total
Daily HDR QA	16.33%	8	±9%	83.67%	41	±9%	49
Deliver HDR treatments	26.53%	13	±12%	73.47%	36	±12%	49
Other (please specify)							15
						**Answered**	49
						**Skipped**	5

Q5. Which of the following weekly activities do your therapists perform?

	Yes		Uncertainty	No		Uncertainty	Total
Report changes in patient separation	72.22%	39	±11%	27.78%	15	±11%	54
Record SSDs	65.38%	34	±13%	34.62%	18	±13%	52
Check charts	50.94%	27	±13%	49.06%	26	±13%	53
Orchestrate the Chart Rounds	43.14%	22	±13%	56.86%	29	±13%	51
Other (please specify)							0
						**Answered**	54
						**Skipped**	0

Tomotherapy Independent MU Validation

Q1. If you operate a Tomotherapy unit, do you perform an independent MU validation?

Answer Choices	Responses		Uncertainty
Yes	15.15%	5	±12%
No	78.79%	26	±12%
Comment	6.06%	2	±9%
	**Answered**	33	
	**Skipped**	0	

TPS and OIS

Q1. Which of the following best describes your clinic?

Answer Choices	Responses		Uncertainty
Eclipse and ARIA	39.24%	62	±8%
Eclipse and MOSAIQ	10.76%	17	±8%
Pinnacle and ARIA	4.43%	7	±7%
Pinnacle and MOSAIQ	35.44%	56	±8%
Other TPS and ARIA	2.53%	4	±6%

Other TPS and MOSAIQ	7.59%	12	±8%
	**Answered**	158	
	**Skipped**	0	

Treating Facial Lesions

Q1. What modality do you use to treat facial lesions?

Answer Choices	Responses		Uncertainty
Electrons	80.00%	24	±13%
Superficial X-rays	20.00%	6	±13%
HDR with Ir-192	0.00%	0	±8%
HDR with electronic brachytherapy	0.00%	0	±8%
Other (please specify)		3	
	**Answered**	30	
	**Skipped**	1	

Q2. If using electrons, how do you shape the field?

Answer Choices	Responses		Uncertainty
Skin blocking	45.83%	11	±19%
Cutout in the electron cone	54.17%	13	±19%
Other (please specify)		10	
	**Answered**	24	
	**Skipped**	7	

Q3. If using skin blocking, what is your method?

Answer Choices	Responses		Uncertainty
Fabricate a custom lead mask by making a mould of the face and shaping lead	55.00%	11	±20%

Shape a piece of lead as best you can by hand	45.00%	9	±20%
Other (please specify)		3	
	**Answered**	20	
	**Skipped**	11	

Q4. If you make a lead mask, what material do you use to make the facial impression?

Answer Choices	Responses		Uncertainty
Thermoplastic sheet	40.00%	4	±27%
Geltrate	60.00%	6	±27%
Other (please specify)		6	
	**Answered**	10	
	**Skipped**	21	

Q5. If you make a lead mask, what material do you use to make the mould?

Answer Choices	Responses		Uncertainty
Dental Stone	70.00%	7	±25%
Cerrobend	30.00%	3	±25%
Other (please specify)		7	
	**Answered**	10	
	**Skipped**	21	

Treatment Plan Evaluation Methods

Q1. What method do you use to evaluate your treatment plans?

Answer Choices	Responses		Uncertainty
DVH Evaluator by DiversiLabs	0.00%	0	±5%
PlanIQ by Sun Nuclear	6.45%	6	±9%
Scorecard provided by treatment planning system	17.20%	16	±10%
Manual extraction of metrics which are	38.71%	36	±10%

tabulated in a spreadsheet			
None of the above	13.98%	13	±10%
Other (please specify)	23.66%	22	±10%
	**Answered**	93	
	**Skipped**	0	

Q2. What do you do with the results of the treatment plan evaluation? (select all that apply)

Answer Choices	Responses		Uncertainty
File report in patient chart	85.00%	68	±8%
Compile in database for comparison to other plans	8.75%	7	±5%
Compile in database for comparison to other treatment planners	2.50%	2	±2%
Other (please specify)	15.00%	12	±7%
	**Answered**	80	
	**Skipped**	13	

Treatment Plan QA

Q1. Let's say Dosimetrist A completes a treatment plan. Who reviews the plan prior the the physician?

Answer Choices	Responses		Uncertainty
Dosimetrist B	9.09%	4	±11%
Physicist	11.36%	5	±11%
Nobody	79.55%	35	±11%
Other (please specify)		6	
	**Answered**	44	
	**Skipped**	2	

Q2. After the physician approves the plan, who reviews the plan?

Answer Choices	Responses		Uncertainty

Dosimetrist B	5.00%	2	±5%
Physicist	95.00%	38	±5%
Other (please specify)		5	
	**Answered**	40	
	**Skipped**	6	

Q3. Who pushes the plan to the secondary MU validation program (e.g. RadCalc)?

Answer Choices	Responses		Uncertainty
Dosimetrist A	90.91%	40	±7%
Dosimetrist B	2.27%	1	±5%
Physicist	6.82%	3	±7%
Other (please specify)		2	
	**Answered**	44	
	**Skipped**	2	

Q4. Who calculates the secondary MU validation?

Answer Choices	Responses		Uncertainty
Dosimetrist A	84.44%	38	±9%
Dosimetrist B	2.22%	1	±6%
Physicist	13.33%	6	±9%
Other (please specify)		3	
	**Answered**	45	
	**Skipped**	1	

Q5. Who approves the secondary MU validation results?

Answer Choices	Responses		Uncertainty
Dosimetrist A	11.63%	5	±9%
Dosimetrist B	2.33%	1	±7%
Physicist	86.05%	37	±9%
Other (please specify)		5	
	**Answered**	43	
	**Skipped**	3	

Turnaround time for planning

Q1. In your department, what is the expected turnaround time for a physician to contour the CT dataset for a 3D plan?

Answer Choices	Responses		Uncertainty
Within one day	47.27%	26	±13%
Within two days	41.82%	23	±13%
Within three days	10.91%	6	±13%
Other (please specify)		16	
	**Answered**	55	
	**Skipped**	13	

Q2. In your department, what is the expected turnaround time for the dosimetrist to develop a 3D plan for the physician to review?

Answer Choices	Responses		Uncertainty
Within one day	30.51%	18	±13%
WIthin two days	42.37%	25	±13%
WIthin three days	27.12%	16	±13%
Other (please specify)		12	
	**Answered**	59	
	**Skipped**	9	

Q3. In your department, what is the expected turnaround time for a physician to contour the CT dataset for an IMRTplan?

Answer Choices	Responses		Uncertainty
Within one day	16.07%	9	±13%
Within two days	55.36%	31	±13%
Within three days	21.43%	12	±13%
Within four days	7.14%	4	±10%
Other (please specify)		14	
	**Answered**	56	
	**Skipped**	12	

Q4. In your department, what is the expected turnaround time for a dosimetrist to generate an IMRT plan for the physician to review?

Answer Choices	Responses		Uncertainty
Within one day	0.00%	0	±8%
Within two days	30.00%	15	±15%
Within three days	34.00%	17	±15%
Within four days	36.00%	18	±15%
Other (please specify)		16	
	**Answered**	50	
	**Skipped**	18	

VMAT SBRT using FFF Mode for Lung

Q1. Does your site treat SBRT lung using VMAT under FFF mode?

Answer Choices	Responses		Uncertainty
Yes	73.08%	57	±9%
No	24.36%	19	±9%
Other (please specify)	2.56%	2	±6%
	**Answered**	78	
	**Skipped**	0	

Q2. If Yes, do you reduce modulation during VMAT planning?

Answer Choices	Responses		Uncertainty
Yes	18.75%	12	±11%
No	67.19%	43	±11%
Other (please specify)	14.06%	9	±11%
	**Answered**	64	
	**Skipped**	14	

Q3. If Yes, do you limit dose rate when treating?

Answer Choices	Responses		Uncertainty
Yes	8.20%	5	±8%
No	85.25%	52	±8%

Other (please specify)	6.56%	4	±8%
	**Answered**	61	
	**Skipped**	17	

Q4. If Yes, do you use abdomen compression to limit respiration motion?

Answer Choices	Responses		Uncertainty
Yes	45.16%	28	±13%
No	43.55%	27	±13%
Other (please specify)	11.29%	7	±13%
	**Answered**	62	
	**Skipped**	16	

Q6. If No, why not?

Answer Choices	Responses		Uncertainty
Do not have a linac with FFF Mode	31.82%	7	±21%
Have a linac with FFF Mode but choose not to use it	36.36%	8	±21%
Other (please specify)	31.82%	7	±21%
	**Answered**	22	
	**Skipped**	56	

WBRT - Take 2

Q1. How do you immobilize your WBRT patients?

Answer Choices	Responses		Uncertainty
Thermoplastic Mask	100.00%	42	±0%
Masking Tape	0.00%	0	±2%
Other (please specify)	0.00%	0	±2%
	**Answered**	42	
	**Skipped**	0	

Q2. How do you plan your WBRT patients?

Answer Choices	Responses		Uncertainty
Collimator at 0, MLC	69.05%	29	±13%
Angled Collimator, MLC	19.05%	8	±13%
Angled Collimator, Blocking Tray	0.00%	0	±7%
Other (please specify)	11.90%	5	±13%
	**Answered**	42	
	**Skipped**	0	

Q3. What do you charge for WBRT planning?

Answer Choices	Responses		Uncertainty
Simple Simulation plus Simple Plan	12.20%	5	±14%
Complex Simulation plus Complex Plan	36.59%	15	±16%
3D Simulation	36.59%	15	±16%
Other (please specify)	14.63%	6	±16%
	**Answered**	41	
	**Skipped**	1	

Q4. What do you charge for the WBRT treatment?

Answer Choices	Responses		Uncertainty
Simple Treatment	18.42%	7	±14%
Complex Treatment	68.42%	26	±14%
Other (please specify)	13.16%	5	±14%
	**Answered**	38	
	**Skipped**	4	

Q6. Do you perform daily IGRT for your WBRT patients?

Answer Choices	Responses		Uncertainty
Yes	0.00%	0	±5%
No	100.00%	15	±0%

Other (please specify)	0.00%	0	±5%
	**Answered**	15	
	**Skipped**	27	

Weekly Rounds

Q1. What is the nature of your Weekly Rounds? (select all that apply)

Answer Choices	Responses		Uncertainty
Review every patient under treatment	34.48%	10	±10%
Review New Starts ONLY	58.62%	17	±12%
Review Boosts	41.38%	12	±11%
Review Brachytherapy patients	17.24%	5	±7%
We do not conduct Weekly Rounds	6.90%	2	±4%
Other (please specify)	24.14%	7	±8%
	**Answered**	29	
	**Skipped**	0	

Who Attends Simulation?

Q1. When do your radiation oncologists attend simulation for their patients?

Answer Choices	Responses		Uncertainty
As often as possible	58.54%	24	±15%
Only for select patients with special considerations	36.59%	15	±15%
Rarely, they write explicit instructions instead	4.88%	2	±11%
Other (please specify)		14	
	**Answered**	41	
	**Skipped**	6	

Q3. When do your dosimetrists attend simulation?

Answer Choices	Responses		Uncertainty
As often as possible	20.00%	9	±15%
Only for select patients with special considerations	46.67%	21	±15%
Rarely, they let the simulation therapist do the job	33.33%	15	±15%
Other (please specify)		8	
	**Answered**	45	
	**Skipped**	2	

Who does what – 2016

Q1. At your center, who routinely does the image fusion?

Answer Choices	Responses		Uncertainty
Physicist	7.02%	4	±6%
Dosimetrist	92.98%	53	±6%
Other (please specify)		8	
	**Answered**	57	
	**Skipped**	8	

Q2. At your center, who routinely does the IMRT/VMAT planning?

Answer Choices	Responses		Uncertainty
Physicist	3.17%	2	±3%
Dosimetrist	96.83%	61	±3%
Other (please specify)		2	
	**Answered**	63	
	**Skipped**	2	

Q3. At your center, who routinely does the SBRT planning?

Answer Choices	Responses		Uncertainty
Physicist	26.92%	14	±11%

Dosimetrist	73.08%	38	±11%
Other (please specify)		8	
	**Answered**	52	
	**Skipped**	13	

Q4. At your center, who routinely does the SRS planning?

Answer Choices	Responses		Uncertainty
Physicist	67.39%	31	±13%
Dosimetrist	32.61%	15	±13%
Other (please specify)		14	
	**Answered**	46	
	**Skipped**	19	

Q5. At your center, who routinely does the HDR planning?

Answer Choices	Responses		Uncertainty
Physicist	77.36%	41	±11%
Dosimetrist	22.64%	12	±11%
Other (please specify)		9	
	**Answered**	53	
	**Skipped**	12	

Q6. At your center, who routinely does the Prostate Seed Implant planning?

Answer Choices	Responses		Uncertainty
Physicist	73.91%	34	±12%
Dosimetrist	26.09%	12	±12%
Other (please specify)		9	
	**Answered**	46	
	**Skipped**	19	

Q7. At your center, who routinely does the Weekly Chart Check?

Answer Choices	Responses		Uncertainty
Physicist	94.74%	54	±4%

Dosimetrist	5.26%	3	±5%
Other (please specify)		8	
	**Answered**	57	
	**Skipped**	8	

Q8. At your center, who rotuinely does the extra work required for protocol patients?

Answer Choices	Responses		Uncertainty
Physicist	27.08%	13	±12%
Dosimetrist	72.92%	35	±12%
Other (please specify)		10	
	**Answered**	48	
	**Skipped**	17	

Who does what for HDR?

Q1. Who is responsible for taking measurements at Simulation?

Answer Choices	Responses		Uncertainty
Physician	5.13%	2	±11%
Physicist	58.97%	23	±15%
Dosimetrist	10.26%	4	±14%
Therapist	25.64%	10	±15%
Other (please specify)		3	
	**Answered**	39	
	**Skipped**	1	

Q2. Who is responsible for generating the HDR treatment plan?

Answer Choices	Responses		Uncertainty
Physician	2.70%	1	±10%
Physicist	59.46%	22	±15%
Dosimetrist	37.84%	14	±15%
Other (please specify)		5	
	**Answered**	37	
	**Skipped**	3	

Q3. Who is responsible for connecting the transfer tubes?

Answer Choices	Responses		Uncertainty
Physician	15.79%	6	±16%
Physicist	44.74%	17	±16%
Dosimetrist	2.63%	1	±9%
Therapist	36.84%	14	±16%
Other (please specify)		4	
	**Answered**	38	
	**Skipped**	2	

Q4. Who is responsible for initiating treatment at the Control Console?

Answer Choices	Responses		Uncertainty
Physician	28.95%	11	±15%
Physicist (not allowed in many States)	10.53%	4	±14%
Dosimetrist	5.26%	2	±11%
Therapist	55.26%	21	±15%
Other (please specify)		2	
	**Answered**	38	
	**Skipped**	2	

Who does what?

Q1. At your center, who routinely does the IMRT planning?

Answer Choices	Responses		Uncertainty
Physicist	5.93%	7	±4%
Dosimetrist	94.07%	111	±4%
Other (please specify)		2	
	**Answered**	118	
	**Skipped**	0	

Q2. At your center, who routinely does the SBRT planning?

Answer Choices	Responses		Uncertainty
Physicist	18.18%	18	±7%
Dosimetrist	81.82%	81	±7%
Other (please specify)		17	
	**Answered**	99	
	**Skipped**	19	

Q3. At your center, who routinely does the SRS planning?

Answer Choices	Responses		Uncertainty
Physicist	50.00%	45	±10%
Dosimetrist	50.00%	45	±10%
Other (please specify)		24	
	**Answered**	90	
	**Skipped**	28	

Q4. At your center, who routinely does the HDR planning?

Answer Choices	Responses		Uncertainty
Physicist	69.89%	65	±9%
Dosimetrist	30.11%	28	±9%
Other (please specify)		21	
	**Answered**	93	
	**Skipped**	25	

Q5. At your center, who routinely does the Prostate Seed Implant planning?

Answer Choices	Responses		Uncertainty
Physicist	65.63%	63	±9%
Dosimetrist	34.38%	33	±9%
Other (please specify)		17	
	**Answered**	96	
	**Skipped**	22	

Whole Breast Contouring – meddos

Q1. Inverse Planning for Whole Breast requires a Whole Breast PTV contour. At your facility, who creates the Whole Breast PTV contour?

Answer Choices	Responses		Uncertainty
Radiation Oncologist	34.62%	9	±17%
Dosimetrist	65.38%	17	±17%
Other (please specify)		3	
	**Answered**	26	
	**Skipped**	3	

Q2. How is the Whole Breast PTV contour created?

Answer Choices	Responses		Uncertainty
From scratch following an Atlas such as from RTOG	53.85%	14	±18%
Apply open Tangents and convert an Isodose Line to the PTV contour	46.15%	12	±18%
Other (please specify)		5	
	**Answered**	26	
	**Skipped**	3	

Winston Lutz for Lung SBRT

Q1. Do you perform a WInston Lutz test before each Lung SBRT fraction?

Answer Choices	Responses		Uncertainty
Yes	21.43%	9	±11%
No	78.57%	33	±11%
Other (please specify)		3	
	**Answered**	42	
	**Skipped**	2	

Winston Lutz Test

Q1. For SRS treatments, when do you perform the Winston Lutz test?

Answer Choices	Responses	Uncertainty

The night before the treatment	7.14%	7	±9%
The morning of the treatment	52.04%	51	±10%
Immediately before the treatment	40.82%	40	±10%
Other (please specify)		18	
	**Answered**	98	
	**Skipped**	16	

Q2. For SRT or SBRT treatments, when do you perform the Winston Lutz test?

Answer Choices	Responses		Uncertainty
The night before the treatment	10.53%	8	±10%
The morning of the treatment	69.74%	53	±10%
Immediately before the treatment	19.74%	15	±10%
Other (please specify)		38	
	**Answered**	76	
	**Skipped**	38	

Q3. Who performs the WInston Lutz test?

Answer Choices	Responses		Uncertainty
Physicist	77.27%	85	±7%
Dosimetrist	2.73%	3	±5%
Therapist	20.00%	22	±7%
Other (please specify)		9	
	**Answered**	110	
	**Skipped**	4	

Q4. Does the test use film or the portal imager?

Answer Choices	Responses		Uncertainty

Film	25.23%	28	±8%
Portal Imager	74.77%	83	±8%
Other (please specify)		6	
	**Answered**	111	
	**Skipped**	3	

Q5. How are the images analyzed?

Answer Choices	Responses		Uncertainty
Manually	44.55%	45	±10%
Mobius	24.75%	25	±10%
PIPSpro	16.83%	17	±10%
RIT	13.86%	14	±10%
Other (please specify)		17	
	**Answered**	101	
	**Skipped**	13	

Q6. If using Cones and MLC the same day, which Winston Lutz test(s) do you perform?

Answer Choices	Responses		Uncertainty
Cone alone	41.25%	33	±11%
MLC alone	20.00%	16	±11%
Both	38.75%	31	±11%
Other (please specify)		29	
	**Answered**	80	
	**Skipped**	34	

Q7. What happens if IGRT (MV, KV, CBCT) indicates the anatomy does not line up?

Answer Choices	Responses		Uncertainty
Go with the Laser setup	7.00%	7	±4%
Shift based on IGRT	93.00%	93	±4%
Other (please specify)		16	
	**Answered**	100	

**Skipped**	14

Would you invest in hetero brachy software?

Q1. Would you purchase an algorithm that supports heterogeneity corrected dose calculations in HDR brachytherapy (e.g.Acuros BV) - today?

Answer Choices	Responses		Uncertainty
Yes	54.17%	26	±14%
No	45.83%	22	±14%
	**Answered**	48	
	**Skipped**	1	
